# Combining network pharmacology, molecular docking, molecular dynamics simulation, and experimental verification to examine the efficacy and immunoregulation mechanism of FHB granules on vitiligo

**DOI:** 10.3389/fimmu.2023.1194823

**Published:** 2023-07-27

**Authors:** Xiaolong Li, Fengze Miao, Rujuan Xin, Zongguang Tai, Huijun Pan, Hao Huang, Junxia Yu, Zhongjian Chen, Quangang Zhu

**Affiliations:** ^1^ Shanghai Skin Disease Hospital, Tongji University School of Medicine, Shanghai, China; ^2^ Shanghai Engineering Research Center for Topical Chinese Medicine, Shanghai, China

**Keywords:** Fufang Honghua Buji granules, vitiligo, network pharmacology, molecular docking, molecular dynamics simulation, anti-inflammation, JAK-STAT pathway

## Abstract

**Background:**

Fufang Honghua Buji (FHB) granules, have proven efficacy against vitiligo in long-term clinical practice. However, its major active chemical components and molecular mechanisms of action remain unknown. The purpose of this study was to confirm the molecular mechanism of FHB’s therapeutic effect on vitiligo utilizing network pharmacology, molecular docking, and molecular dynamics simulation prediction, as well as experimental verification.

**Methods:**

Traditional Chinese Medicine Systems Pharmacology (TCMSP) and HERB databases were used to obtain the chemical composition and action targets of FHB. Online Mendelian Inheritance in Man (OMIM), DrugBank, DisGeNET, GeneCards, and Therapeutic Target Database (TTD) databases were applied to screen for vitiligo-related targets. Gene Ontology (GO) and Kyoto Encyclopedia of Genes and Genomes (KEGG) enrichment analyses were performed through the Matascape database. Molecular docking and dynamics simulation methods were for the analysis of the binding sites and binding energies between the FHB’s active components and the targets. Finally, a vitiligo mouse model was created, and the therapeutic effect and molecular mechanism of action of FHB were validated using enzyme linked immunosorbent assay (ELISA), western blot (WB), and quantitative reverse transcription-polymerase chain reaction (qRT-PCR). Additionally, hematoxylin-eosin staining (HE) and blood biochemical assays were conducted to assess the biosafety of FHB.

**Result:**

The screening of chemical composition and targets suggested that 94 genetic targets of FHB were associated with vitiligo. The bioinformatics analysis suggested that luteolin, quercetin, and wogonin may be major active components, and nuclear factor-kappa B p65 subunit (RELA), signal transducer, and activator of transcription (STAT) 3 and RAC-alpha serine/threonine-protein kinase (AKT) 1 may be potential targets of FHB-vitiligo therapy. Molecular docking and dynamics simulation further demonstrated that luteolin, quercetin, and wogonin all bound best to STAT3. Through experimental verification, FHB has been demonstrated to alleviate the pathogenic characteristics of vitiligo mice, suppress the JAK-STAT signaling pathway, reduce inflammation, and increase melanogenesis. The *in vivo* safety evaluation experiments also demonstrated the non-toxicity of FHB.

**Conclusions:**

FHB exerts anti-inflammatory and melanogenesis-promoting effects via the effect of multi-component on multi-target, among which the JAK-STAT pathway is a validated FHB-vitiligo target, providing new ideas and clues for the development of vitiligo therapy.

## 1 Introduction

Vitiligo, a common depigmentation illness, which affects between 0.1% and 2% of the world’s population, is defined by a selective loss of melanocytes that results in unsightly white patches on the skin (1). Vitiligo patients are typically between the ages of 10 and 30 (2, 3). Genetics account for around 80% of the condition, with environmental factors accounting for the remaining 20% (4). Vitiligo disfigurement lowers a patient’s quality of life by undermining their sense of self-worth and resulting in severe psychological distress, which is on par with the severity of other severe skin conditions like psoriasis and eczema (5). An immunological imbalance is the primary etiology of vitiligo, while it may also be accompanied by other variables such as oxidative stress, melanocyte defects, keratin-forming cell defects, and fibroblast defects (6, 7). Conventional therapies for vitiligo include PUVA therapy (application of psoralen followed by exposure to UV light or sunlight) and topical administration of corticosteroids and/or calcineurin inhibitors. These treatments, however, are not universally effective, and all existing treatments only provide short-term relief and do not recolor all lesion sites. Patients have a 40% recurrence incidence in the first year after discontinuing treatment, and these medicines can be difficult to apply (4, 8). More dangerously, these medicines can cause skin atrophy, phototoxic responses, rashes, capillary dilatation, migraines, and liver and kidney failure (9–11). As a result, it is critical to design more effective and safe treatments.

In recent years, traditional Chinese medicine (TCM) has gained popularity as a complementary alternative medicine around the world, with many herbal formulas successfully treating diseases such as cancer, diabetes, and novel coronavirus (12–14). TCM remedies are also widely employed in the treatment of dermatological illnesses, and some studies have shown the potential usefulness of TCM formulas in the treatment of skin pigmentation disorders in recent years (15). FHB is composed of 17 traditional Chinese medicinal ingredients, with safflower (*Carthamus tinctorius* L.), Fructus Psoraleae (*Psoralea corylifolia* L. seed), and puncture vine (dried ripe fruit of *Tribulus terrestris* L.) being the most important therapeutic components. Safflower is commonly used to promote blood circulation and cure blood stasis. The isolated safflower yellow (CY) from safflower has been proven to effectively reduce whole blood viscosity, plasma viscosity, and erythrocyte aggregation index (16), and safflower extract can also be utilized as an antioxidant to protect cells from oxidative damage (17). Psoralen is the main active component of Fructus Psoraleae, which has a wide range of biological activities for the treatment of osteoporosis, tumors, microbial infections, and inflammation, and is used in conjunction with UVA light to treat skin diseases such as psoriasis, eczema, and vitiligo (18, 19). Tribulus terrestris is commonly used to treat inflammation, dry skin, pruritus, and oxidative damage (20), and it has been shown to promote melanin production (21). Despite its long history of therapeutic use in China, the precise molecular mechanism of FHB therapy for vitiligo is unknown.

**Figure 1:**
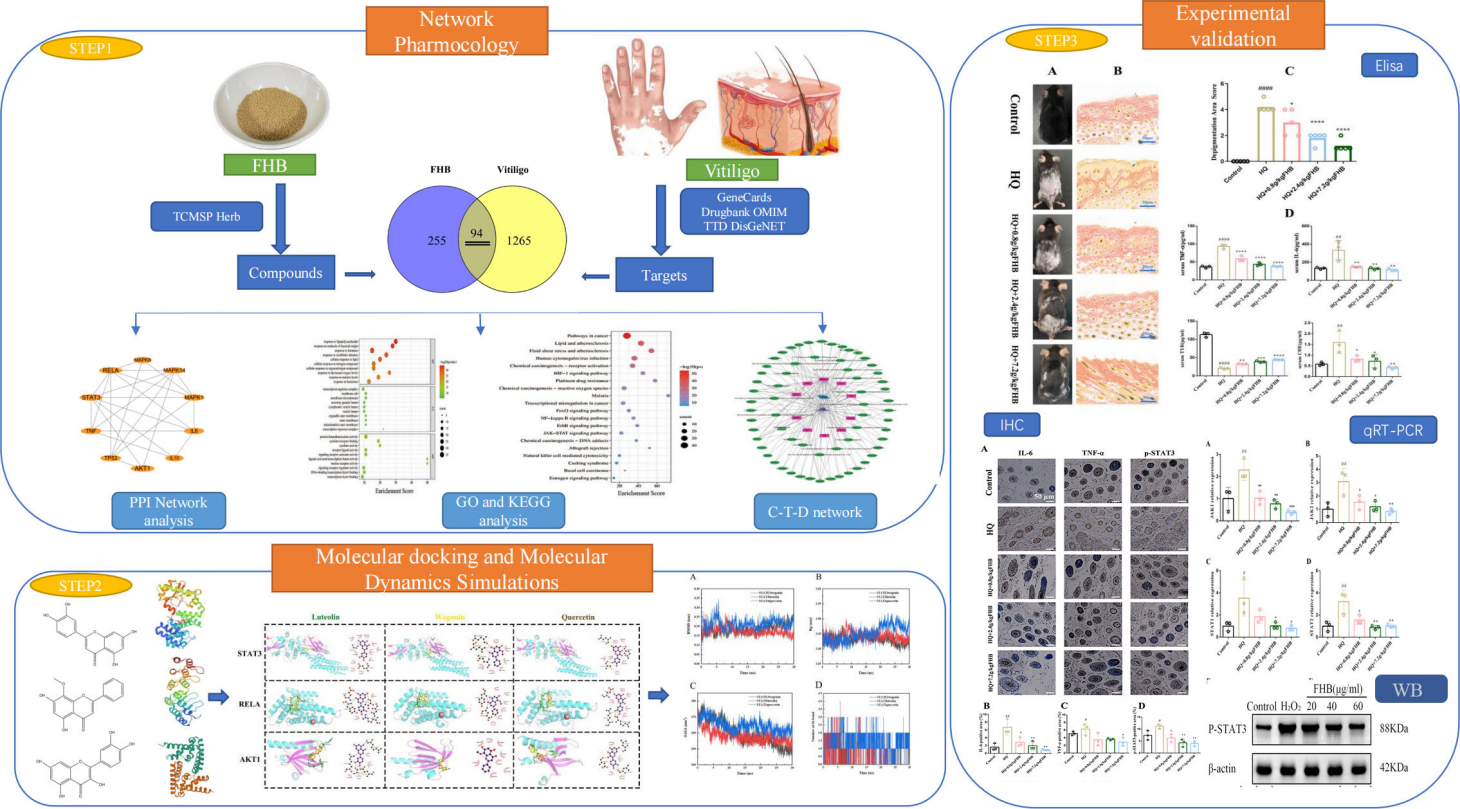
Workflow of network pharmacology, molecular docking, molecular dynamics simulation, and experimental verification for the therapeutic mechanism of FHB granules on vitiligo.

TCM formulas have a complicated chemical composition with multiple active targets. Network pharmacology, which is based on systems biology theory, employs network analysis and other techniques to reveal the complex network relationships between drug targets and diseases, providing a new approach to predicting the molecular mechanisms of TCM formulas, including multiple active components, contributing targets, and involved pathways (22). The simulation of ligand-receptor protein interaction via a computer platform is referred to as molecular docking and molecular dynamics simulation technology. In this study, the network pharmacology approach was used to construct a “component-target-disease” network for FHB, and the target functions and signaling pathways were investigated using gene ontology (GO) and Kyoto Encyclopedia of Genes and Genomes (KEGG) enrichment analysis and the binding ability of key components to key targets was tested using molecular docking and molecular dynamics simulation technology. Based on the findings of the “component-target-disease” network, GO and KEGG analysis, and molecular docking, we hypothesized that the STAT3 and JAK-STAT pathways may be involved in the therapeutic molecular mechanism of FHB in vitiligo. For further validation, pharmacological experiments were conducted to confirm the pharmacodynamic effects of FHB and determine whether the STAT3 and the JAK-STAT pathways are involved in its therapeutic molecular processes. This study aims to provide a theoretical foundation for further clinical practice and clinical research by exploring the therapeutic mechanism of FHB in the treatment of vitiligo. Scheme 1 represents the flow chart of our study (**Figure 1**).

## 2 Materials and methods

### 2.1 Materials and experimental animals

Traditional Chinese medicine (TCM) materials including safflower (*Carthamus tinctorius* L.), puncture vine (dried ripe fruit of *Tribulus terrestris* L.), dark plum (*Prunus mume* Sieb. et Zucc.), catnip (*Nepeta cataria* L.), Danshen (the dried root or rhizome of *Salvia miltiorrhiza* Bge.), Dangshen (the dried root or rhizome of *Codonopsis pilosula* (Franch.) Nannf.), siberian cocklebur fruit (the fruits of *Xanthium sibiricum* Patr.), licorice (*Glycyrrhiza uralensis* Fisch.), peach seed (*Prunus persica* L. Batsch), Danggui (the root or rhizome of *Angelica sinensis* (Oliv.) Diels), milkvetch root (*Astragalus membranceus* (Fisch.) Bunge.), Daxueteng (the liana stem of *Sargentodoxa cuneata* (Oliv.) Rehd. et Wils.), Saposhnikoviae Radix (the root or rhizome of *Saposhnikovia divaricata* (Trucz.) Schischk.), Herba Siegesbeckiae (*Siegesbeckia orientalis* L.), Fructus Psoraleae (*Psoralea corylifolia* L. seed), Heshouwu (the root or rhizome of *Fallopia multiflora* (Thunb.) Harald.), and magnetic powder (Fe_3_O_4_) were purchased from Shanghai Hongqiao Chinese Medicine Beverage Co., Ltd. Carboxymethylcellulose Sodium, hydroquinone (HQ) were bought from MACKLIN reagent, IL-6 mouse ELISA kit (030311WL202680304), TNF-α mouse ELISA kit (030311WL104840304) and TYR mouse ELISA kit (030311WL204410304) were from Jianglaibio,STAT3(bs-1141r), IL-6(bs-6309r), and TNF-α(bs-10802r) antibodies were procured from Affinity Biosciences, formalin, 4% Paraformaldehyde Fix Solution, RNA extraction solution was bought from Solarbio, and NovoScript Plus All-in-one 1st Strand cDNA Synthesis SuperMis (gDNA Purge) kit and NovoStart SYBR qPCR SuperMix Plus kit were purchased from Novoprotein, China. CCK8 (C0038) kit and H_2_O_2_ (9011M2114540) were purchased from Beyotime.

Twenty-five 6-week male C57BL/6 mice, weighing 20 to 22 g, were bought from Shanghai Sunrise Biotechnology Company. All mice were maintained in the animal laboratory of Shanghai Skin Disease Hospital at a temperature of 23 ± 2°C, with 16 h of light and 8 h of darkness, and provided with sterile water and standard feed. All animal procedures were approved and supervised by the Ethics Committee of Shanghai Skin Disease Hospital (ID number 2020-062).

### 2.2 Preparation of FHB granules

FHB granules are an oral TCM formula used in clinical treatments of vitiligo at the Shanghai Skin Disease Hospital. The TCM materials of Fufang Honghua Buji (FHB) granules - i.e., carthami flos (Carthamus tinctorius L.), psoraleae fructus (Psoralea corylifolia L.), tribuli fructus (Tribulus terrestris L.), salviae miltiorrhizae radix et rhizoma (Salvia miltiorrhiza Bge.), persicae semen (Prunus persica (L.) Batsch), angelicae sinensis radix (Angelica sinensis (Oliv.) Diels), glycyrrhizae radix et rhizoma (Glycyrrhiza uralensis Fisch.), codonopsis radix (Codonopsis pilosula (Franch.) Nannf.), polygoni multiflori radix (Polygonum multiforum Thunb.), magnetitum (Fe3O4), xanthii fructus (Xanthium sibiricum Patr.), saposhnikoviae Radix (Saposhnikovia divaricata (Trucz.) Schischk.), schizonepetae herba (Schizonepeta tenuifllia Briq.), siegesbeckiae herba (Siegesbeckia orientalis L.), mume fructus (Prunus mume (Sieb.) Sieb. et Zucc.), stagentodoxae caulis (Sargentodoxa cuneata (Oliv.) Rehd. et Wils.), and astragali radix (Astragalus membranceus (Fisch.) Bge.) were mixed in the ratio of 1.7: 1.7: 1.1: 1.1: 1.1: 1.1: 1.1: 1.1: 1.1: 1.1: 1.1: 1.1: 2.2: 2.2: 2.2. Then, the mixture was decocted with 10-fold weight of water for 2h, and was subsequently decocted with 8-fold weight of water for 1h. After two decoction processes, the filtrates after filtration by sterilized gauze were combined and were left to set for 24 h. After that, the supernatant was taken for concentration and evaporation using a rotatory evaporator. Finally, the concentrated extract was added with appropriate amounts of excipients (sucrose and dextrin with 2: 1.25), stirred well, and dried into granules. In our experiment, due to the low concentration of the granules after brewing, we chose to gavage mice directly with the aqueous concentrated extract of FHB to achieve sufficient dosing concentration as well as to facilitate absorption in mice.

### 2.3 Screening of active compounds for FHB granules

The chemical composition of 17 herbs was obtained from Traditional Chinese Medicine Systems Pharmacology (TCMSP) (https://old.tcmsp-e.com/tcmsp.php) database and Herb database (http://herb.ac.cn/), and screened was also based on oral bioavailability (OB) and drug-like index (DL). The components meeting both OB>30% and DL≥0.18 were selected as potential active compounds (23, 24).

### 2.4 Predication of potential targets of FHB granules for vitiligo treatment

Target information of active compounds in FHB was collected from TCMSP and Herb databases. All FHB targets collected were combined and de-duplicated, then corrected on the Universal Protein database (https://www.uniprot.org/), and converted into the Official Gene Symbol of *Homo sapiens*. Vitiligo-related targets were obtained from the OMIM database (https://www.omim.org/), DrugBank database (https://www.drugbank.com/datasets), DisGeNET database (https://www.disgenet.org/), GeneCards database (https://www.genecards.org/) and TTD database (http://db.idrblab.net/ttd/) by searching “vitiligo” as the keyword and removing duplicate and false positive genes. FHB targets and vitiligo-related targets were processed using the Venny 2.1 (https://bioinfogp.cnb.csic.es/tools/venny/) online tool, and the intersection target genes of the two (FHB-vitiligo targets) were collected as the potential targets of FHB for vitiligo treatment.

### 2.5 Construction of FHB-vitiligo targets protein-protein interaction network

To further investigate the interaction of FHB involved in vitiligo-related targets, we imported the FHB-vitiligo targets into the STRING database (https://cn.string-db.org/), set the condition as human (*homo sapiens*), selected data with combinescore≥0.9 to imported into Cytoscape 3.7.2 (https://cytoscape.org/), and constructed the PPI network model. The Degree values of the topological parameter in the network were used to identify the core target genes in the network.

### 2.6 Gene Ontology and Kyoto Encyclopedia of Genes and Genomes enrichment analysis of the FHB-vitiligo targets

Metascape (https://metascape.org/gp/index.html#/main/step1) was utilized to perform enrichment analysis of FHB-vitiligo target genes. Metascape integrates KEGG pathways, GO biological processes, reactant gene sets, classical pathways, transcriptional factor targets, and other tools to annotate biological processes and pathway analysis. All genes are used as enrichment backgrounds. In addition, the systematic data are well-timed. A threshold of p < 0.05 was used to identify key GO and KEGG pathways.

### 2.7 Construction of “component-target-disease” network

To investigate the mechanism of action of FHB in vitiligo, the Cytoscape 3.7.2 software package was used to construct a “component-target-disease” network. This network helps to scientifically explain the complex relationships between compounds, genes, pathways, and diseases. An Analyzer plug-in is used to analyze the network diagram nodes.

### 2.8 Molecular docking

Based on the results of a previous network pharmacology screening, the 2- dimension (2D) structures of potential active compounds in FHB were downloaded from the PubChem database (https://pubchem.ncbi.nlm.nih.gov/), and the 3D structure of the target protein was obtained from the PDB database (https://pubchem.ncbi.nlm.nih.gov/). These files had been saved in PDB format. In AutoDock4, the active compound protein files were set as follows: water was removed and replaced with hydrogen, and the protein was designated as a receptor and stored as a PDBQT protein receptor file. The protein structures’ water molecules and small molecule ligands were removed using Pymol2 software, and the data were then imported into AutoDockTools1.1.2 for pre-processing of dehydration reaction and hydrogenation. To examine the junctions and activities of targets and FHB molecules, molecular docking was performed in AutoDock vina1.1.2.

### 2.9 Molecular dynamics simulation

Molecular dynamics simulations were performed using the GROMACS 5.0.4 software package for the receptor protein with the highest binding energy to the ligand molecule in the molecular docking results to obtain its binding mode and binding energy to the ligand. Taking the molecular docking results as the initial structure, the Energy minimization was done first, then 500 ps molecular dynamics simulations were done under vacuum conditions. The Coulomb force intercept and van der Waals radius intercept were both 1.4 nm, and finally the system was equilibrated using the regular system (NVT) and isothermal isobaric system (NPT), and then molecular dynamics simulations were performed for 30 ns at room temperature and pressure.

### 2.10 Experimental verification

#### 2.10.1 Mice *in vivo* model of HQ-induced vitiligo

Hydroquinone (HQ) was used in our experiment to induce vitiligo in mice. The back hair of all experimental mice was shaved over an area of 3cm × 2cm.

The mice were randomly divided into 5 groups, 5 mice each group (1): Control group: 1% Carboxymethylcellulose Sodium was applied to the shaved area on the back of the mice. (2) Hydroquinone (HQ) group: 5 mg 3% HQ ointment was evenly applied to the shaved area, and 0.4 ml of pure water was gavaged. (3) HQ+0.8g/kg FHB group: 5mg 3% HQ ointment was evenly applied to the shaved area, followed by the gavage of 0.4ml FHB (0.8g/kg). (4) HQ+ 2.4g/kg FHB group: 5mg 3% HQ ointment was evenly applied to the shaved area, and then 0.4ml FHB (2.4g/kg) was gavaged. (5) HQ+7.2g/kg FHB group: 5mg 3% HQ ointment was evenly applied to the shaved area, followed by the gavage of 0.4ml FHB (7.2g/kg). All the groups were administered once a day for 60 days. On day 60, the vitiligo area of each group (Control, HQ, HQ+0.8g/kg FHB, HQ+2.4g/kg FHB, HQ+7.2g/kg FHB) was imaged using a digital camera, and was evaluated according to the previous protocol: 0 points were labeled for the absence of depigmentation 0%; 1 point was labeled for 0 > 5%; 2 points were labeled for > 5–25%; 3 points were labeled for> 25–50%; 4 points were labeled for > 50–75%; 5 points were labeled for > 75–100% (25). Diet was prohibited before sampling, and the skin of the tithe logo area and blood samples were collected when the experiment was over on day 60.

#### 2.10.2 Cellular model of vitiligo and cell viability assay

B16 melanoma cells were purchased from the ATCC cell bank. To construct vitiligo model cells, cells were inoculated into 96-well plates at 100 μl (1x10^4^ cells) per well. The control group was incubated with 1640 medium and the H_2_O_2_ group was incubated with different concentrations of H_2_O_2_ solution (50, 100, 200, 400, 600μmol/L) for 2h, 4h, 6h, and 8h respectively. The viability of B16 cells was assayed at different periods using the cck-8 method.

To examine the *in vitro* therapeutic effect of FHB, different concentrations of FHB (5-200μg/ml) were used to treat vitiligo B16 cells (200μmol H_2_O_2_ for 6 hours). After 24h-incubation of FHB, the viabilities of vitiligo B16 cells were detected using cck-8. The control group was incubated with a mere 1640 medium for 30 h, and the H_2_O_2_ group was first incubated with 200μmol H_2_O_2_ for 6 hours, and then incubated with a mere 1640 medium for 24h.

#### 2.10.3 The enzyme-linked immunosorbent assay

Blood samples collected from the orbits were allowed to stand for 1 h at 4°C and then were centrifuged (1800 rpm/min) for 5 min at 4°C. The supernatant was collected and used for ELISA. IL-6, TNF-α, and TYR protein expression levels were detected using an IL-6 mouse ELISA kit, TNF-α mouse ELISA, kit, and TYR mouse ELISA kit.

#### 2.10.4 Histological and immunostaining analysis

The collected skin samples were fixed with 4% formalin solution, followed by melanin staining performed by Wuhan Service Technology Co., Ltd. 200 × microscopy was used to observe the pathological changes in the dorsal lesions, which were imaged using CaseViewer 2.2 scanning and viewing software.

For immunohistochemical staining analysis, the skin of the dorsal lesions was collected, fixed in 4% paraformaldehyde, and then immunohistochemical (IHC) staining was performed to analyze the expression of IL-6, TNF-α, and p-STAT3. Antibodies used for immunohistochemical staining included IL-6, TNF-α, and p-STAT3. The expressions of IL-6, TNF-α, and p-STAT3 were quantified based on positive areas within each square millimeter of the epidermis. The positive areas band intensities were quantified using Image J software (NIH, Bethesda, MD). Three sections per group were used for analysis.

#### 2.10.5 RNA extraction and qRT-PCR

The total RNA of vitiligo skin tissue on the back of mice was extracted using an RNA extraction solution according to the manufacturer’s instructions. According to the instructions of the NovoScript Plus All-in-one 1st Strand cDNA Synthesis SuperMis (gDNA Purge) kit, 1μg of RNA was utilized in reverse transcriptase reaction to gain cDNA.

The expression of Jak1, Jak2, Sta1, Stat2, Stat3, TNF-α, IL-6, and Tyr was investigated using RT-PCR. The primer sequences of the above genes are listed in **Table 1**. 2×NovoStart SYBR qPCR SuperMix Plus kit and LightCycler 480 System were employed to perform qRT-PCR. The threshold cycle (CT) is defined as the PCR cycle at which the fluorescent signal of the reporter dye crosses the automatically placed threshold. Genic transcript levels were calculated using the formula 2^-ΔΔCT^. The mRNA levels of tested genes were normalized glyceraldehyde-3-phosphate dehydrogenase (Gapdh) as an internal reference. The qRT-PCR experiment was conducted in biological triplicate for each group.

**Table 1:** Primers for qRT-PCR.

Gene	Sense (5′-3′)	bp	annealing temperatures (°C)
Gapdh-F	CCTCGTCCCGTAGACAAAATG	133	60
Gapdh-R	TGAGGTCAATGAAGGGGTCGT		60
Jak1-F	CGGAACTTCCCAAAGACATCA	153	60
Jak1-R	TCCAAGGTAGCCAGGTATTTCA		60
Jak2-F	CTTATTCATGGGAATGTGTGTGC	270	60
Jak2-R	ATCCAGAGCACTCAGGGGCTTA		60
Stat1-F	TGCCTATGATGTCTCGTTTGC	165	60
Stat1-R	ATCTGTACGGGATCTTCTTGGA		60
Stat2-F	AAGGCAGCGAATCACTCAAAG	153	60
Stat2-R	TCAAGAAGCCGAAGTCCCAAA		60
Stat3-F	TGCGGAGAAGCATTGTGAGTG	210	60
Stat3-R	TCTTAATTTGTTGGCGGGTCT		60
Tyr-F	TAACTTACTCAGCCCAGCATCC	113	60
Tyr-R	ATAGTGGTCCCTCAGGTGTTCC		60

#### 2.10.6 Western blot analysis

Cells and tissues were homogenized in a cell lysis reagent, lysed on ice for 30 minutes, and then centrifuged at 16000 g at 4°C for 5 minutes. A BCA protein assay kit was used to detect the total protein concentration of each sample. SDS-PAGE gels were used to grade the proteins, which were then blotted onto nitrocellulose membranes. Subsequently, nonspecific binding was blocked in TBST [0.05% Tween 20, 120 mmol/L Tris-HCl (pH 7.4) and 150 mmol/L NaCl] with 5% skim milk powder at 25°C for 1 h. The membranes were then incubated with the indicated primary antibodies (p-STAT3) at 4°C. Afterward, the membranes were washed three times with TBST and inoculated with a secondary antibody. Visualize protein bands using the Bio-Rad Chemi Doc XRS+ imaging system. Band intensities were quantified using Image J software (NIH, Bethesda, MD). All Western blot assays were performed in triplicate.

#### 2.10.7 *In vivo* biosafety evaluation

Twenty mice were randomly divided into Control and FHB groups, with 10 mice in each group. To make 0.75g/ml of FHB aqueous extract, 0.3g of the dried FHB aqueous extract powder was weighed and combined with 0.4 ml of pure water. The administration group was given 0.5 mL of 0.75 g/mL FHB per day and the blank group was given the same volume of saline for 14 days. After treatment, mice were euthanized, and the blood of the mice was collected. The plasma was sent for a hematology survey (n = 5), and the serum was separated for biochemistry analysis (n = 5). The main organs were taken from the mice and processed into sections for HE staining. 200 x microscope to observe the pathological changes of each organ.

### 2.11 Statistical methods

GraphPad Prism 8.0 software (San Diego, CA, USA) was used for analysis and graphing. The data are presented as the mean standard ± standard error of the mean. To compare various groups, a one-way analysis of variance (ANOVA) was utilized. The statistical significance level was set at p<0.05.

## 3 Results

### 3.1 Acquisition of potential FHB action targets for vitiligo therapy

**Figure 2:**
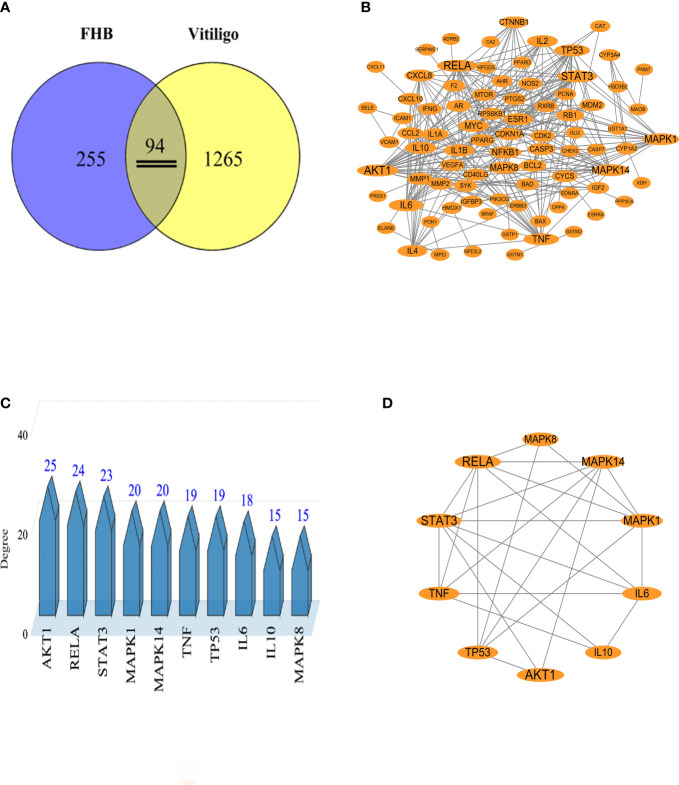
Analysis of potential FHB action targets for vitiligo therapy. **(A)** Venn diagram of potential FHB action targets for vitiligo therapy. **(B)** Protein-protein interaction (PPI) network of potential FHB action targets. The circles represent the target protein. The larger the diameter of the circle, the larger the Degree value. Straight lines indicate the interaction between target proteins. **(C)** Top 10 targets of FHB action targets for vitiligo therapy with the highest Degree values. **(D)** Core PPI network of core proteins extracted from **(B)**.

The chemical components of FHB were gathered from the TCMSP and Herb databases, and the screening conditions were set as OB ≥ 30% and DL ≥ 0.18. After removing repeated and false-positive components and targets, a total of 291 active components and 349 potential FHB action targets were predicted (**Supplementary Tables 1**, **2**). To search and integrate vitiligo-related targets, the OMIM database, DrugBank database, DisGeNET database, GeneCards database, and TTD database were used. Repeated targets were removed, and a total of 1359 vitiligo-related target proteins were obtained to be closely related to the development of vitiligo. The FHB action targets were cross-referenced with vitiligo disease targets, yielding a total of 94 target genes (**Figure 2A**).

### 3.2 PPI network analysis

To create a PPI network, 94 potential FHB action targets for vitiligo were uploaded to the STRING database, and the system was configured as follows: selecting “Homo sapiens” in the species column, choosing “Evidence” in the network edge column, and setting the confidence level to > 0.9. A total of 94 target nodes and 296 lines were collected in the PPI network of FHB-vitiligo targets, showing the intricate interconnection between these targets (**Figure 2B**).

We calculated the average degree value and selected targets larger than the average degree value as the core targets. The top core 10 targets with the highest Degree values were presented as the key targets of FHB for vitiligo therapy (**Figures 2C, D**). Among them, the Degree values of RAC-alpha serine/threonine-protein kinase (AKT1), nuclear factor-kappa B p65 subunit (RELA, signal transducer and activator of transcription 3 (STAT3) were highest and were respectively 25, 24, and 23 (**Supplementary Table 4**). The Top 10 targets also included two inflammatory factor targets, i.e., tumor necrosis factor (TNF, often called TNF-α) and interleukin (IL)-6, with Degree values of 19 and 18, respectively.

### 3.3 GO and KEGG analysis

**Figure 3:**
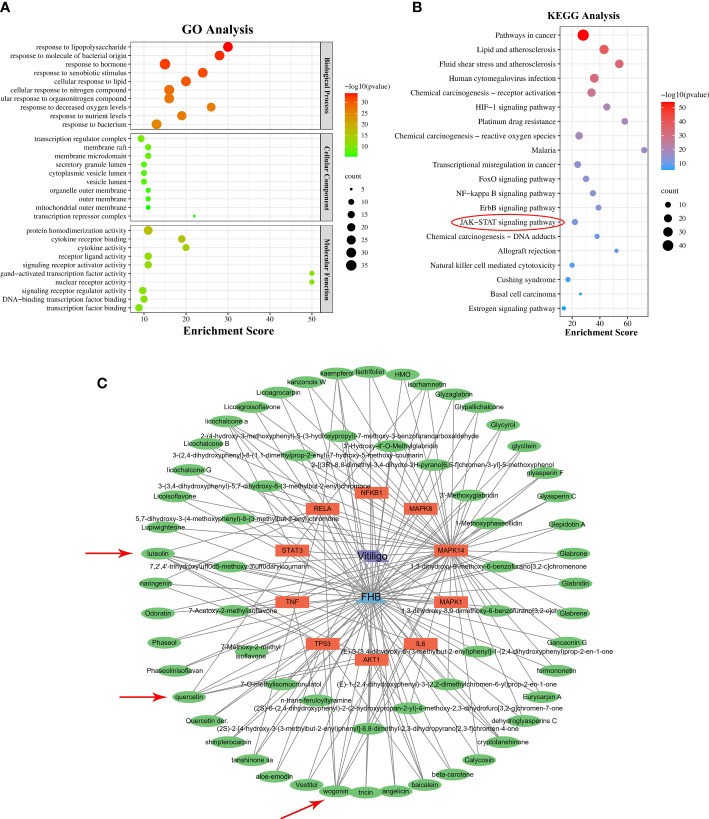
GO and KEGG analysis, and “component-target-disease” network. **(A)** The top 10 significantly enriched terms (P < 0.05) in biological process, cellular component, and molecular function of GO analysis were selected. The X-axis represents the enrichment score. **(B)** The top 20 significantly enriched pathways (P < 0.05) were selected. The X-axis represents the enrichment score. Red circles represent the main signaling pathways explored in this study. **(C)** “Component-target-disease” network. The blue triangle represents FHB, the green ovals represent the active components of FHB, the orange rectangles represent FHB-vitiligo targets, and the purple parallelogram represents vitiligo. Red arrows represent FHB core ingredients.

The biological properties of FHB-vitiligo targets and the signaling pathways involved were investigated using GO and KEGG analysis. A total of 1525 biological processes, 49 cellular components, and 116 molecular functions were enriched by GO analysis (**Supplementary Table 5**), and the top 10 of each were chosen for presentation after being ranked by corrected P-values. According to the GO analysis, FHB-vitiligo targets may participate the biological processes such as “response to lipopolysaccharide”, “cellular response to nitrogen compound”, and “response to decreased oxygen levels”; influence on the cellular components including “transcription regulator complex” and “transcription repressor complex”; and exert molecular functions like “cytokine receptor binding”, “cytokine activity”, and “nuclear receptor activity” (**Figure 3A**).

KEGG analysis enriched 180 pathways (**Supplementary Table 6**). According to the findings of the KEGG analysis, the mechanism of FHB for vitiligo therapy may involve pathways including “pathways in cancer”, “HIF-1 signaling pathway”, “FoxO signaling pathway”, “NF-kappa B signaling pathway”, “ErbB signaling pathway”, and “JAK-STAT signaling pathway” (**Figure 3B**).

### 3.4 “Component-target-disease” network analysis and key active components of FHB for vitiligo therapy

To discover the molecular mechanism of FHB for vitiligo, we constructed the “component-target-disease” network based on the top 10 FHB-vitiligo targets predicted by the PPI network. As shown in **Figure 3C**, a total of 75 nodes and 159 lines were collected. Each active component corresponded to multiple targets, and each FHB-vitiligo target was linked to several components, illustrating the potential FHB mechanism of multi-component and multi-targeted treatment for vitiligo.

Next, we searched for key active components of FHB for vitiligo therapy based on the Degree values of components in the “component-target-disease” network. Luteolin, wogonin, quercetin, baicalein, naringenin, beta-carotene, kaempferol, licochalcone A, isorhamnetin, and cryptotanshinone were presented as top 10 components (**Table 2**). This implied that these FHB components may majorly act on key FHB-vitiligo targets and then influence the core pathways used to treat vitiligo. Among them, luteolin, wogonin, and quercetin had the highest Degree values, which were 7, 6, and 6, respectively (**Table 2**).

**Table 2:** Top 10 compounds information of FHB network.

Number	Compound	Molecule structure	OB	DL	Degree
1	luteolin	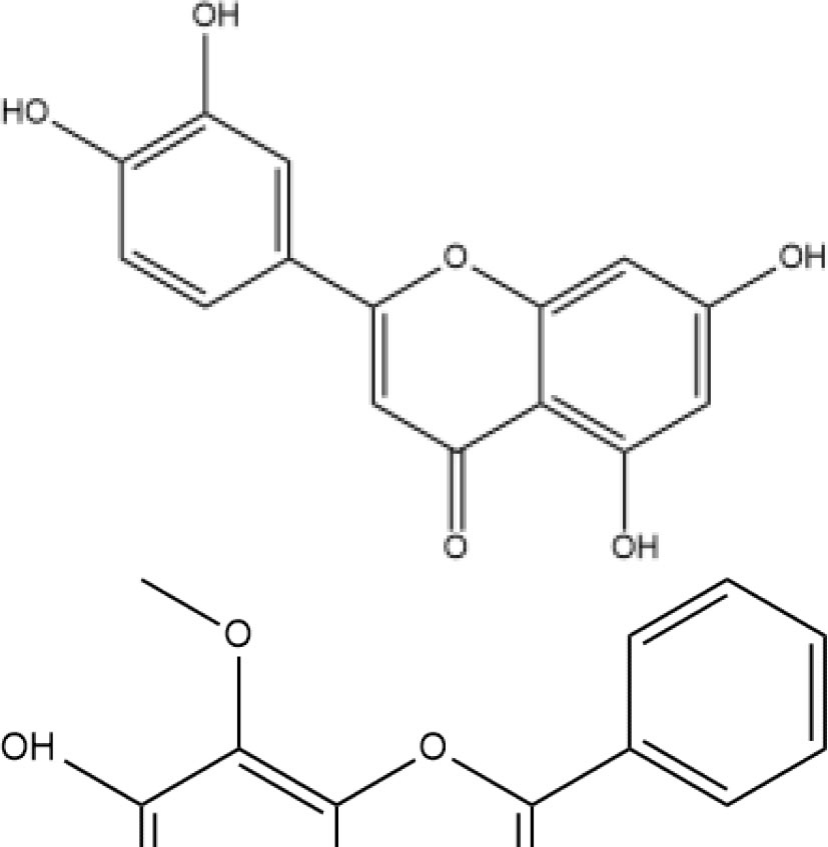	36.16	0.24	7
2	wogonin	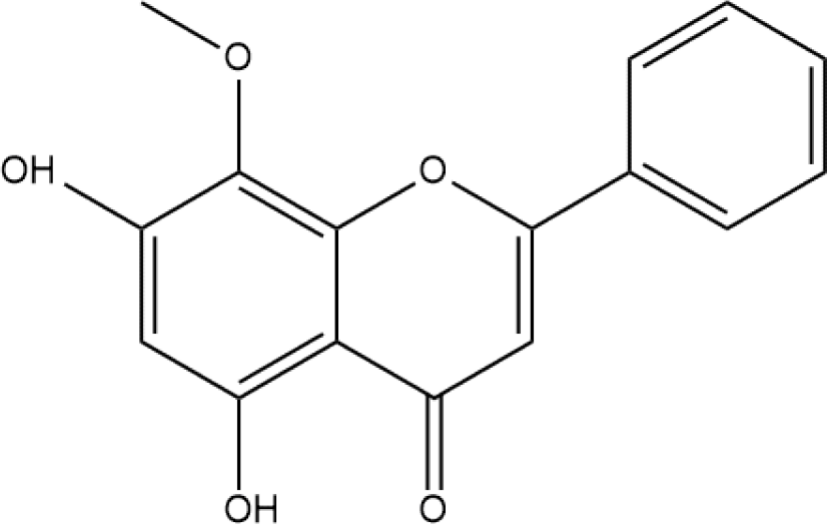	30.68	0.23	6
3	quercetin	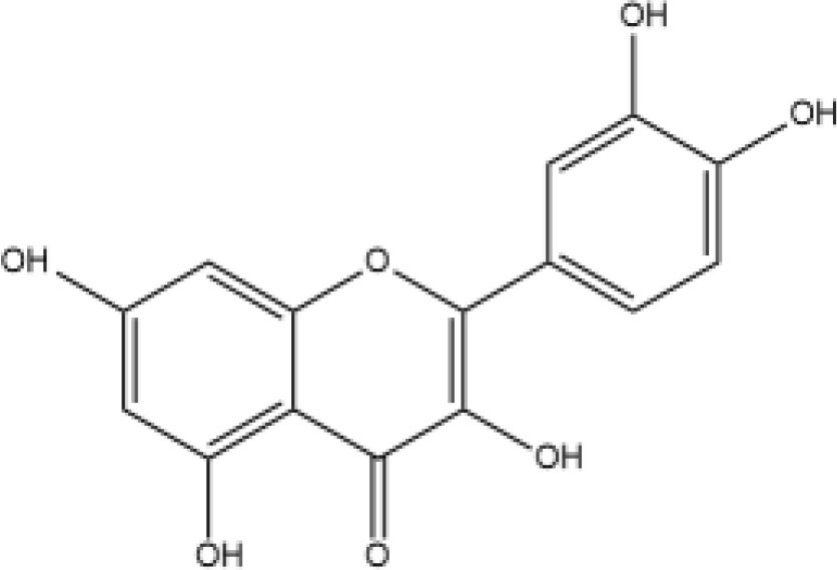	46.43	0.28	6
4	baicalein	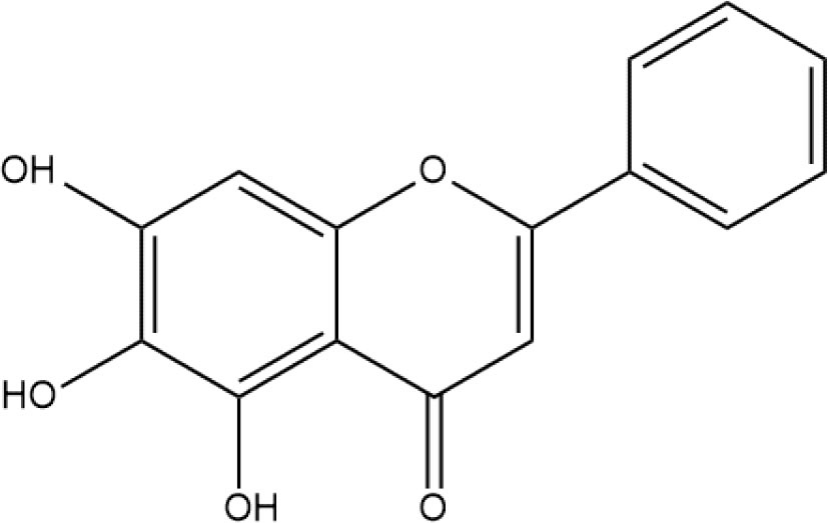	33.52	0.21	4
5	kaempferol	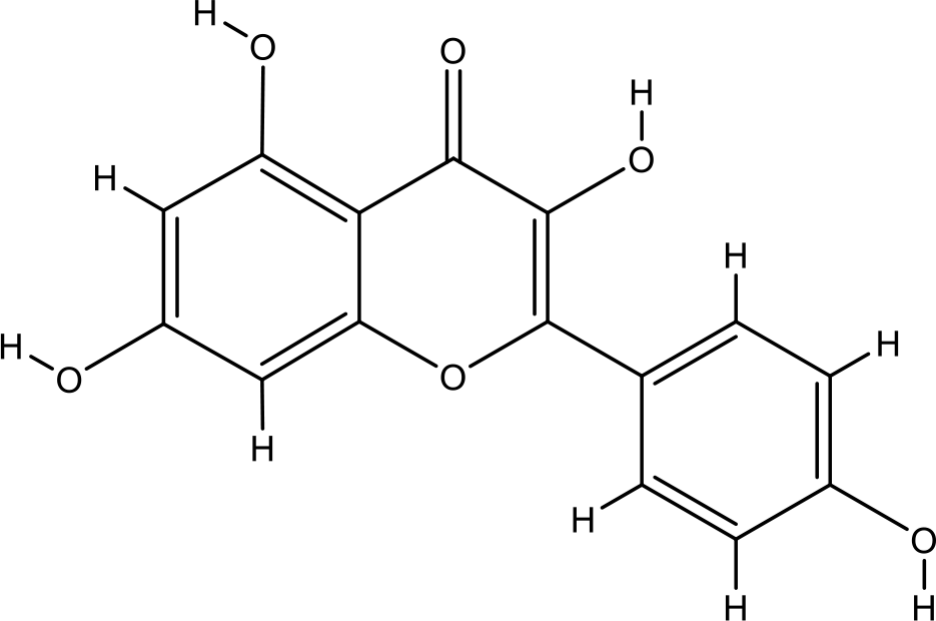	41.88	0.24	4
6	naringenin	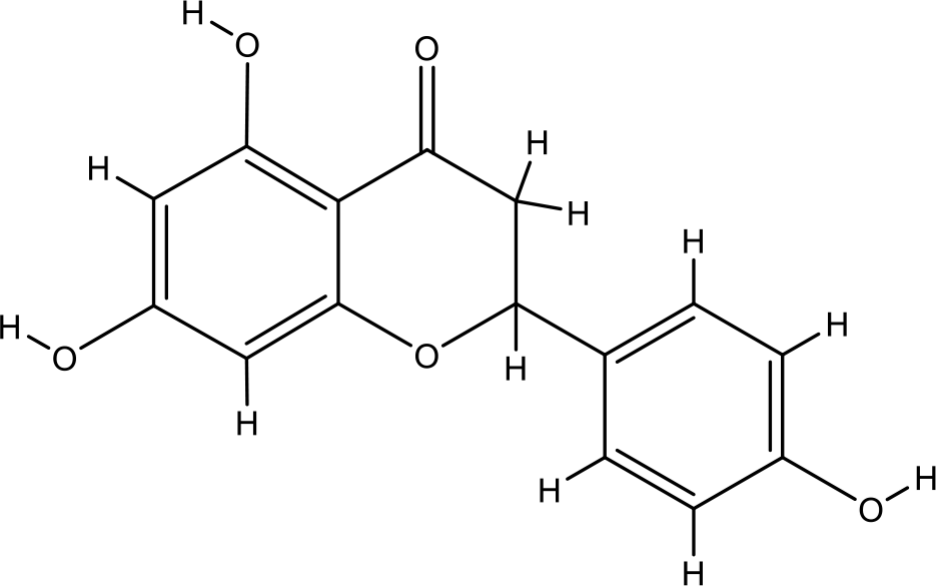	59.29	0.21	4
7	licochalcone A	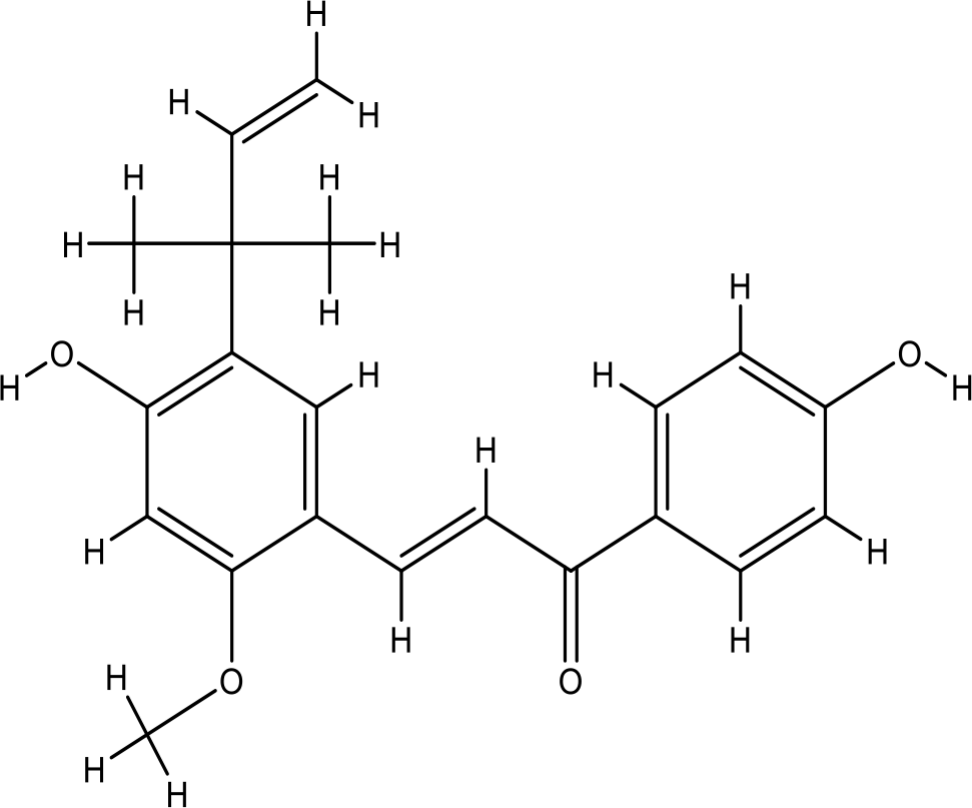	40.79	0.29	4
8	isorhamnetin	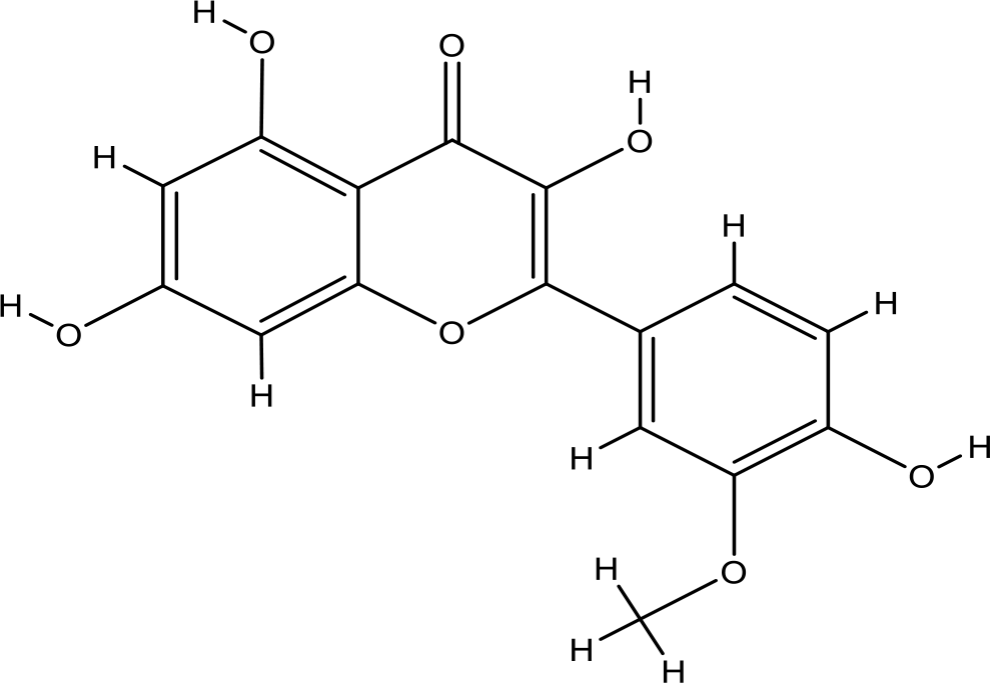	49.62	0.31	3
9	cryptotanshinone	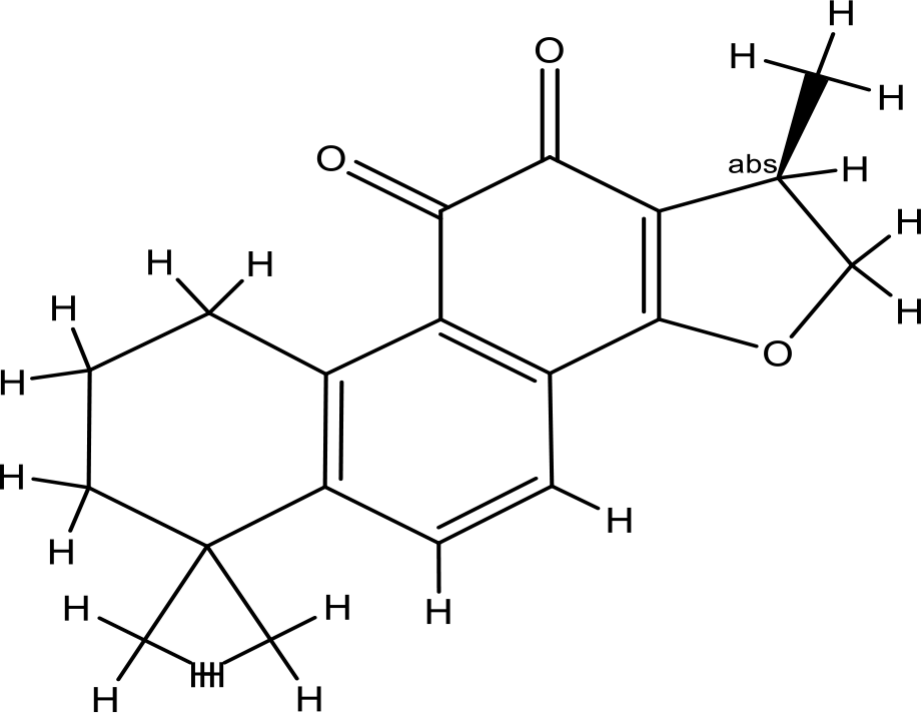	52.34	0.40	3
10	tanshinone iia	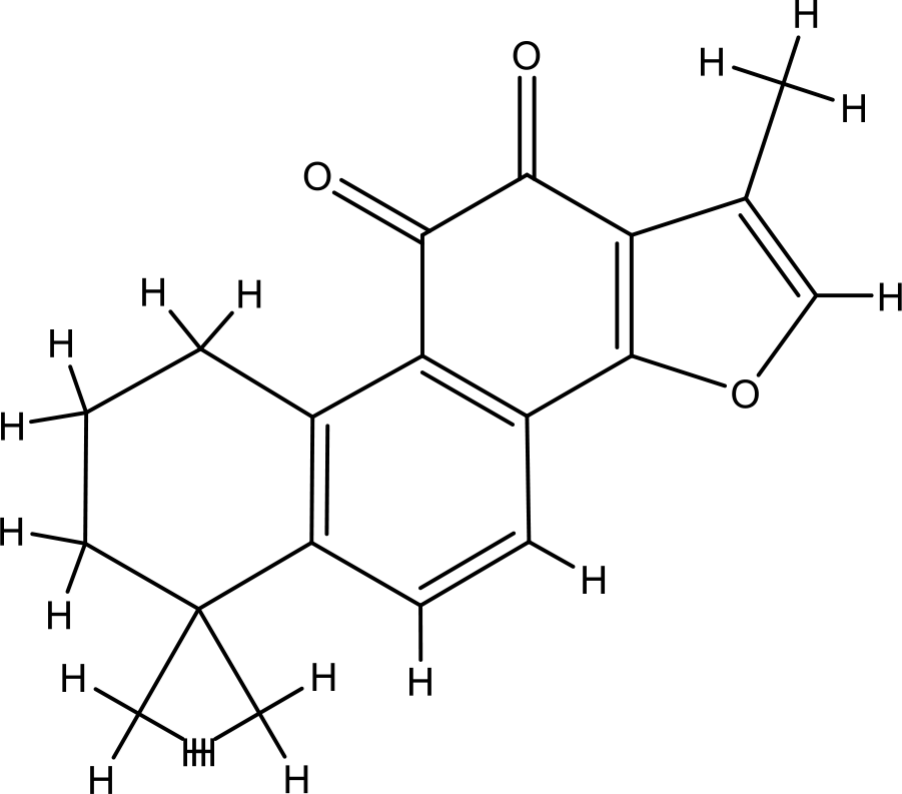	49.89	0.40	3

### 3.5 Molecular docking

**Figure 4:**
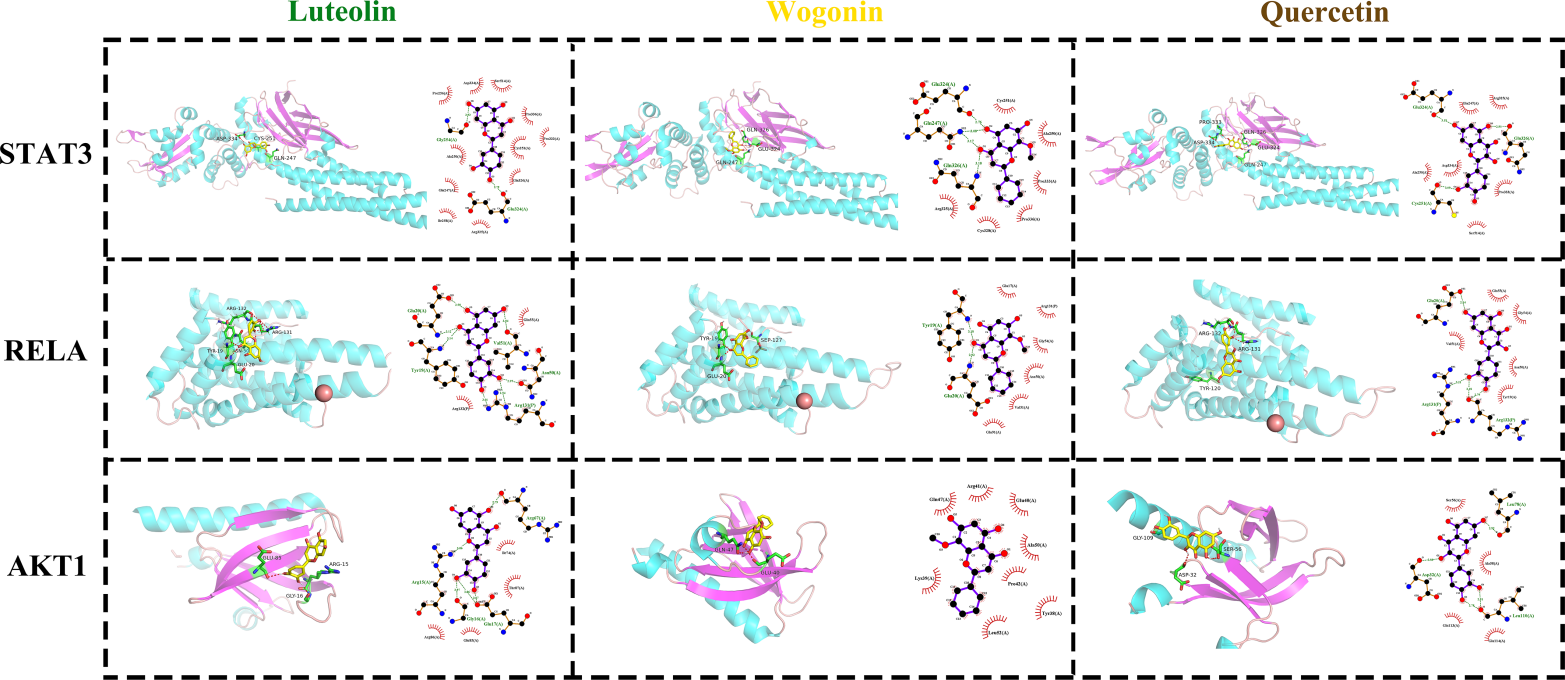
The docking modes of FHB top 3 active components (luteolin, wogonin, quercetin) and top 3 FHB-vitiligo targets (STAT3, RELA, AKT1). The 3D visualization of molecular docking is on the left side of each cell, and the 2D visualization is on the right side.

Based on the results of network pharmacology, molecular docking was performed to validate the binding modes of the top three active components (i.e., luteolin, quercetin, wogonin) and the top three FHB-vitiligo targets (i.e., RELA, STAT3, AKT1). **Table 3** displays the binding affinities of these components with target proteins. The 2D and 3D visualization of molecular docking of luteolin, quercetin, wogonin, and with RELA, STAT3, and AKT1 was presented in **Figure 4**.

**Table 3:** The binding energy of compound and core targets (kcal/mol).

Target	Target (PDB ID)	Compound	column amino acid bindings	Affinity (kcal/mol)
STAT3	4ZIA	luteolin	Gly254(A) Glu324(A) Pro256(A) Asp334(A) Ser514(A) Pro336(A) Pro333(A) Cys251(A) Gin326(A) Arg325(A) Ile258(A) Gin247(A) Ala250(A)	-7.4
		wogonin	Gln-247(A) Glu-324(A) Gln-326(A) Cys251(A) Ala250(A) Pro333(A) Pro336(A) Cys328(A) Arg325(A)	-7.3
		quercetin	Glu324(A) Gin326(A) Cys251(A) Gln247(A) Arg325(A) Pro333(A) Ser514(A) Ala250(A) Asp334(A)	-7.5
RELA	1VJ7	luteolin	Tyr-19(A) Arg131(P) Asn50(A) Val51(A) Glu20(A) Gin55(A) Arg132(P)	-7.0
		wogonin	Tyr19(A) Glu20(A) Glu17(A) Arg131(A) Gly54(A) Asn50(A) Val51(A) Glu91(A)	-6.7
		quercetin	Glu20(A) Arg131(P) Arg132(P) Gln55(A) Gly54(A) Asn50(A) Tyr19(A) Val51(A)	-6.9
AKT1	5AAR	luteolin	Arg15(A) Arg67(A) Glu17(A) Gly16(A) Ile74(A) Thr87(A) Glu8(A) Arg86(A)	-6.7
		wogonin	Gln47(A) Arg41(A) Glu40(A) Ala50(A) Pro42(A) Tyr38(A) Leu52(A) Lys39(A)	-6.3
		quercetin	Asp32(A) Leu78(A) Leu110(A) Ser56(A) Ala58(A) Glu114(A) Gln113(A)	-6.6

With -7.4, -7.3, and -7.5, respectively, luteolin, quercetin, and wogonin all had the strongest binding affinity to STAT3 protein (**Table 3**). In STAT3, luteolin created one hydrogen bond with Gly254(A) and one hydrogen bond with Glu324(A); wogonin made one hydrogen bond with Glu324(A), one with Gin326(A), and two with Gin247(A); quercetin made one hydrogen bond with Glu324, one with Gin326(A), and one with Cys251(A) (**Figure 4**).

### 3.6 Molecular dynamic simulation

**Figure 5:**
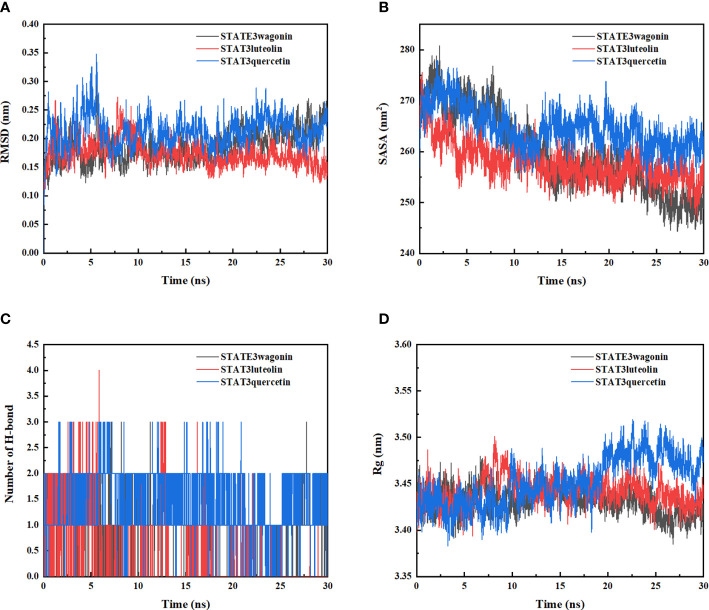
Molecular dynamics simulation of STAT3-wogonin, STAT3-luteolin, and STAT3-quercetin. **(A)** Root Mean Square Fluctuation (RMSD) values extracted from protein fit ligand of the protein–ligand docked complexes. **(B)** The compactness of protein structure in terms of the radius of gyration (Rg). **(C)** Solvent accessible surface area (SASA) analysis. **(D)** H bond formation between STAT3 and wogonin, luteolin, and quercetin.

The molecular dynamic simulation was performed to further confirm the binding abilities of STAT3 to luteolin, wogonin, and quercetin. RMSD curves indicated fluctuations in protein conformation. As shown in **Figure 5A**, STAT3-wogonin, STAT3-luteolin, and STAT3-quercetin were stable after 10 ns, and STAT3-luteolin was more stable within 30 ns, which meant the protein conformation of STAT3 does not change a lot after binding with three compounds, and the bindings are relatively stable. No break in the RMSD curve indicated that the compounds can be firmly attached to STAT3 without dissociating from the protein pocket during the simulation.

In addition, SASA analysis was performed to detect the degree of exposure of the receptor to surrounding solvent molecules during the simulation. The solvent-accessible surface area of STAT3-wogonin, STAT3-luteolin, and STAT3-quercetin complexes gradually decreased throughout the simulation, indicating that the bindings between them are gradually increased (**Figure 5B**).

The number of hydrogen bonds in the STAT3-compound complexes reflected their binding strengths. Among them, STAT3-quercetin had the highest hydrogen bond density and strength, followed by STAT3-wogonin, and STAT3-luteolin (**Figure 5C**). Binding free energy can be used to determine the variability and stability of ligand and protein binding patterns. In the MMPBSA calculations, the total binding free energies of STAT3-wogonin, STAT3-luteolin, and STAT3-quercetin were -70.039, -62.201, and -94.260 KJ/mol, respectively. Among them, STAT3-quercetin had the lowest binding free energy and the highest binding intensity, which was consistent with the results of molecular docking and dynamic simulation (**Table 4**). The radius-of-rotation curve represents the denseness of the overall protein structure. STAT3-wogonin, STAT3-luteolin, and STAT3-quercetin have stable radii of rotation, which was inhibited by the results of RMSD and indicated that the protein conformation is stable and the binding of three compounds does not affect the stability of STAT3 (**Figure 5D**).

**Table 4:** Analysis of protein ligand Molecular Mechanics Generalized Born Surface Area (MMPBSA) (KJ/mol).

Energy	STATE3-wogonin	STAT3-luteolin	STAT3-quercetin
Van der Waals Energy	-132.031	-171.889	-149.021
Electrostatic energy	-106.656	-121.359	-112.146
Polar solvation energy	137.971	189.885	137.958
Nonpolar solvation Energy	-44.111	-34.128	-45.896
Total Binding Energy	-144.827	-137.491	-169.105
TΔS	74.788	75.290	74.845
Total Binding Free Energy	-70.039	-62.201	-94.260

### 3.7 The treatment effect of FHB in vitiligo mice model

**Figure 6:**
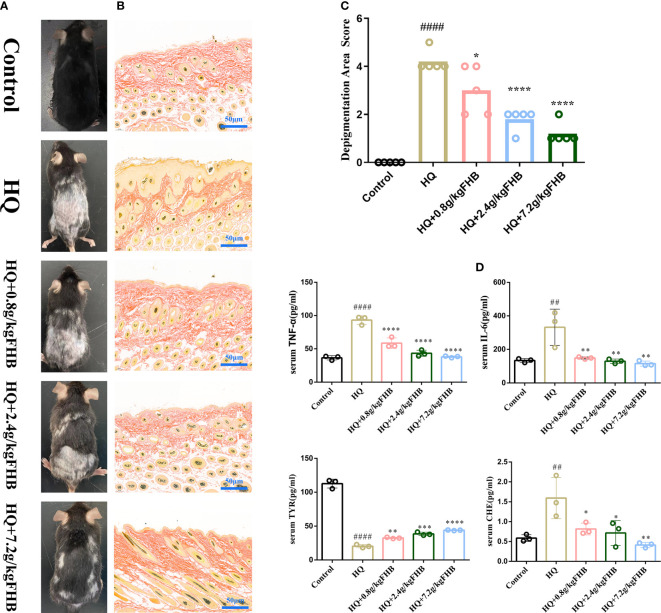
*In vivo* experimental verification of the anti-inflammatory and melanogenesis-promoting effects of FHB for vitiligo therapy. **(A)** Representative images of vitiligo areas on the back of mice of five groups (Control, Hydroquinone (HQ)), HQ+0.8g/kg FHB, HQ+2.4g/kg FHB, HQ+7.2g/kg FHB) on day 60. **(B)** Melanin staining image of the vitiligo area skin of each group (Control, HQ, HQ+0.8g/kg FHB, HQ+2.4g/kg FHB, HQ+7.2g/kg FHB) on day 60 (200 x). **(C)** Depigmentation area scores of five groups (Control, HQ, HQ+0.8g/kg FHB, HQ+2.4g/kg FHB, HQ+7.2g/kg FHB) (n=5). **(D)** The expressions of TNF-α, IL-6, TYR, and CHE in the blood of mice of five groups (Control, HQ, HQ+0.8g/kg FHB, HQ+2.4g/kg FHB, HQ+7.2g/kg FHB) determined by ELISA (n=3). The significance of differences was determined using the t-test. “#” represents a significant difference between the labeled group and the control group, and “*” represents a significant difference between the labeled group and the HQ group. *P < 0.05, ## or **P<0.01, ***P<0.001, #### or ****P<0.0001.

In our research, 5 mg 3% hydroquinone (HQ) ointment was applied to the shaved area on the back of the mice to induce the vitiligo mice model. As seen in **Figures 6A, B**, the HQ-induced model mice displayed evident depigmentation of the skin on the back, compared to the mice of the control group. FHB therapy groups significantly reduced the depigmentation area in vitiligo model mice in a dose-dependent manner (**Figure 6C**).

The results of Masson-Fontana staining showed that after 60 days’ treatment, the epidermis of mice in the control group had a large number of melanin granules in hair follicle cells with no hyperplasia, whereas mice in the vitiligo model group had significantly fewer melanin granules, and the epidermis of vitiligo mice was hyperplastic and the number of hair follicles was reduced (**Figure 6B**). The epidermis of vitiligo mice treated with low (0.8g/kg), medium (2.4g/kg), and high (7.2g/kg) doses of FHB also showed different degrees of hyperplasia, but the number of hair follicles and melanin granules increased significantly as compared to the mice of the vitiligo model group.

### 3.8 FHB reduces the level of inflammation and promotes melanin production

**Figure 7:**
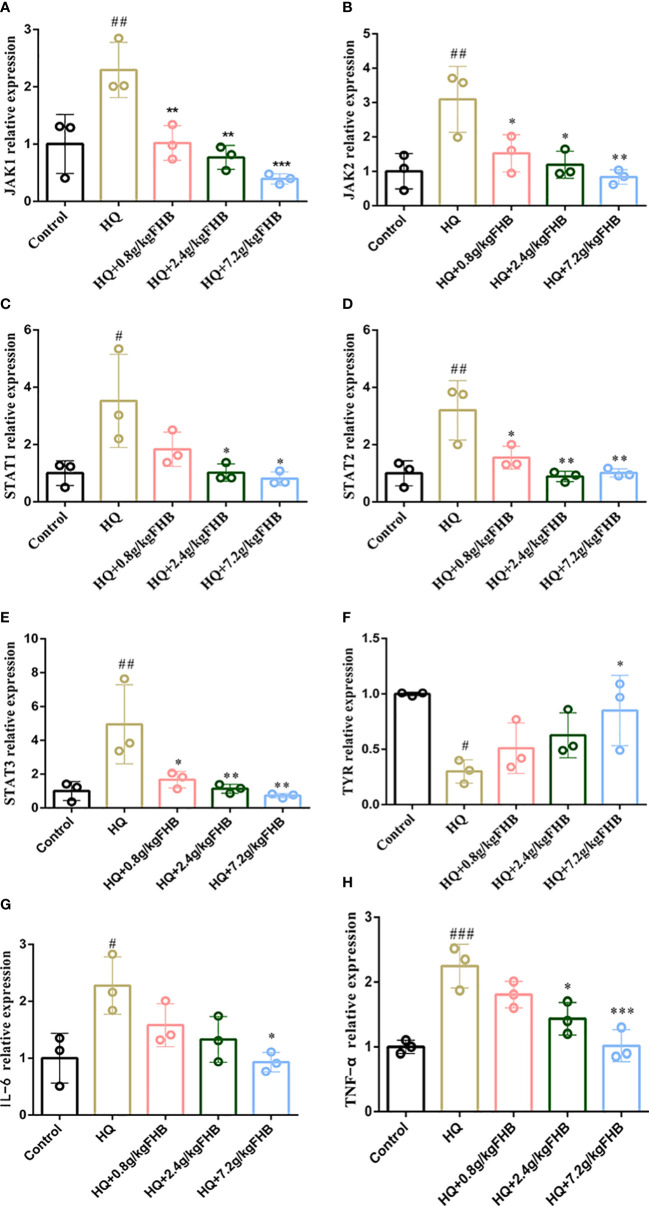
The mRNA relative expression of **(A)** Jak1, **(B)** Jak2, **(C)** Stat1, **(D)** Stat2, **(E)** Stat3, **(F)** TYR, **(G)** (IL-6) and **(H)** TNF-α in vitiligo area skins. The significance of differences was determined using the t-test. “#” represents a significant difference between the labeled group and the control group, and “*” represents a significant difference between the labeled group and the HQ group. # or *P < 0.05, ## or **P<0.01, ### or ***P<0.001.

Cytokines (e.g., TNF-α and IL-6) -mediated inflammation is important in the development of vitiligo, and CHE and TYR are molecules related to melanin synthesis. As shown in **Figure 6D**, the FHB therapy group significantly reduced the levels of inflammatory factors TNF-α and IL-6 in the blood of vitiligo mice, which was consistent with the results anticipated by network pharmacology. Furthermore, in the FHB therapy group, the levels of CHE in the peripheral blood of vitiligo mice were much lower, but the levels of TYR were significantly greater than in the model group. The mRNA expression of TYR of FHB in the therapy group also increased in a dose-dependent manner (**Figure 7F**).

**Figure 8:**
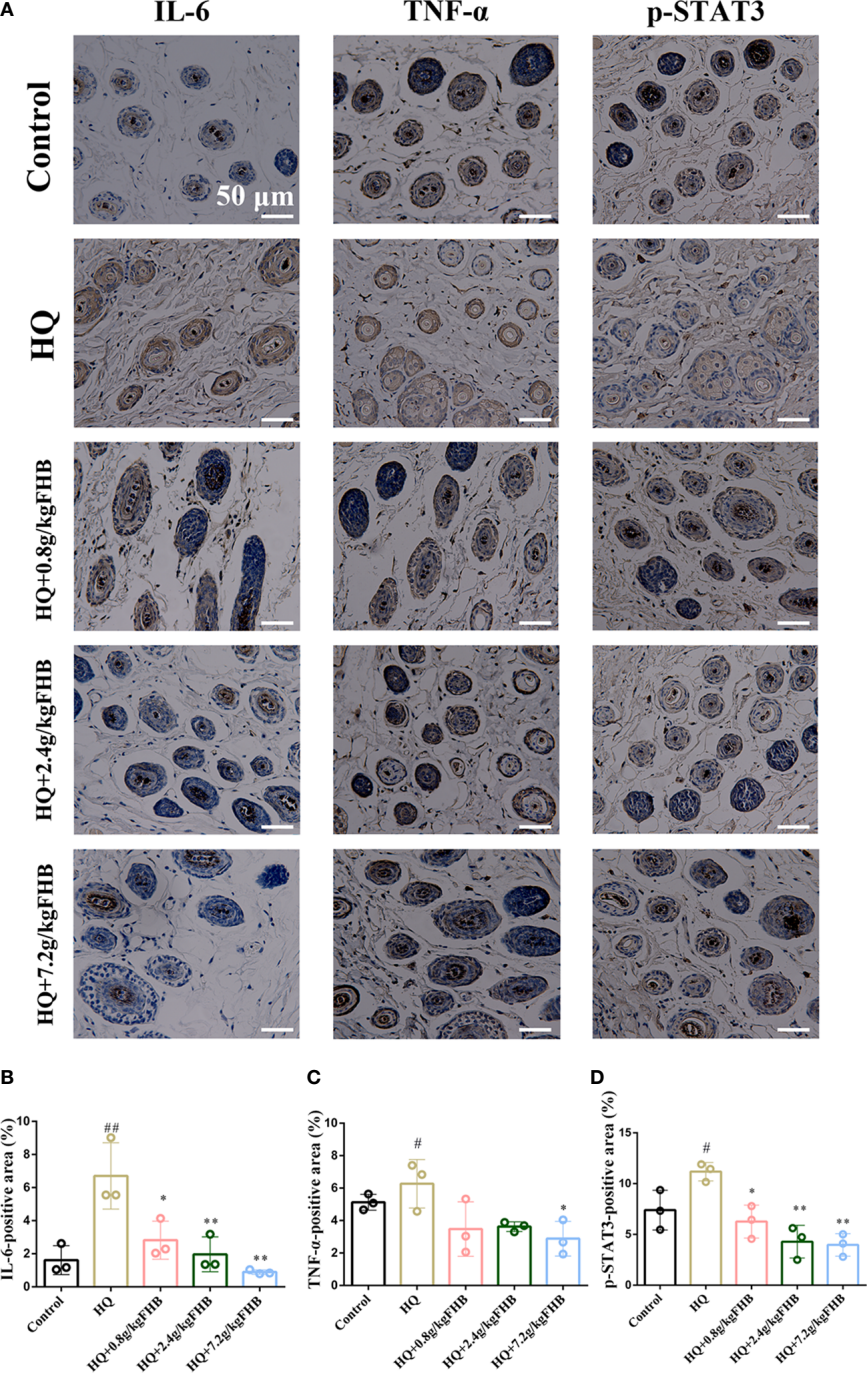
IHC staining of IL-6, TNF-α, p-STAT3 in vitiligo area skins. (200 x) **(A)** Representative IL-6, TNF-α, p-STAT3 staining images of five groups (Control, Hydroquinone (HQ), HQ+0.8g/kg FHB, HQ+2.4g/kg FHB, HQ+7.2g/kg FHB). Bar = 50 μm. **(B)** IL-6 positive areas of five groups are evaluated based on staining images from independent experiments (n=3). **(C)** TNF-α positive areas of five groups are evaluated based on staining images from independent experiments (n=3). **(D)** p-STAT3 positive areas of five groups are evaluated based on staining images from independent experiments (n=3). The significance of differences was determined using the t-test. “#” represents a significant difference between the labeled group and the control group, and “*” represents a significant difference between the labeled group and the HQ group. # or *P < 0.05, ## or **P<0.01.

In addition, we used IHC to further investigate the expression of inflammatory markers IL-6 and TNF-α in the skin tissues. In comparison to the control group, HQ-induced vitiligo mice had a significant increase of IL-6 and TNF-α in both skin tissues and melanocytes, but FHB therapy can effectively decrease them (**Figure 8A**). The IHC-positive areas of IL-6 and TNF-α of the FHB high-dose treatment group (HQ+7.2g/kg FHB) were even lower than those of the control group, indicating that FHB strongly inhibited inflammation in the topical vitiligo skin (**Figures 8B, C**). These findings demonstrate that FHB has a molecular mechanism for reducing inflammation and promoting melanogenesis, with the high-dose FHB therapy group (HQ+7.2g/kg FHB) having the most significant effect.

To further investigate the anti-inflammatory ability of FHB on vitiligo mice at the transcriptional level, we used PCR to detect the expression of IL-6 and TNF-α in the lesioned skin. As shown in **Figures 7G, H**, This showed significant mRNA expression of IL-6 and TNF-α in FHB.

### 3.9 Inhibition effects of FHB on targets related to the JAK-STAT signaling pathway

According to the findings of network pharmacology and molecular docking, FHB may treat vitiligo by binding to STAT3 and decreasing its phosphorylation, hence inhibiting the transactivation of the JAK-STAT signaling pathway. To verify the effect of FHB on the JAK-STAT signaling pathway in vitiligo mice *in vivo*, we first investigated the expression of p-STAT3, one of the markers of JAK-STAT signaling pathway activation, in vitiligo skin tissues by IHC. FHB suppressed the expression of p-STAT3 in vitiligo mice in a dose-dependent manner, implying that FHB is involved in JAK-STAT pathway regulation (**Figures 8A, D**).

To further investigate the effect of FHB on the JAK-STAT pathway in vitiligo mice at the transcriptional level, we used qRT-PCR to examine the influence of FHB on the mRNA expression of Jak1, Jak2, Stat1, Stat2, and Stat3 in the lesion region. The mRNA expression of Jak1, Jak2, Stat1, Stat2, and Stat3 was dramatically elevated in the HQ-induced vitiligo model mice, as shown in **Figure 7**. However, FHB therapy significantly hampered the increase of mRNA expression of Jak1, Jak2, Stat1, Stat2, and Stat3 in vitiligo mice, and this inhibitory effect was dose-dependent (**Figure 7**). indicating that FHB influences vitiligo development by indirectly or directly mediating the transcription of upstream and downstream genes in the JAK-STAT pathway.

**Figure 9:**
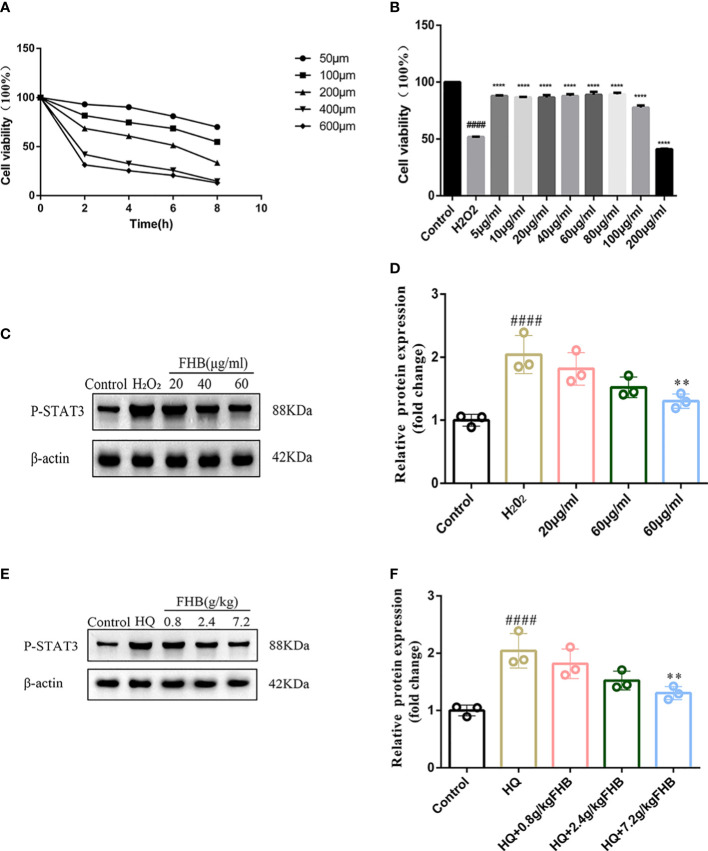
FHB inhibits STAT3 phosphorylation from the protein level. **(A)** Effect of concentration and time of H_2_0_2_ action on the survival rate of B16 cells. **(B)** Effect of different concentrations of FHB on the survival rate of B16 cells. **(C)** The protein expression levels of P-STAT3 in B16 cells after treatment with different concentrations (20, 40, 60μg/ml) of FHB for 24h. (n=3) **(D)**The grey value statistics of the protein expression levels of P-STAT3 in B16 cells after treatment with different concentrations (20, 40, 60μg/ml) of FHB for 24h. (n=3) **(E)** The protein expression levels of P-STAT3 in tissue after treatment with different concentrations (0.8, 2.4, 7.2g/kg) of FHB. **(F)** The grey value statistics of the protein expression levels of P-STAT3 in tissue after treatment with different concentrations (0.8, 2.4, 7.2g/kg) of FHB. The significance of differences was determined using the t-test. “#” represents a significant difference between the labeled group and the control group, and “*” represents a significant difference between the labeled group and the model group. **P<0.01, #### or ****P<0.0001.

In addition, we used WB assay to detect the protein expression level of P-STAT3 in lesion skin and B16 vitiligo model cells by FHB. To establish vitiligo model cells, we used 200μmol H_2_O_2_ to induce B16 cells for 6 hours to achieve an optimal state of oxidative stress (**Figure 9A**). Then we used different concentrations of FHB to treat B16 vitiligo model cells, and 20μg/ml, 40μg/ml, and 60μg/ml were selected as the dosing concentrations in the absence of cellular toxicity (**Figure 9B**). It can be concluded from the cellular level that a high dose of FHB can significantly inhibit the expression of P-STAT3 in vitiligo model cells (**Figures 9C, D**). From the WB results of lesioned skin, the expression of P-STAT3 in the lesioned area was significantly reduced in the FHB-treated group (**Figures 9E, F**), which was consistent with the previous PCR results, FHB can inhibit the development of vitiligo by suppressing transcription factors upstream and downstream of the JAK-STAT pathway.

### 3.10 *In vivo* biosafety evaluation of the FHB

**Figure 10:**
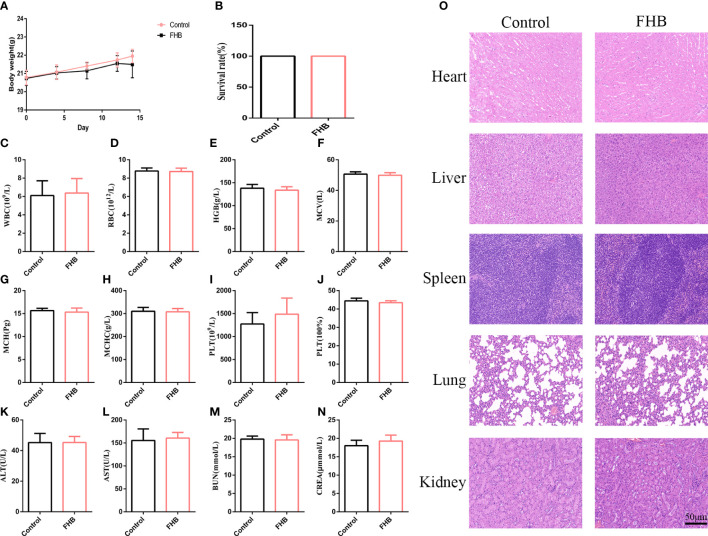
*In vivo* biosafety of the FHB. **(A)** The body-weight curve in 14 days of the mice intravenously injected with FHB (0.75g/ml) (n = 10). **(B)** The survival rate of the drug-administered mice at day 14. **(C–J)** Hematology surveys of the mice show all parameters in a normal range, and there are no significant differences between the control groups and FHB groups (n = 5). **(K-N)** The blood biochemical test shows the normal function of livers (ALT and AST) and kidneys (BUN and CREA) (n = 5). **(O)** HE staining of the main organs. Bar = 50 μm. (200x). The significance of differences was determined using the t-test.

After 14 days of gavage administration, the survival rate of the two groups of rats was 100% and there was no significant difference in body weight change (**Figures 10A, B**). Hematological parameters such as white blood cells (WBC), red blood cells (RBC), hemoglobin (HGB), mean red blood cell volume (MCV), mean red blood cell hemoglobin content (MCH), mean red blood cell hemoglobin containing concentration (MCHC), platelets (PLT), and hematocrit (HCT) were within the normal range (**Figures 10C–J**). The blood biochemical parameters such as the liver function indexes aspartate aminotransferase (AST) and alanine aminotransferase (ALT), and the kidney function indexes blood urea nitrogen (BUN) and creatinine (CREA) were at the normal level (**Figures 10K–N**). These results suggest that FHB has no hepatic or renal toxicity. From the results of HE, no significant lesions and inflammation were observed in the viscera of each group, indicating that FHB did not show organ toxicity within 14 days (**Figure 10O**).

## 4 Discussion

Vitiligo is a common pigmentary disease with serious psychosocial impacts. Genetic factors, immune dysregulation, oxidative stress, and melanocyte adhesion defects are among the mechanisms that cause melanocyte loss, and a combination of these processes may be responsible for depigmentation lesions, with immune dysregulation playing a key role in the pathogenesis of vitiligo (6). The present conventional Western medical therapy for vitiligo fails to produce the desired therapeutic effect in some individuals and is associated with side effects and recurrence rates (4). The therapeutic application of alternative therapies such as traditional Chinese medicine (TCM) is receiving widespread attention. Fufang Honghua Buji (FHB) granules, a homemade formula from Shanghai Skin Disease Hospital, have been demonstrated to be therapeutically useful in vitiligo (26). However, the compound formula of TCM has diverse pharmacological effects involving various components, targets, and pathways, making it difficult to identify FHB’s composition and interactions between specific chemical components, as well as to illustrate the precise molecular mechanisms for therapy.

Network pharmacology is consistent with TCM theory’s holistic view, which provides a systematic perspective and research pattern for predicting bioactive chemicals, probable target genes, and disease-related pathways in TCM formulas by building a “drug-target-disease” network. In this study, we employed network pharmacology in combination with molecular docking, Molecular dynamics simulation, and experimental validation to explore the bioactive components and molecular mechanisms of FHB for vitiligo. Our findings revealed that the majority of the active components of FHB work synergistically to treat vitiligo. Besides, numerous FHB chemicals affect multiple targets and have overlapping targets. Luteolin, wogonin, and quercetin may be the most active FHB chemicals in vitiligo treatment since they affect most of the disease-related targets. They are all-natural flavonoids having antioxidant, anti-inflammatory, and anti-cancer properties. Luteolin has been demonstrated to inhibit the production of IL-8 (CXCL8), a crucial inflammatory chemokine in vitiligo, as well as to stimulate melanin synthesis in cell and zebrafish models (27, 28). Wogonin can act directly on immune cells and inhibit the production of inflammatory factors such as IL-6, TNF-α, and reactive oxygen species. Downregulation of toll-like receptors, activation of the Nrf2 and PPAR signaling pathways, and suppression of inflammation-related pathways such as MAPK, PI3K-Akt, NF-B, and JAK-STAT are among the molecular mechanisms participating (29–32). Quercetin is one of the most prevalent flavonoids in the human diet, which can protect melanocytes from H_2_O_2_-induced oxidative damage (33). As an anti-inflammatory agent, quercetin also can suppress inflammatory pathways such as JAK-STAT, PI3K/Akt, and HIF-1, all of which are involved in solid tumors and neurological disorders (34–36). These previous studies and our research provide evidence that luteolin, wogonin, and quercetin may have the ability to treat vitiligo-related targets. However, FHB exerts a therapeutic effect on vitiligo through synergistic interactions between components, so whether the usage of reasonable combination doses of luteolin, wogonin, and quercetin has similar therapeutic effects with FHB remains to be confirmed.

In addition, network pharmacology analysis identified AKT1, RELA, and STAT3 as key targets of FHB for vitiligo treatment. Molecular docking results further confirmed that the major components in FHB bind to these key protein targets, with luteolin, wogonin, and quercetin having the highest binding energy to STAT3, implying that FHB may suppress the JAK-STAT pathway by binding to STAT3 to inhibit STAT3’s expression or/and phosphorylation, hence curing vitiligo. Based on the understanding of the key role of specific targets in the pathogenesis of vitiligo, targeted therapeutic agents such as JAK, PDE-4 inhibitors, and anti-CD122 blocking antibodies have been developed to treat vitiligo disease (7). However, several of these have been demonstrated to be ineffective in vitiligo patients (37, 38). Although JAK inhibitors prove successful in some case reports and open-label trials, they may be paradoxical: one patient with rheumatoid arthritis acquired new-onset vitiligo after systemic tofacitinib treatment (39); besides, JAK inhibitors are incapable of depleting skin-localized tissue-resident memory T (T_RM_) cells, which may make repigmentation not durable (40). FHB, unlike targeted agents, has multiple components, and network pharmacology results show that FHB inhibits multiple pro-inflammatory cytokines and disease targets, suggesting that it may be a supplemental option for the treatment of severe pigment loss and persistent vitiligo recurrence. Furthermore, GO and KEGG pathway analysis revealed that FHB may engage HIF-1, NF-kappaB, JAK-STAT, and other pathways to suppress the development of vitiligo, and has effects on “cytokine receptor binding”, “cytokine activity”, “nuclear receptor activity” and so on, which is closely related to the reported pathogenesis of vitiligo (41–44).

As mentioned previously, the results of network pharmacology and molecular docking imply that the STAT3 and the JAK-STAT pathway may be the potential therapeutic targets of FHB in vitiligo. *In vivo* experiments further demonstrate that FHB is closely related to the JAK-STAT signaling pathway, with STAT3, a downstream transcription factor of the JAK-STAT signaling pathway, being a key target protein. The JAK-STAT signaling pathway is a typical pathway involved in inflammatory and autoimmune illnesses, as many type I/II cytokines (e.g., IL-2, IL-15, IL-6, IFN-γ) can bind to specific JAKs(Janus kinases) and activate downstream STAT (signal transducer and activator of transcription) proteins’ phosphorylation and dimerization (45, 46). Although the exact mechanism of vitiligo onset and melanocyte loss is unknown, multiple investigations have shown that CD8^+^ T-cell infiltration near melanocytes is involved in the elimination and death of pigment cells (47, 48). Activated CXCR3^+^ CD8^+^ T cells stimulate keratin-forming cells to release IFN-γ and chemokines that increase melanocyte detachment and death via the JAK/STAT pathway, resulting in continued recruitment of CXCR3^+^ CD8^+^ T cells and the establishment of a positive feedback loop (49, 50). Our findings found that FHB not only inhibited the expression of STAT3 and p-STAT3 in the JAK-STAT signaling pathway in hydroquinone (HQ)-induced vitiligo mice but also suppressed the expression of genes of JAK-STAT pathway (including JAK1, JAK2, STAT1, STAT2), as well as the expression of inflammatory cytokines TNF-α and IL-6, which is consistent with the results predicted by network pharmacology. On the one hand, there is considerable homology between STAT1 and STAT3, and off-target STAT1 blockade by FHB may be responsible for the reduced STAT1 expression (51). On the other hand, a more likely explanation is that FHB extensively inhibits the expression of JAK-STAT upstream type I/II inflammatory cytokines in vitiligo mice, which in turn inhibits the activation and expression of downstream factors such as JAKs and STATs, further suppressing inflammatory factor expression and forming a positive feedback loop of immune regulation.

Furthermore, the observational results showed that FHB improved melanin deposition in vitiligo mice. From a molecular perspective and tyrosinase (TYR) tend to be reduced in expression in vitiligo models and clinical, and the expression of cholinesterase (CHE) may increase (52, 53). Skin inflammation in vitiligo also affects the synthesis of TYR (54). The vitiligo inducer HQ used in our study can inhibit the expression of TYR proteins and increase the activity of CHE in serum, thus preventing the catalytic conversion of TYR to melanin (55). In our study, the expression of melanogenesis-related genes TYR in the skin tissues of FHB-administered vitiligo mice was significantly increased, as were the concentrations of CHE and TYR in the serum, and the expression of inflammatory factors TNF-α and IL-6 were reduced, confirming the anti-inflammatory and melanogenesis-promoting effects of FHB.

This study still has several limitations. First, some chemical components and targets have not yet been reported in the network pharmacology database, while vitiligo-related targets are continually being updated, so this study may not be completely accurate. Second, we chose representative STAT3 target genes and relevant JAK-STAT pathways for pharmacological experimental validation, but we did not test the inhibitory effects of FHB on MAPK, PI3K/Akt, NF-κB, HIF-1α, and other inflammatory pathways associated with vitiligo and predicted network pharmacological results. Furthermore, though we identified luteolin, wogonin, and quercetin as probable key chemicals in FHB, they may not be equivalent to FHB, and the synergistic effects of the three key compounds still require experimental validation.

## 5 Conclusion

In this study, we discovered that FHB had therapeutic effects on vitiligo by modulating various components, targets, and pathways. Luteolin, wogonin, and quercetin are key active components of FHB, which may act on targets like RELA, AKT1, STAT3, MAPK14, TNF-α, and IL-6 to regulate pathways including NF-κB, PI3K/Akt, and JAK-STAT signaling pathways, thereby exerting therapeutic effects. The results of molecular docking and Molecular dynamics simulation demonstrated that the main components and key target proteins predicted by network pharmacology bind well. The *in vivo* experiments further revealed that FHB could inhibit STAT3’s expression, phosphorylation, and dimerization to exert an anti-inflammatory and melanogenesis-promoting effect, and treat vitiligo by downregulating the JAK-STAT signaling pathway. Our study first combined network pharmacology, molecular docking, Molecular dynamics simulation, and experimental verification to demonstrate that FHB could significantly reduce the expression of JAK-STAT pathway genes, alleviate inflammation and enhance the secretion of melanogenesis-related factors TYR to promote melanin pigmentation, which may support FHB as an alternative therapy for the treatment of inflammation and vitiligo.

## Data availability statement

The original contributions presented in the study are included in the article/**Supplementary Material**. Further inquiries can be directed to the corresponding author/s.

## Ethics statement

The animal study was reviewed and approved by Ethics Committee of Shanghai Skin Disease Hospital.

## Author contributions

XL, FM, and RX conceived and designed the article; JY and HH performed experimental work. ZT, HP, ZC, and QZ reviewed and edited the manuscript. All authors contributed to the article and approved the submitted version.
