# Supplementary material for: Combining network pharmacology, molecular docking, molecular dynamics simulation, and experimental verification to examine the efficacy and immunoregulation mechanism of FHB granules on vitiligo
Source: Front Immunol. 2023 Jul 27;14:1194823. doi: 10.3389/fimmu.2023.1194823 (PMC10414113; doi:10.3389/fimmu.2023.1194823)
Supplement: Supplementary file 1 [file Table_1.docx]

Supplementary Material

**Combining network pharmacology, molecular docking, Molecular dynamics simulation, and experimental verification to examine the efficacy and immunoregulation mechanism of FHB granules on vitiligo**

**Xiaolong Li^1,2^**^†^**; Fengze Miao^1,2^**^†^**; Rujuan Xi^1,2^**^†^**; Zongguang Tai^1,2^;Huijun Pan^1,2^;Hao Huang^1,2^; Junxia Yu^1,2^; Zhongjian Chen^1,2*^; Quangang Zhu^1,2*^**

^1^Shanghai Skin Disease Hospital, Tongji University School of Medicine, Shanghai 200443, China

^2^Shanghai Engineering Research Center for Topical Chinese Medicine, Shanghai 200443, China

***Correspondence:**

Zhongjian Chen^*^

aajian818@163.com

Quangang Zhu^*^

[qgzhu@126.com](mailto:qgzhu@126.com)

1. **Supplementary Table 1. 339 active components of FHB from TCMSP and HERB.**

| Ingredient name | OB | DL | Source |
| --- | --- | --- | --- |
| Stigmasterol | 43.82985 | 0.75665 | TCMSP、HERB |
| Baicalin | 40.12361 | 0.75264 | TCMSP、HERB |
| beta-sitosterol | 36.91391 | 0.75123 | TCMSP、HERB |
| poriferast-5-en-3beta-ol | 36.91391 | 0.75034 | TCMSP、HERB |
| CLR | 37.8739 | 0.67677 | TCMSP、HERB |
| lignan | 43.31816 | 0.65067 | TCMSP、HERB |
| beta-carotene | 37.18433 | 0.58358 | TCMSP、HERB |
| Flavoxanthin | 60.41294 | 0.55609 | TCMSP、HERB |
| Phytoene | 39.56307 | 0.50463 | TCMSP、HERB |
| phytofluene | 43.18173 | 0.50316 | TCMSP、HERB |
| 4-[(E)-4-(3,5-dimethoxy-4-oxo-1-cyclohexa-2,5-dienylidene)but-2-enylidene]-2,6-dimethoxycyclohexa-2,5-dien-1-one | 48.46631 | 0.36494 | TCMSP、HERB |
| Pyrethrin II | 48.35707 | 0.35025 | TCMSP、HERB |
| lupeol-palmitate | 33.98365 | 0.31954 | TCMSP、HERB |
| quercetagetin | 45.00699 | 0.30991 | TCMSP、HERB |
| quercetin | 46.43335 | 0.27525 | TCMSP、HERB |
| 6-Hydroxykaempferol | 62.13267 | 0.27266 | TCMSP、HERB |
| luteolin | 36.16263 | 0.24552 | TCMSP、HERB |
| 6-Hydroxynaringenin | 33.22921 | 0.24203 | TCMSP、HERB |
| kaempferol | 41.88225 | 0.24066 | TCMSP、HERB |
| baicalein | 33.51892 | 0.20888 | TCMSP、HERB |
| qt_carthamone | 51.02582 | 0.20055 | TCMSP、HERB |
| 7,8-dimethyl-1H-pyrimido[5,6-g]quinoxaline-2,4-dione | 45.75094 | 0.18605 | TCMSP、HERB |
| 20-Hexadecanoylingenol | 32.7 | 0.65 | HERB |
| Onjixanthone I | 79.16 | 0.3 | HERB |
| Linoleyl acetate | 42.1 | 0.2 | HERB |
| carthamidin | 41.15 | 0.24 | HERB |
| alpha-onocerin | 39.31 | 0.73 | HERB |
| Stigmasterol | 43.82985 | 0.75665 | TCMSP、HERB |
| beta-sitosterol | 36.91391 | 0.75123 | TCMSP、HERB |
| hederagenin | 36.91391 | 0.75072 | TCMSP、HERB |
| Coronaridine | 34.96552 | 0.67559 | TCMSP、HERB |
| Siegesesteric acid II | 51.97925 | 0.48412 | TCMSP、HERB |
| Siegesmethyletheric acid | 60.71556 | 0.43321 | TCMSP、HERB |
| 15alpha-Hydroxy-ent-kaur-16-en-19-oic acid | 58.7273 | 0.38365 | TCMSP、HERB |
| (1R)-1-[(2S,4aR,4bS,7R,8aS)-7-hydroxy-2,4b,8,8-tetramethyl-4,4a,5,6,7,8a,9,10-octahydro-3H-phenanthren-2-yl]ethane-1,2-diol | 46.70141 | 0.31312 | TCMSP、HERB |
| Vernolic acid | 37.6253 | 0.18715 | TCMSP、HERB |
| Stigmasterol | 43.82985 | 0.75665 | TCMSP、HERB |
| beta-sitosterol | 36.91391 | 0.75123 | TCMSP、HERB |
| campest-5-en-3beta-ol | 37.57682 | 0.71481 | TCMSP、HERB |
| CLR | 37.8739 | 0.67677 | TCMSP、HERB |
| quercetin | 46.43335 | 0.27525 | TCMSP、HERB |
| kaempferol | 41.88225 | 0.24066 | TCMSP、HERB |
| Methyl arachidonate | 46.89969 | 0.23381 | TCMSP、HERB |
| (2R)-5,7-dihydroxy-2-(4-hydroxyphenyl)chroman-4-one | 42.36332 | 0.21141 | TCMSP、HERB |
| Sitosterol alpha1 | 43.28127 | 0.78354 | TCMSP、HERB |
| beta-sitosterol | 36.91391 | 0.75123 | TCMSP、HERB |
| hederagenin | 36.91391 | 0.75072 | TCMSP、HERB |
| campesterol | 37.57682 | 0.71476 | TCMSP、HERB |
| GA87 | 68.85255 | 0.57188 | TCMSP、HERB |
| Gibberellin A44 | 101.6132 | 0.54105 | TCMSP、HERB |
| GA30 | 61.71774 | 0.54002 | TCMSP、HERB |
| GA63 | 65.54356 | 0.53773 | TCMSP、HERB |
| GA122-isolactone | 88.11097 | 0.5371 | TCMSP、HERB |
| GA121-isolactone | 72.69926 | 0.5371 | TCMSP、HERB |
| GA54 | 64.20665 | 0.5349 | TCMSP、HERB |
| 2,3-didehydro GA77 | 88.08055 | 0.53017 | TCMSP、HERB |
| GA60 | 93.16869 | 0.53004 | TCMSP、HERB |
| GA77 | 87.89416 | 0.52764 | TCMSP、HERB |
| 2,3-didehydro GA70 | 63.29363 | 0.49632 | TCMSP、HERB |
| GA122 | 64.79329 | 0.49617 | TCMSP、HERB |
| gibberellin 7 | 73.80062 | 0.49609 | TCMSP、HERB |
| gibberellin 17 | 94.64115 | 0.49443 | TCMSP、HERB |
| GA119 | 76.36423 | 0.49382 | TCMSP、HERB |
| 4a-formyl-7alpha-hydroxy-1-methyl-8-methylidene-4aalpha,4bbeta-gibbane-1alpha,10beta-dicarboxylic acid | 88.59516 | 0.46382 | TCMSP、HERB |
| GA120 | 84.84964 | 0.45279 | TCMSP、HERB |
| 3-O-p-coumaroylquinic acid | 37.6279 | 0.28636 | TCMSP、HERB |
| Populoside_qt | 108.8855 | 0.20476 | TCMSP、HERB |
| Stigmasterol | 43.82985 | 0.75665 | TCMSP、HERB |
| beta-sitosterol | 36.91391 | 0.75123 | TCMSP、HERB |
| sitosterol | 36.91391 | 0.7512 | TCMSP、HERB |
| campest-5-en-3beta-ol | 37.57682 | 0.71481 | TCMSP、HERB |
| Schkuhrin I | 54.45027 | 0.52014 | TCMSP、HERB |
| Supraene | 33.54594 | 0.42161 | TCMSP、HERB |
| Schizonepetoside B | 31.02273 | 0.28216 | TCMSP、HERB |
| quercetin | 46.43335 | 0.27525 | TCMSP、HERB |
| Diosmetin | 31.13795 | 0.27442 | TCMSP、HERB |
| 5,7-dihydroxy-2-(3-hydroxy-4-methoxyphenyl)chroman-4-one | 47.73644 | 0.27226 | TCMSP、HERB |
| luteolin | 36.16263 | 0.24552 | TCMSP、HERB |
| Hesperetin | 70.31 | 0.27 | HERB |
| Sainfuran | 79.91 | 0.23 | HERB |
| Vestitone | 52.83 | 0.24 | HERB |
| (2aR,5S,6aS,6bS,8aS,8bS,11aS,12aR,12bR)-10-isopentyl-6a,8a,9-trimethyl-2,2a,3,4,5,6,6a,6b,7,8,8a,8b,11a,12,12a,12b-hexadecahydro-1H-naphtho[2',1':4,5]indeno[2,1-b]furan-5-ol | 39.2139 | 0.84447 | TCMSP、HERB |
| (3R,8S,9S,10R,13R,14R,17S)-17-((2S,5R)-5-ethyl-6-methylheptan-2-yl)-3-hydroxy-10,13-dimethyl-3,4,8,9,10,11,12,13,14,15,16,17-dodecahydro-1H-cyclopenta[a]phenanthren-7(2H)-one | 40.92701 | 0.79013 | TCMSP、HERB |
| (3R,7R,8S,9S,10S,13R,14S,17R)-17-((2R,5S)-5-ethyl-6-methylheptan-2-yl)-3,10-dimethyl-2,3,4,7,8,9,10,11,12,13,14,15,16,17-tetradecahydro-1H-cyclopenta[a]phenanthren-7-ol | 34.21133 | 0.76401 | TCMSP、HERB |
| (2aR,2'S,4R,4'R,5'S,6aS,6bS,8aS,8bR,9S,11aR,12aR,12bR)-4,4'-dihydroxy-5',6a,8a,9-tetramethylicosahydro-1H-spiro[pentaleno[2,1-a]phenanthrene-10,2'-pyran]-8(2H)-one | 58.73947 | 0.75597 | TCMSP、HERB |
| sitosterol | 36.91391 | 0.7512 | TCMSP、HERB |
| \u03b2-sitosterol-\u03b2-D-glucopyranoside | 32.4138 | 0.70881 | TCMSP、HERB |
| isorhamnetin | 49.60438 | 0.306 | TCMSP、HERB |
| terrestriamide | 114.088 | 0.29408 | TCMSP、HERB |
| (2aR,2'R,4R,6aR,6bS,8aS,8bR,11aS,12aR,12bR)-4-((S)-2-(2,6-dimethylphenyl)propoxy)-5',5',6a,8a-tetramethyl-8-methylenedocosahydro-1H-spiro[pentaleno[2,1-a]phenanthrene-10,2'-pyran] | 59.48672 | 0.28089 | TCMSP、HERB |
| (Z)-3-(4-hydroxy-3-methoxy-phenyl)-N-[2-(4-hydroxyphenyl)ethyl]acrylamide | 118.3477 | 0.26399 | TCMSP、HERB |
| kaempferol | 41.88225 | 0.24066 | TCMSP、HERB |
| (Z)-3-(3,4-dihydroxyphenyl)-N-[2-(4-hydroxyphenyl)ethyl]acrylamide | 113.2505 | 0.23596 | TCMSP、HERB |
| 9,10-dimethoxypterocarpan-3-O-\u03b2-D-glucoside | 36.73669 | 0.9243 | TCMSP、HERB |
| (3S,8S,9S,10R,13R,14S,17R)-10,13-dimethyl-17-[(2R,5S)-5-propan-2-yloctan-2-yl]-2,3,4,7,8,9,11,12,14,15,16,17-dodecahydro-1H-cyclopenta[a]phenanthren-3-ol | 36.22847 | 0.78288 | TCMSP、HERB |
| Mairin | 55.37707 | 0.7761 | TCMSP、HERB |
| hederagenin | 36.91391 | 0.75072 | TCMSP、HERB |
| FA | 68.96044 | 0.7057 | TCMSP、HERB |
| 5'-hydroxyiso-muronulatol-2',5'-di-O-glucoside | 41.71767 | 0.69251 | TCMSP、HERB |
| Bifendate | 31.09782 | 0.66553 | TCMSP、HERB |
| isomucronulatol-7,2'-di-O-glucosiole | 49.28106 | 0.62065 | TCMSP、HERB |
| 1,7-Dihydroxy-3,9-dimethoxy pterocarpene | 39.04541 | 0.47943 | TCMSP、HERB |
| 3,9-di-O-methylnissolin | 53.74153 | 0.47573 | TCMSP、HERB |
| (6aR,11aR)-9,10-dimethoxy-6a,11a-dihydro-6H-benzofurano[3,2-c]chromen-3-ol | 64.25545 | 0.42486 | TCMSP、HERB |
| isorhamnetin | 49.60438 | 0.306 | TCMSP、HERB |
| 7-O-methylisomucronulatol | 74.68614 | 0.29792 | TCMSP、HERB |
| isoflavanone | 109.9867 | 0.29572 | TCMSP、HERB |
| Jaranol | 50.82882 | 0.29148 | TCMSP、HERB |
| quercetin | 46.43335 | 0.27525 | TCMSP、HERB |
| (3R)-3-(2-hydroxy-3,4-dimethoxyphenyl)chroman-7-ol | 67.66748 | 0.26479 | TCMSP、HERB |
| Calycosin | 47.75183 | 0.24278 | TCMSP、HERB |
| kaempferol | 41.88225 | 0.24066 | TCMSP、HERB |
| formononetin | 69.67388 | 0.21202 | TCMSP、HERB |
| (-)-Medicocarpin | 40.99397 | 0.95059 | TCMSP、HERB |
| Kanzonol F | 32.46833 | 0.89364 | TCMSP、HERB |
| Xambioona | 54.84916 | 0.87419 | TCMSP、HERB |
| Isoglycyrol | 44.69923 | 0.83845 | TCMSP、HERB |
| glycyroside | 37.25032 | 0.79156 | TCMSP、HERB |
| Gancaonin H | 50.10372 | 0.78416 | TCMSP、HERB |
| Mairin | 55.37707 | 0.7761 | TCMSP、HERB |
| sitosterol | 36.91391 | 0.7512 | TCMSP、HERB |
| liquiritin | 65.69011 | 0.73893 | TCMSP、HERB |
| shinpterocarpin | 80.29528 | 0.72746 | TCMSP、HERB |
| (2S)-2-[4-hydroxy-3-(3-methylbut-2-enyl)phenyl]-8,8-dimethyl-2,3-dihydropyrano[2,3-f]chromen-4-one | 31.78703 | 0.72403 | TCMSP、HERB |
| 18\u03b1-hydroxyglycyrrhetic acid | 41.16139 | 0.7091 | TCMSP、HERB |
| Glycyrol | 90.77578 | 0.66819 | TCMSP、HERB |
| licopyranocoumarin | 80.36001 | 0.6535 | TCMSP、HERB |
| 1-Methoxyphaseollidin | 69.98098 | 0.63739 | TCMSP、HERB |
| (2S)-6-(2,4-dihydroxyphenyl)-2-(2-hydroxypropan-2-yl)-4-methoxy-2,3-dihydrofuro[3,2-g]chromen-7-one | 60.25041 | 0.63433 | TCMSP、HERB |
| Glycyrrhiza flavonol A | 41.27528 | 0.59512 | TCMSP、HERB |
| Glyasperins M | 72.67081 | 0.59274 | TCMSP、HERB |
| Licoagrocarpin | 58.8139 | 0.58498 | TCMSP、HERB |
| Phaseol | 78.76622 | 0.57867 | TCMSP、HERB |
| 3'-Hydroxy-4'-O-Methylglabridin | 43.71495 | 0.57406 | TCMSP、HERB |
| 3'-Methoxyglabridin | 46.16151 | 0.57393 | TCMSP、HERB |
| euchrenone | 30.28726 | 0.57386 | TCMSP、HERB |
| Semilicoisoflavone B | 48.77755 | 0.54732 | TCMSP、HERB |
| 3,22-Dihydroxy-11-oxo-delta(12)-oleanene-27-alpha-methoxycarbonyl-29-oic acid | 34.31942 | 0.54718 | TCMSP、HERB |
| Licoisoflavone B | 38.92871 | 0.54714 | TCMSP、HERB |
| licoisoflavanone | 52.46625 | 0.54488 | TCMSP、HERB |
| Inermine | 75.18306 | 0.53754 | TCMSP、HERB |
| glyasperin F | 75.8368 | 0.53514 | TCMSP、HERB |
| 1,3-dihydroxy-8,9-dimethoxy-6-benzofurano[3,2-c]chromenone | 62.90135 | 0.52759 | TCMSP、HERB |
| 2-[(3R)-8,8-dimethyl-3,4-dihydro-2H-pyrano[6,5-f]chromen-3-yl]-5-methoxyphenol | 36.21429 | 0.52122 | TCMSP、HERB |
| kanzonols W | 50.48008 | 0.51704 | TCMSP、HERB |
| Glabrone | 52.51217 | 0.49645 | TCMSP、HERB |
| Licoagroisoflavone | 57.28224 | 0.48679 | TCMSP、HERB |
| Glycyrin | 52.60657 | 0.47466 | TCMSP、HERB |
| Licoricone | 63.57846 | 0.4712 | TCMSP、HERB |
| Glabridin | 53.24514 | 0.46967 | TCMSP、HERB |
| Gancaonin B | 48.7944 | 0.44924 | TCMSP、HERB |
| Phaseolinisoflavan | 32.00811 | 0.44538 | TCMSP、HERB |
| Glabrene | 46.26686 | 0.43902 | TCMSP、HERB |
| glyasperin B | 65.22439 | 0.43851 | TCMSP、HERB |
| 3-(2,4-dihydroxyphenyl)-8-(1,1-dimethylprop-2-enyl)-7-hydroxy-5-methoxy-coumarin | 59.62247 | 0.42894 | TCMSP、HERB |
| 1,3-dihydroxy-9-methoxy-6-benzofurano[3,2-c]chromenone | 48.14154 | 0.42831 | TCMSP、HERB |
| Isotrifoliol | 31.94479 | 0.42422 | TCMSP、HERB |
| Isolicoflavonol | 45.16999 | 0.41859 | TCMSP、HERB |
| Licoisoflavone | 41.61022 | 0.41646 | TCMSP、HERB |
| 2-(3,4-dihydroxyphenyl)-5,7-dihydroxy-6-(3-methylbut-2-enyl)chromone | 44.15196 | 0.41482 | TCMSP、HERB |
| Sigmoidin-B | 34.88109 | 0.41455 | TCMSP、HERB |
| 3-(3,4-dihydroxyphenyl)-5,7-dihydroxy-8-(3-methylbut-2-enyl)chromone | 66.37125 | 0.41392 | TCMSP、HERB |
| 6-prenylated eriodictyol | 39.22383 | 0.41259 | TCMSP、HERB |
| 5,7-dihydroxy-3-(4-methoxyphenyl)-8-(3-methylbut-2-enyl)chromone | 30.48878 | 0.41002 | TCMSP、HERB |
| 8-prenylated eriodictyol | 53.79476 | 0.40383 | TCMSP、HERB |
| Gancaonin A | 51.07519 | 0.40378 | TCMSP、HERB |
| Glyasperin C | 45.56381 | 0.39947 | TCMSP、HERB |
| Gancaonin G | 60.43521 | 0.39404 | TCMSP、HERB |
| 8-(6-hydroxy-2-benzofuranyl)-2,2-dimethyl-5-chromenol | 58.43728 | 0.38106 | TCMSP、HERB |
| Eurycarpin A | 43.27728 | 0.37429 | TCMSP、HERB |
| dehydroglyasperins C | 53.82326 | 0.37006 | TCMSP、HERB |
| Lupiwighteone | 51.63569 | 0.36739 | TCMSP、HERB |
| Licocoumarone | 33.21085 | 0.3568 | TCMSP、HERB |
| Glyzaglabrin | 61.06889 | 0.35347 | TCMSP、HERB |
| (E)-1-(2,4-dihydroxyphenyl)-3-(2,2-dimethylchromen-6-yl)prop-2-en-1-one | 39.61686 | 0.35077 | TCMSP、HERB |
| Glepidotin A | 44.72187 | 0.34685 | TCMSP、HERB |
| Glepidotin B | 64.46292 | 0.34485 | TCMSP、HERB |
| Medicarpin | 49.21982 | 0.3351 | TCMSP、HERB |
| Quercetin der. | 46.44939 | 0.3343 | TCMSP、HERB |
| Inflacoumarin A | 39.7091 | 0.32613 | TCMSP、HERB |
| licochalcone G | 49.25496 | 0.32325 | TCMSP、HERB |
| (2S)-7-hydroxy-2-(4-hydroxyphenyl)-8-(3-methylbut-2-enyl)chroman-4-one | 36.56537 | 0.32291 | TCMSP、HERB |
| Glabranin | 52.89566 | 0.31208 | TCMSP、HERB |
| (E)-3-[3,4-dihydroxy-5-(3-methylbut-2-enyl)phenyl]-1-(2,4-dihydroxyphenyl)prop-2-en-1-one | 46.26792 | 0.3062 | TCMSP、HERB |
| isorhamnetin | 49.60438 | 0.306 | TCMSP、HERB |
| Odoratin | 49.94822 | 0.30487 | TCMSP、HERB |
| Jaranol | 50.82882 | 0.29148 | TCMSP、HERB |
| licochalcone a | 40.78965 | 0.28517 | TCMSP、HERB |
| quercetin | 46.43335 | 0.27525 | TCMSP、HERB |
| licorice glycoside E | 32.88743 | 0.27218 | TCMSP、HERB |
| 7,2',4'-trihydroxy\uff0d5-methoxy-3\uff0darylcoumarin | 83.71437 | 0.27136 | TCMSP、HERB |
| 7-Acetoxy-2-methylisoflavone | 38.92333 | 0.26217 | TCMSP、HERB |
| Calycosin | 47.75183 | 0.24278 | TCMSP、HERB |
| kaempferol | 41.88225 | 0.24066 | TCMSP、HERB |
| formononetin | 69.67388 | 0.21202 | TCMSP、HERB |
| naringenin | 59.2939 | 0.21128 | TCMSP、HERB |
| HMO | 38.36542 | 0.21067 | TCMSP、HERB |
| Vestitol | 74.65519 | 0.20935 | TCMSP、HERB |
| 7-Methoxy-2-methyl isoflavone | 42.56474 | 0.19946 | TCMSP、HERB |
| icos-5-enoic acid | 30.70294 | 0.19725 | TCMSP、HERB |
| gadelaidic acid | 30.70294 | 0.19725 | TCMSP、HERB |
| Licochalcone B | 76.75735 | 0.1935 | TCMSP、HERB |
| Glypallichalcone | 61.59706 | 0.18993 | TCMSP、HERB |
| DFV | 32.76272 | 0.18316 | TCMSP、HERB |
| (2R)-7-hydroxy-2-(4-hydroxyphenyl)chroman-4-one | 71.12299 | 0.18303 | TCMSP、HERB |
| 18α-hydroxyglycyrrhetic acid | 41.16 | 0.71 | HERB |
| hispidulin | 30.97 | 0.27 | HERB |
| isogosferol | 30.07 | 0.25 | HERB |
| Isoramanone | 39.97 | 0.51 | HERB |
| liquiritigenin | 32.76 | 0.18 | HERB |
| sigmoidin b | 34.88 | 0.41 | HERB |
| beta-sitosterol | 36.91391 | 0.75123 | TCMSP、HERB |
| sitosterol | 36.91391 | 0.7512 | TCMSP、HERB |
| (2R,3R)-3-(4-hydroxy-3-methoxy-phenyl)-5-methoxy-2-methylol-2,3-dihydropyrano[5,6-h][1,4]benzodioxin-9-one | 68.8256 | 0.66236 | TCMSP、HERB |
| anomalin | 59.65406 | 0.65656 | TCMSP、HERB |
| ledebouriellol | 32.05015 | 0.506 | TCMSP、HERB |
| Decursin | 39.26721 | 0.38286 | TCMSP、HERB |
| divaricatol | 31.65264 | 0.38004 | TCMSP、HERB |
| divaricatacid | 86.99614 | 0.32487 | TCMSP、HERB |
| phelloptorin | 43.38604 | 0.27878 | TCMSP、HERB |
| Phellopterin | 40.18556 | 0.27878 | TCMSP、HERB |
| 11-hydroxy-sec-o-beta-d-glucosylhamaudol_qt | 50.24351 | 0.26931 | TCMSP、HERB |
| 5-O-Methylvisamminol | 37.99006 | 0.2494 | TCMSP、HERB |
| wogonin | 30.68457 | 0.22942 | TCMSP、HERB |
| methyl icosa-11,14-dienoate | 39.66706 | 0.22908 | TCMSP、HERB |
| isoimperatorin | 45.46425 | 0.22524 | TCMSP、HERB |
| Ammidin | 34.54856 | 0.22355 | TCMSP、HERB |
| Prangenidin | 36.31449 | 0.21938 | TCMSP、HERB |
| Mandenol | 41.9962 | 0.19321 | TCMSP、HERB |
| 5alpha-Stigmastan-3,6-dione | 33.1154 | 0.79021 | TCMSP、HERB |
| Daturilin | 50.36513 | 0.76801 | TCMSP、HERB |
| Taraxerol | 38.40254 | 0.76677 | TCMSP、HERB |
| (8S,9S,10R,13R,14S,17R)-17-[(E,2R,5S)-5-ethyl-6-methylhept-3-en-2-yl]-10,13-dimethyl-1,2,4,7,8,9,11,12,14,15,16,17-dodecahydrocyclopenta[a]phenanthren-3-one | 45.40462 | 0.76174 | TCMSP、HERB |
| Stigmasterol | 43.82985 | 0.75665 | TCMSP、HERB |
| ZINC03978781 | 43.82985 | 0.75647 | TCMSP、HERB |
| poriferasta-7,22E-dien-3beta-ol | 42.97937 | 0.75555 | TCMSP、HERB |
| Spinasterol | 42.97937 | 0.75534 | TCMSP、HERB |
| stigmast-7-enol | 37.42312 | 0.75133 | TCMSP、HERB |
| 11-Hydroxyrankinidine | 40.00276 | 0.66203 | TCMSP、HERB |
| Chrysanthemaxanthin | 38.72398 | 0.58352 | TCMSP、HERB |
| 3-beta-Hydroxymethyllenetanshiquinone | 32.16103 | 0.40894 | TCMSP、HERB |
| Spinoside A | 39.96686 | 0.40288 | TCMSP、HERB |
| Diop | 43.59333 | 0.39247 | TCMSP、HERB |
| Frutinone A | 65.90373 | 0.34184 | TCMSP、HERB |
| 7-(beta-Xylosyl)cephalomannine_qt | 38.32746 | 0.28646 | TCMSP、HERB |
| Perlolyrine | 65.94775 | 0.2747 | TCMSP、HERB |
| luteolin | 36.16263 | 0.24552 | TCMSP、HERB |
| glycitein | 50.47891 | 0.23826 | TCMSP、HERB |
| methyl icosa-11,14-dienoate | 39.66706 | 0.22908 | TCMSP、HERB |
| 7-Methoxy-2-methyl isoflavone | 42.56474 | 0.19946 | TCMSP、HERB |
| Stigmasterol | 43.82985 | 0.75665 | TCMSP、HERB |
| beta-sitosterol | 36.91391 | 0.75123 | TCMSP、HERB |
| \u03b1-amyrin | 39.51209 | 0.76221 | TCMSP、HERB |
| Poriferasterol | 43.82985 | 0.75596 | TCMSP、HERB |
| Baicalin | 40.12361 | 0.75264 | TCMSP、HERB |
| poriferast-5-en-3beta-ol | 36.91391 | 0.75034 | TCMSP、HERB |
| salvianolic acid j | 43.37605 | 0.72497 | TCMSP、HERB |
| 6-o-syringyl-8-o-acetyl shanzhiside methyl ester | 46.69066 | 0.71145 | TCMSP、HERB |
| przewalskin a | 37.1065 | 0.64901 | TCMSP、HERB |
| salvianolic acid g | 45.56486 | 0.60602 | TCMSP、HERB |
| Danshenol B | 57.95088 | 0.55764 | TCMSP、HERB |
| dan-shexinkum d | 38.88302 | 0.55453 | TCMSP、HERB |
| Danshenol A | 56.96525 | 0.52172 | TCMSP、HERB |
| przewaquinone f | 40.30788 | 0.45925 | TCMSP、HERB |
| (6S)-6-hydroxy-1-methyl-6-methylol-8,9-dihydro-7H-naphtho[8,7-g]benzofuran-10,11-quinone | 75.38588 | 0.4551 | TCMSP、HERB |
| Tanshindiol B | 42.66581 | 0.45303 | TCMSP、HERB |
| Przewaquinone E | 42.85485 | 0.45301 | TCMSP、HERB |
| (6S,7R)-6,7-dihydroxy-1,6-dimethyl-8,9-dihydro-7H-naphtho[8,7-g]benzofuran-10,11-dione | 41.31046 | 0.453 | TCMSP、HERB |
| tanshinaldehyde | 52.4747 | 0.45196 | TCMSP、HERB |
| (6S)-6-(hydroxymethyl)-1,6-dimethyl-8,9-dihydro-7H-naphtho[8,7-g]benzofuran-10,11-dione | 65.25894 | 0.44871 | TCMSP、HERB |
| 3\u03b1-hydroxytanshinone\u2161a | 44.92934 | 0.44272 | TCMSP、HERB |
| przewalskin b | 110.324 | 0.43809 | TCMSP、HERB |
| miltionone \u2161 | 71.0297 | 0.43711 | TCMSP、HERB |
| formyltanshinone | 73.44462 | 0.41736 | TCMSP、HERB |
| Przewaquinone B | 62.24006 | 0.41374 | TCMSP、HERB |
| 3-beta-Hydroxymethyllenetanshiquinone | 32.16103 | 0.40894 | TCMSP、HERB |
| przewaquinone c | 55.74167 | 0.40408 | TCMSP、HERB |
| Dehydrotanshinone II A | 43.76229 | 0.40019 | TCMSP、HERB |
| tanshinone iia | 49.8873 | 0.39781 | TCMSP、HERB |
| Isotanshinone II | 49.91603 | 0.39674 | TCMSP、HERB |
| 2-(4-hydroxy-3-methoxyphenyl)-5-(3-hydroxypropyl)-7-methoxy-3-benzofurancarboxaldehyde | 62.78415 | 0.39628 | TCMSP、HERB |
| cryptotanshinone | 52.34196 | 0.39555 | TCMSP、HERB |
| isocryptotanshi-none | 54.98193 | 0.39449 | TCMSP、HERB |
| Salvilenone | 30.38365 | 0.37639 | TCMSP、HERB |
| miltipolone | 36.55611 | 0.36803 | TCMSP、HERB |
| 1-methyl-8,9-dihydro-7H-naphtho[5,6-g]benzofuran-6,10,11-trione | 34.72082 | 0.36634 | TCMSP、HERB |
| Methylenetanshinquinone | 37.07319 | 0.36017 | TCMSP、HERB |
| dihydrotanshinone\u2160 | 45.04328 | 0.36015 | TCMSP、HERB |
| 1,2,5,6-tetrahydrotanshinone | 38.74539 | 0.35791 | TCMSP、HERB |
| (2R)-3-(3,4-dihydroxyphenyl)-2-[(Z)-3-(3,4-dihydroxyphenyl)acryloyl]oxy-propionic acid | 109.3805 | 0.35119 | TCMSP、HERB |
| neocryptotanshinone | 52.488 | 0.32306 | TCMSP、HERB |
| dihydrotanshinlactone | 38.68477 | 0.32227 | TCMSP、HERB |
| miltionone \u2160 | 49.68439 | 0.32125 | TCMSP、HERB |
| (E)-3-[2-(3,4-dihydroxyphenyl)-7-hydroxy-benzofuran-4-yl]acrylic acid | 48.24363 | 0.31229 | TCMSP、HERB |
| prolithospermic acid | 64.37096 | 0.31017 | TCMSP、HERB |
| danshenspiroketallactone | 50.43128 | 0.3067 | TCMSP、HERB |
| epidanshenspiroketallactone | 68.27316 | 0.30549 | TCMSP、HERB |
| tanshinone \u2165 | 45.63731 | 0.29549 | TCMSP、HERB |
| 5,6-dihydroxy-7-isopropyl-1,1-dimethyl-2,3-dihydrophenanthren-4-one | 33.76525 | 0.28585 | TCMSP、HERB |
| deoxyneocryptotanshinone | 49.40035 | 0.28555 | TCMSP、HERB |
| microstegiol | 39.61229 | 0.27734 | TCMSP、HERB |
| sugiol | 36.11353 | 0.27648 | TCMSP、HERB |
| NSC 122421 | 34.49292 | 0.27645 | TCMSP、HERB |
| (Z)-3-[2-[(E)-2-(3,4-dihydroxyphenyl)vinyl]-3,4-dihydroxy-phenyl]acrylic acid | 88.53602 | 0.25869 | TCMSP、HERB |
| digallate | 61.84862 | 0.25635 | TCMSP、HERB |
| Miltirone | 38.75699 | 0.25418 | TCMSP、HERB |
| C09092 | 36.06949 | 0.2474 | TCMSP、HERB |
| luteolin | 36.16263 | 0.24552 | TCMSP、HERB |
| salviolone | 31.72415 | 0.23568 | TCMSP、HERB |
| miltirone \u2161 | 44.95107 | 0.23537 | TCMSP、HERB |
| neocryptotanshinone ii | 39.46299 | 0.23157 | TCMSP、HERB |
| 2-isopropyl-8-methylphenanthrene-3,4-dione | 40.86015 | 0.22897 | TCMSP、HERB |
| salvilenone \u2160 | 32.43471 | 0.22895 | TCMSP、HERB |
| 4-methylenemiltirone | 34.34868 | 0.22726 | TCMSP、HERB |
| isoimperatorin | 45.46425 | 0.22524 | TCMSP、HERB |
| sclareol | 43.67068 | 0.2058 | TCMSP、HERB |
| manool | 45.04432 | 0.20208 | TCMSP、HERB |
| beta-sitosterol | 36.91391 | 0.75123 | TCMSP、HERB |
| sitosterol | 36.91391 | 0.7512 | TCMSP、HERB |
| meso-1,4-Bis-(4-hydroxy-3-methoxyphenyl)-2,3-dimethylbutane | 31.31843 | 0.26047 | TCMSP、HERB |
| (-)-catechin | 49.67639 | 0.24162 | TCMSP、HERB |
| 2-(4-hydroxyphenyl)ethyl (E)-3-(4-hydroxyphenyl)prop-2-enoate | 93.35552 | 0.20507 | TCMSP、HERB |
| Peroxyergosterol | 44.39152 | 0.82 | TCMSP、HERB |
| 24-Ethylcholest-4-en-3-one | 36.08361 | 0.75703 | TCMSP、HERB |
| Stigmasterol | 43.82985 | 0.75665 | TCMSP、HERB |
| (3S,8S,9S,10R,13R,14S,17R)-17-[(1S,4R)-4-ethyl-1,5-dimethylhexyl]-10,13-dimethyl-2,3,4,7,8,9,11,12,14,15,16,17-dodecahydro-1H-cyclopenta[a]phenanthren-3-ol | 36.91391 | 0.75147 | TCMSP、HERB |
| beta-sitosterol | 36.91391 | 0.75123 | TCMSP、HERB |
| sitosterol | 36.91391 | 0.7512 | TCMSP、HERB |
| Cynarin(e) | 31.7585 | 0.67849 | TCMSP、HERB |
| (2R,3R)-3-(4-hydroxy-3-methoxy-phenyl)-5-methoxy-2-methylol-2,3-dihydropyrano[5,6-h][1,4]benzodioxin-9-one | 68.8256 | 0.66236 | TCMSP、HERB |
| carboxyatractyloside | 39.9687 | 0.47025 | TCMSP、HERB |
| Moupinamide | 86.71216 | 0.26454 | TCMSP、HERB |
| aloe-emodin | 83.37964 | 0.2409 | TCMSP、HERB |
| isobavachin | 54.44 | 0.32 | HERB |
| stigmasterol | 43.83 | 0.76 | HERB |
| catechin | 54.83 | 0.24 | HERB |
| n-trans-feruloyltyramine | 86.71 | 0.26 | HERB |
| Physciondiglucoside | 41.65 | 0.63 | HERB |
| rhein | 47.07 | 0.28 | HERB |

1. **Supplementary Table 2. 349 target of vitiligo from FHB**

| NO. | Target |
| --- | --- |
| 1 | PGR |
| 2 | NCOA2 |
| 3 | PTGS2 |
| 4 | ESR1 |
| 5 | CA2 |
| 6 | NOS2 |
| 7 | PTGS1 |
| 8 | AR |
| 9 | PRSS1 |
| 10 | DPP4 |
| 11 | NCOA1 |
| 12 | RELA |
| 13 | AKT1 |
| 14 | VEGFA |
| 15 | BCL2 |
| 16 | FOS |
| 17 | BAX |
| 18 | MMP9 |
| 19 | CASP3 |
| 20 | TP53 |
| 21 | HIF1A |
| 22 | FOSL1 |
| 23 | FOSL2 |
| 24 | CDK1 |
| 25 | CCNB1 |
| 26 | MPO |
| 27 | AHR |
| 28 | IGF2 |
| 29 | CYCS |
| 30 | NFATC1 |
| 31 | TDRD7 |
| 32 | EGLN1 |
| 33 | NOX5 |
| 34 | FABP5 |
| 35 | APOD |
| 36 | GABRA1 |
| 37 | CASP9 |
| 38 | MMP2 |
| 39 | JUN |
| 40 | CASP8 |
| 41 | MMP1 |
| 42 | HMOX1 |
| 43 | CYP3A4 |
| 44 | CYP1A2 |
| 45 | CAV1 |
| 46 | CTNNB1 |
| 47 | MYC |
| 48 | CASP7 |
| 49 | F3 |
| 50 | GJA1 |
| 51 | MMP10 |
| 52 | KCNH2 |
| 53 | DRD1 |
| 54 | CHRM3 |
| 55 | CHRM1 |
| 56 | SCN5A |
| 57 | CHRM4 |
| 58 | ADRA1D |
| 59 | CHRM2 |
| 60 | ADRA1B |
| 61 | ADRB2 |
| 62 | CHRNA2 |
| 63 | SLC6A4 |
| 64 | OPRM1 |
| 65 | PRKCA |
| 66 | PON1 |
| 67 | MAP2 |
| 68 | ACHE |
| 69 | SLC6A2 |
| 70 | F7 |
| 71 | IKBKB |
| 72 | AHSA1 |
| 73 | MAPK8 |
| 74 | XDH |
| 75 | STAT1 |
| 76 | PPARG |
| 77 | CYP1A1 |
| 78 | ICAM1 |
| 79 | SELE |
| 80 | VCAM1 |
| 81 | NR1I2 |
| 82 | CYP1B1 |
| 83 | ALOX5 |
| 84 | HAS2 |
| 85 | GSTP1 |
| 86 | PSMD3 |
| 87 | SLC2A4 |
| 88 | NR1I3 |
| 89 | INSR |
| 90 | DIO1 |
| 91 | PPP3CA |
| 92 | GSTM1 |
| 93 | GSTM2 |
| 94 | AKR1C3 |
| 95 | SLPI |
| 96 | NR3C2 |
| 97 | ADH1C |
| 98 | IGHG1 |
| 99 | RXRA |
| 100 | ADRA2A |
| 101 | SLC6A3 |
| 102 | AKR1B1 |
| 103 | PLAU |
| 104 | LTA4H |
| 105 | CTRB1 |
| 106 | ADRB1 |
| 107 | EGFR |
| 108 | CCND1 |
| 109 | BCL2L1 |
| 110 | CDKN1A |
| 111 | MAPK1 |
| 112 | IL10 |
| 113 | RB1 |
| 114 | CDK4 |
| 115 | IL6 |
| 116 | NFKBIA |
| 117 | TOP1 |
| 118 | MDM2 |
| 119 | PCNA |
| 120 | ERBB2 |
| 121 | MCL1 |
| 122 | BIRC5 |
| 123 | IL2 |
| 124 | TYR |
| 125 | IFNG |
| 126 | IL4 |
| 127 | TOP2A |
| 128 | XIAP |
| 129 | CD40LG |
| 130 | PTGES |
| 131 | NUF2 |
| 132 | ADCY2 |
| 133 | MET |
| 134 | MMP3 |
| 135 | EIF6 |
| 136 | EGF |
| 137 | ELK1 |
| 138 | POR |
| 139 | ODC1 |
| 140 | RAF1 |
| 141 | RUNX1T1 |
| 142 | HSPA5 |
| 143 | ACACA |
| 144 | IL1B |
| 145 | CCL2 |
| 146 | PTGER3 |
| 147 | CXCL8 |
| 148 | PRKCB |
| 149 | DUOX2 |
| 150 | NOS3 |
| 151 | HSPB1 |
| 152 | SULT1E1 |
| 153 | MGAM |
| 154 | PLAT |
| 155 | THBD |
| 156 | SERPINE1 |
| 157 | IL1A |
| 158 | NCF1 |
| 159 | ABCG2 |
| 160 | NFE2L2 |
| 161 | CXCL11 |
| 162 | CXCL2 |
| 163 | DCAF5 |
| 164 | CHEK2 |
| 165 | CLDN4 |
| 166 | PPARA |
| 167 | PPARD |
| 168 | HSF1 |
| 169 | CRP |
| 170 | CXCL10 |
| 171 | CHUK |
| 172 | SPP1 |
| 173 | RUNX2 |
| 174 | RASSF1 |
| 175 | E2F1 |
| 176 | E2F2 |
| 177 | ACP3 |
| 178 | CTSD |
| 179 | IGFBP3 |
| 180 | IRF1 |
| 181 | ERBB3 |
| 182 | PCOLCE |
| 183 | NPEPPS |
| 184 | HK2 |
| 185 | NKX3-1 |
| 186 | RASA1 |
| 187 | ESR2 |
| 188 | MAPK14 |
| 189 | GSK3B |
| 190 | CDK2 |
| 191 | CCNA2 |
| 192 | PYGM |
| 193 | CHEK1 |
| 194 | GRIA2 |
| 195 | OLR1 |
| 196 | NR3C1 |
| 197 | DPEP1 |
| 198 | CHRM5 |
| 199 | HTR3A |
| 200 | OPRD1 |
| 201 | ADRA2C |
| 202 | STAT3 |
| 203 | ADRA2B |
| 204 | GABRG3 |
| 205 | GABRE |
| 206 | FASN |
| 207 | EDNRA |
| 208 | NPM1 |
| 209 | ECE1 |
| 210 | PARP4 |
| 211 | CALCR |
| 212 | ITGB3 |
| 213 | PKIA |
| 214 | TUBB1 |
| 215 | MMP13 |
| 216 | MMP8 |
| 217 | PRKCE |
| 218 | PRKCD |
| 219 | KDR |
| 220 | MAPK10 |
| 221 | ATP5F1B |
| 222 | MT-ND6 |
| 223 | HSD3B2 |
| 224 | HSD3B1 |
| 225 | MAPK3 |
| 226 | LDLR |
| 227 | BAD |
| 228 | CAT |
| 229 | MTTP |
| 230 | APOB |
| 231 | PLB1 |
| 232 | HMGCR |
| 233 | CYP19A1 |
| 234 | UGT1A1 |
| 235 | SREBF1 |
| 236 | GSR |
| 237 | ABCC1 |
| 238 | ADIPOQ |
| 239 | SOAT2 |
| 240 | AKR1C1 |
| 241 | GOT1 |
| 242 | ABAT |
| 243 | CES1 |
| 244 | SOAT1 |
| 245 | RXRB |
| 246 | ADH1B |
| 247 | KLF7 |
| 248 | TEP1 |
| 249 | FN1 |
| 250 | CNR2 |
| 251 | MAOB |
| 252 | BRAF |
| 253 | ALDH2 |
| 254 | WEE1 |
| 255 | DNM1 |
| 256 | PRKCZ |
| 257 | THRA |
| 258 | CDK5R1 |
| 259 | CDK5 |
| 260 | THRB |
| 261 | CTSL |
| 262 | HSP90AA1 |
| 263 | ABL1 |
| 264 | SYK |
| 265 | GLI2 |
| 266 | PDK1 |
| 267 | BCHE |
| 268 | DRD2 |
| 269 | DRD3 |
| 270 | BMP1 |
| 271 | RPS6KB1 |
| 272 | CDK1 |
| 273 | CCNE1 |
| 274 | CDK3 |
| 275 | GRK2 |
| 276 | EP300 |
| 277 | DUSP3 |
| 278 | ESRRA |
| 279 | ESRRB |
| 280 | HSD17B2 |
| 281 | HSD17B1 |
| 282 | EPHA2 |
| 283 | EPHB2 |
| 284 | EPHA5 |
| 285 | EPHA4 |
| 286 | EPHA8 |
| 287 | EPHA7 |
| 288 | EPHB3 |
| 289 | EPHA3 |
| 290 | EPHB1 |
| 291 | EPHA1 |
| 292 | MYLK |
| 293 | MTOR |
| 294 | TRPM8 |
| 295 | MMP7 |
| 296 | AKT2 |
| 297 | HPGDS |
| 298 | HDAC1 |
| 299 | ANPEP |
| 300 | FNTA |
| 301 | FNTB |
| 302 | ADAM17 |
| 303 | GRM2 |
| 304 | AGTR1 |
| 305 | ADORA1 |
| 306 | ADORA2A |
| 307 | ROCK2 |
| 308 | CA7 |
| 309 | CA6 |
| 310 | CA14 |
| 311 | CA9 |
| 312 | CA5A |
| 313 | VCP |
| 314 | ATP4B |
| 315 | ATP4A |
| 316 | PDE4D |
| 317 | PDE4C |
| 318 | MMP14 |
| 319 | BRD4 |
| 320 | TNF |
| 321 | BRD9 |
| 322 | PNMT |
| 323 | SLC5A1 |
| 324 | TRAP1 |
| 325 | HSP90B1 |
| 326 | TBXA2R |
| 327 | CFD |
| 328 | ALK |
| 329 | HSP90AB1 |
| 330 | CA12 |
| 331 | MELK |
| 332 | SPHK2 |
| 333 | FTO |
| 334 | ELANE |
| 335 | FNTB |
| 336 | NFKB1 |
| 337 | PIK3CG |
| 338 | KCNMA1 |
| 339 | CALML5 |
| 340 | PRKACA |
| 341 | PDE3A |
| 342 | PIM1 |
| 343 | F2 |
| 344 | NOS |
| 345 | RHO |
| 346 | FHUA |
| 347 | F10 |
| 348 | F9 |
| 349 | HTR2A |

1. **Supplementary Table 3. 1359 target of vitiligo from TTD、OMIM、 DrugBank 、DisGeNET and GeneCards .**

| **NO** | **Target** |
| --- | --- |
| 1 | NLRP1 |
| 2 | PTPN22 |
| 3 | VAMAS6 |
| 4 | AIRE |
| 5 | DSTYK |
| 6 | CAT |
| 7 | AIS3 |
| 8 | TYR |
| 9 | FOXD3 |
| 10 | KITLG |
| 11 | CTLA4 |
| 12 | PMEL |
| 13 | TNF |
| 14 | HLA-B |
| 15 | TYRP1 |
| 16 | DCT |
| 17 | MC1R |
| 18 | MLANA |
| 19 | CCR6 |
| 20 | IL17A |
| 21 | MCHR1 |
| 22 | MITF |
| 23 | PRDX5 |
| 24 | IL2 |
| 25 | CD4 |
| 26 | HLA-DRB1 |
| 27 | POMC |
| 28 | FGF2 |
| 29 | CD8A |
| 30 | FAS |
| 31 | PAH |
| 32 | PCBD1 |
| 33 | NPY |
| 34 | HLA-A |
| 35 | PDCD1 |
| 36 | FBXO11 |
| 37 | HLA-DQB1 |
| 38 | DDC |
| 39 | TPO |
| 40 | PLCG2 |
| 41 | ICOSLG |
| 42 | HLA-C |
| 43 | HLA-DQA1 |
| 44 | CIITA |
| 45 | C4B |
| 46 | CD274 |
| 47 | MIR518A1 |
| 48 | NBN |
| 49 | IL10 |
| 50 | IL2RA |
| 51 | MSH3 |
| 52 | ATM |
| 53 | IFNG |
| 54 | FOXP3 |
| 55 | GZMB |
| 56 | IL6 |
| 57 | MYG1 |
| 58 | ACE |
| 59 | TRAC |
| 60 | RAG1 |
| 61 | VDR |
| 62 | IL4 |
| 63 | FASLG |
| 64 | RAG2 |
| 65 | AA1 |
| 66 | AA2 |
| 67 | AIS4 |
| 68 | CXCL10 |
| 69 | ACP5 |
| 70 | GSTM1 |
| 71 | DCLRE1C |
| 72 | TH |
| 73 | GSTT1 |
| 74 | KANSL1 |
| 75 | SLX4 |
| 76 | AIS2 |
| 77 | NFE2L2 |
| 78 | INO80 |
| 79 | TRA |
| 80 | MTHFR |
| 81 | ZNF627 |
| 82 | DDR1 |
| 83 | CEP43 |
| 84 | PSMB8 |
| 85 | IL1RN |
| 86 | IKZF4 |
| 87 | COMT |
| 88 | SOD1 |
| 89 | EDN1 |
| 90 | CXCR3 |
| 91 | SMOC2 |
| 92 | ICAM1 |
| 93 | SUOX |
| 94 | GPX1 |
| 95 | LRBA |
| 96 | FOXD3-AS1 |
| 97 | IL1B |
| 98 | UBASH3A |
| 99 | HLA-DRB4 |
| 100 | CXCR4 |
| 101 | POGZ |
| 102 | EMC1 |
| 103 | MT-CO1 |
| 104 | MT-ND4 |
| 105 | MT-CO2 |
| 106 | MT-ND5 |
| 107 | MT-ND6 |
| 108 | MT-ND1 |
| 109 | MT-CO3 |
| 110 | MT-TL1 |
| 111 | MT-TS1 |
| 112 | MT-TF |
| 113 | MT-TH |
| 114 | MT-TS2 |
| 115 | MT-TW |
| 116 | MT-TQ |
| 117 | BTNL2 |
| 118 | PTGS2 |
| 119 | MBL2 |
| 120 | RNASET2 |
| 121 | LPP |
| 122 | CCR5 |
| 123 | TSLP |
| 124 | SIRT1 |
| 125 | TP53 |
| 126 | LIPE |
| 127 | MTX2 |
| 128 | CELA2A |
| 129 | LIPE-AS1 |
| 130 | LOC101930071 |
| 131 | HGF |
| 132 | TAP1 |
| 133 | PDGFRA |
| 134 | TG |
| 135 | RAB5B |
| 136 | ARMC9 |
| 137 | ZMIZ1 |
| 138 | PTPRC |
| 139 | RERE |
| 140 | C1QTNF6 |
| 141 | HCG9 |
| 142 | XBP1 |
| 143 | CLEC11A |
| 144 | CD80 |
| 145 | CXCL9 |
| 146 | MSH6 |
| 147 | CXCR5 |
| 148 | CYP21A2 |
| 149 | ATP8B1 |
| 150 | KCNK12 |
| 151 | HUNK |
| 152 | ASIP |
| 153 | NFKB2 |
| 154 | FZD6 |
| 155 | GLI2 |
| 156 | ABCD1 |
| 157 | MT-ATP6 |
| 158 | MT-TT |
| 159 | MT-TK |
| 160 | MT-TV |
| 161 | MT-TE |
| 162 | MT-TI |
| 163 | MT-TL2 |
| 164 | MT-TN |
| 165 | MT-TA |
| 166 | EMC1-AS1 |
| 167 | MT-TP |
| 168 | HPP1 |
| 169 | LTA |
| 170 | PSMB9 |
| 171 | IFIH1 |
| 172 | GCH1 |
| 173 | GSTP1 |
| 174 | GAD2 |
| 175 | TXNDC5 |
| 176 | RPGRIP1L |
| 177 | TF |
| 178 | TGFB1 |
| 179 | NUDT6 |
| 180 | FOXP1 |
| 181 | SLC44A4 |
| 182 | ESR1 |
| 183 | APEX1 |
| 184 | HLA-G |
| 185 | KLRK1 |
| 186 | GAD1 |
| 187 | IFNA2 |
| 188 | G6PD |
| 189 | NR1H3 |
| 190 | TSBP1 |
| 191 | AQP3 |
| 192 | QDPR |
| 193 | DKK1 |
| 194 | MAOA |
| 195 | INS |
| 196 | HLA-DRA |
| 197 | RBM17 |
| 198 | TSBP1-AS1 |
| 199 | HMOX1 |
| 200 | IL15 |
| 201 | TGFBR2 |
| 202 | FADS1 |
| 203 | STAT3 |
| 204 | ERBB3 |
| 205 | CDK2 |
| 206 | DDX6 |
| 207 | SLC29A3 |
| 208 | CDH23 |
| 209 | DGKA |
| 210 | ATXN2 |
| 211 | BACH2 |
| 212 | PA2G4 |
| 213 | RPS26 |
| 214 | TREH |
| 215 | UNC5B |
| 216 | PHLDB1 |
| 217 | NOS2 |
| 218 | CBS |
| 219 | UVRAG |
| 220 | DEFB1 |
| 221 | SLC1A2 |
| 222 | FANCA |
| 223 | CASP7 |
| 224 | SH2B3 |
| 225 | CD44 |
| 226 | HERC2 |
| 227 | TICAM1 |
| 228 | OCA2 |
| 229 | TOB2 |
| 230 | SLA |
| 231 | CLNK |
| 232 | NRROS |
| 233 | FBXO45 |
| 234 | VCAM1 |
| 235 | KIF1B |
| 236 | CYP11A1 |
| 237 | CDH1 |
| 238 | BCHE |
| 239 | BMP6 |
| 240 | MPO |
| 241 | CXCL8 |
| 242 | KIT |
| 243 | AIS1 |
| 244 | CP |
| 245 | CCN3 |
| 246 | TPH1 |
| 247 | TNC |
| 248 | CCR4 |
| 249 | IL19 |
| 250 | STAT4 |
| 251 | PRF1 |
| 252 | HAVCR2 |
| 253 | MIR196A2 |
| 254 | ITGB1 |
| 255 | HSPA4 |
| 256 | WASF5P |
| 257 | IL33 |
| 258 | CDK5RAP1 |
| 259 | XDH |
| 260 | JAK1 |
| 261 | RIPK1 |
| 262 | PNMT |
| 263 | CRHR1 |
| 264 | CRH |
| 265 | CYP17A1 |
| 266 | HLA-DQA2 |
| 267 | TLR4 |
| 268 | MSRA |
| 269 | MIR21 |
| 270 | MIR211 |
| 271 | AHR |
| 272 | PDE10A |
| 273 | ICA1 |
| 274 | GCHFR |
| 275 | CORO2A |
| 276 | GLRB |
| 277 | GPHN |
| 278 | GLRA1 |
| 279 | CYP1A2 |
| 280 | ABAT |
| 281 | CNTNAP2 |
| 282 | DPP6 |
| 283 | AMPH |
| 284 | LGI1 |
| 285 | GABARAP |
| 286 | TSPO |
| 287 | CSAD |
| 288 | DPYSL5 |
| 289 | PNMA2 |
| 290 | PTEN |
| 291 | PIK3CB |
| 292 | IL13 |
| 293 | CD28 |
| 294 | FOXO3 |
| 295 | LAMB1 |
| 296 | MIR2909 |
| 297 | HNRNPA1P2 |
| 298 | SIAE |
| 299 | TXN |
| 300 | CD46 |
| 301 | RB1 |
| 302 | CD59 |
| 303 | CD14 |
| 304 | REL |
| 305 | FCGR3A |
| 306 | MTRR |
| 307 | CCL20 |
| 308 | KLRD1 |
| 309 | CASP3 |
| 310 | CREB1 |
| 311 | IFNAR1 |
| 312 | S100B |
| 313 | ARNT |
| 314 | CCL5 |
| 315 | LARP7 |
| 316 | IL22 |
| 317 | EIF5B |
| 318 | IL26 |
| 319 | CASR |
| 320 | DGKE |
| 321 | SPTBN2 |
| 322 | CCK |
| 323 | TRAPPC10 |
| 324 | SLAMF8 |
| 325 | MAPK1 |
| 326 | TNFRSF1A |
| 327 | LEF1 |
| 328 | VEGFA |
| 329 | IL10RB |
| 330 | USF1 |
| 331 | SOD3 |
| 332 | IL22RA1 |
| 333 | IL20 |
| 334 | IL20RA |
| 335 | MIR200C |
| 336 | LOC111365141 |
| 337 | TLR2 |
| 338 | NOTCH1 |
| 339 | SIRT3 |
| 340 | SOX5 |
| 341 | SP1 |
| 342 | CD27 |
| 343 | MC4R |
| 344 | CCR8 |
| 345 | CSF2 |
| 346 | LPO |
| 347 | CXCL16 |
| 348 | CXCR6 |
| 349 | CCL22 |
| 350 | TUG1 |
| 351 | MIR377 |
| 352 | SOX10 |
| 353 | LIG4 |
| 354 | ERCC6 |
| 355 | XRCC6 |
| 356 | XRCC5 |
| 357 | NHEJ1 |
| 358 | H2AC18 |
| 359 | EDNRA |
| 360 | EDNRB |
| 361 | MIF |
| 362 | PON1 |
| 363 | NRG1 |
| 364 | ERCC1 |
| 365 | VDAC1 |
| 366 | CALCA |
| 367 | SST |
| 368 | DNAH5 |
| 369 | STRN3 |
| 370 | MIR25 |
| 371 | MRPS17P7 |
| 372 | IDDM8 |
| 373 | CDH13 |
| 374 | IL21 |
| 375 | PCNA |
| 376 | SOD2 |
| 377 | BCL2 |
| 378 | PARP1 |
| 379 | CDKN1A |
| 380 | BAX |
| 381 | CFLAR |
| 382 | MAGEA1 |
| 383 | MALAT1 |
| 384 | MIR155 |
| 385 | IS1 |
| 386 | SERPINE1 |
| 387 | TAT |
| 388 | CALM1 |
| 389 | MSRB2 |
| 390 | FRS2 |
| 391 | LGALS9 |
| 392 | IFNE |
| 393 | MIR9-2 |
| 394 | MIR421 |
| 395 | F2RL1 |
| 396 | MDH2 |
| 397 | FURIN |
| 398 | ACHE |
| 399 | OGG1 |
| 400 | PRSS1 |
| 401 | MX1 |
| 402 | C4A |
| 403 | PDE6A |
| 404 | IL1RAPL1 |
| 405 | RPS27A |
| 406 | DPP4 |
| 407 | ITGAM |
| 408 | MIR20A |
| 409 | CHEK2 |
| 410 | MDM2 |
| 411 | RAD51 |
| 412 | ATR |
| 413 | MYC |
| 414 | TERT |
| 415 | BRCA1 |
| 416 | RAD50 |
| 417 | BLM |
| 418 | FANCD2 |
| 419 | TUBG1 |
| 420 | LIG1 |
| 421 | IRF8 |
| 422 | PML |
| 423 | SMC1A |
| 424 | RPA1 |
| 425 | WRN |
| 426 | KPNA2 |
| 427 | IL23R |
| 428 | AICDA |
| 429 | COX10 |
| 430 | RAD17 |
| 431 | KIR3DL1 |
| 432 | FANCF |
| 433 | TERF1 |
| 434 | MDC1 |
| 435 | FANCB |
| 436 | MRE11 |
| 437 | TERF2 |
| 438 | KIR3DL2 |
| 439 | KIR2DL1 |
| 440 | KIR3DL3 |
| 441 | H2AX |
| 442 | OSGIN2 |
| 443 | TH2LCRR |
| 444 | TH2-LCR |
| 445 | IGF1 |
| 446 | PMS2 |
| 447 | ACADS |
| 448 | IL18 |
| 449 | FOXN1 |
| 450 | CALR |
| 451 | HMGB1 |
| 452 | IL1R1 |
| 453 | TRPS1 |
| 454 | IL1A |
| 455 | FLG |
| 456 | ULBP3 |
| 457 | TCHH |
| 458 | STX17 |
| 459 | ITGA5 |
| 460 | IL4R |
| 461 | OPRL1 |
| 462 | TNFRSF10A |
| 463 | MIA |
| 464 | PNOC |
| 465 | PMCH |
| 466 | PVT1 |
| 467 | PRO2268 |
| 468 | ICOS |
| 469 | CCDC22 |
| 470 | BTK |
| 471 | MCM2 |
| 472 | CR2 |
| 473 | MCM7 |
| 474 | CORO1A |
| 475 | BAD |
| 476 | GAST |
| 477 | GDF15 |
| 478 | MAPT |
| 479 | REN |
| 480 | CD40LG |
| 481 | NR0B1 |
| 482 | TNFSF13B |
| 483 | CD3G |
| 484 | KIF1C |
| 485 | SPG11 |
| 486 | ZFYVE26 |
| 487 | ZFR |
| 488 | SPG21 |
| 489 | SPART |
| 490 | MAPK8 |
| 491 | TRAF2 |
| 492 | BRAF |
| 493 | PPP3CA |
| 494 | PTPN1 |
| 495 | MSH2 |
| 496 | PCYT1A |
| 497 | ESRRA |
| 498 | AHCY |
| 499 | IL2RB |
| 500 | PLCB3 |
| 501 | FLI1 |
| 502 | CYBA |
| 503 | NR1H2 |
| 504 | GALNS |
| 505 | ITGA7 |
| 506 | ACO2 |
| 507 | KAT2A |
| 508 | THBS2 |
| 509 | TNFSF11 |
| 510 | RXRB |
| 511 | PRMT1 |
| 512 | FEN1 |
| 513 | HLA-DPB1 |
| 514 | ITCH |
| 515 | IRF3 |
| 516 | RPS6KA2 |
| 517 | TNFRSF11A |
| 518 | PSMB7 |
| 519 | LGALS3 |
| 520 | NEU1 |
| 521 | DYNC1H1 |
| 522 | HIPK2 |
| 523 | FADS2 |
| 524 | FAP |
| 525 | ABCG1 |
| 526 | TAP2 |
| 527 | TMPRSS6 |
| 528 | SMARCC2 |
| 529 | CDK10 |
| 530 | CDH15 |
| 531 | BRD2 |
| 532 | BCL2L11 |
| 533 | PPIF |
| 534 | NUP62 |
| 535 | PTGES3 |
| 536 | SERPINB6 |
| 537 | PPP2R5C |
| 538 | NTF4 |
| 539 | IRF4 |
| 540 | CBFA2T3 |
| 541 | PNPT1 |
| 542 | RRAS |
| 543 | RHOH |
| 544 | RPL13A |
| 545 | CHRNA9 |
| 546 | BEST1 |
| 547 | CPT1C |
| 548 | CNP |
| 549 | ACSF3 |
| 550 | RNF168 |
| 551 | PARVA |
| 552 | HLA-DPA1 |
| 553 | HLA-DMA |
| 554 | HLA-DMB |
| 555 | KCNK4 |
| 556 | AGPAT1 |
| 557 | CEP57 |
| 558 | ARID5B |
| 559 | INCENP |
| 560 | CD37 |
| 561 | PLIN3 |
| 562 | HLA-DOA |
| 563 | HLA-DOB |
| 564 | HLA-DRB5 |
| 565 | HCRT |
| 566 | GZMH |
| 567 | MYL6 |
| 568 | FARP2 |
| 569 | STK25 |
| 570 | ANAPC1 |
| 571 | CPVL |
| 572 | CARD8 |
| 573 | RAB4A |
| 574 | RAB5C |
| 575 | XK |
| 576 | NEK6 |
| 577 | KCNH4 |
| 578 | RPS11 |
| 579 | UBE2E2 |
| 580 | LIN7B |
| 581 | NKIRAS2 |
| 582 | GJA10 |
| 583 | HLA-DQB2 |
| 584 | KCNA7 |
| 585 | PDS5A |
| 586 | SERPINB9 |
| 587 | SNRNP70 |
| 588 | SESN3 |
| 589 | TNFSF18 |
| 590 | SAFB |
| 591 | TEF |
| 592 | COL19A1 |
| 593 | DNAJC7 |
| 594 | BCL2L12 |
| 595 | ATF6B |
| 596 | BAZ2A |
| 597 | AKAP11 |
| 598 | MICAL2 |
| 599 | RALY |
| 600 | PARP12 |
| 601 | FEM1A |
| 602 | FADS3 |
| 603 | POLR2G |
| 604 | POLR3H |
| 605 | SENP5 |
| 606 | SYNGR2 |
| 607 | SLC37A1 |
| 608 | DHX58 |
| 609 | BAG6 |
| 610 | AHNAK |
| 611 | PTOV1 |
| 612 | USP36 |
| 613 | ZC3H7B |
| 614 | KLHL11 |
| 615 | HRC |
| 616 | PPARGC1B |
| 617 | RTKN2 |
| 618 | RHOU |
| 619 | TTC26 |
| 620 | SARNP |
| 621 | SLC37A3 |
| 622 | SLC6A16 |
| 623 | CYTH4 |
| 624 | AFMID |
| 625 | LUC7L2 |
| 626 | MEI1 |
| 627 | PPFIA3 |
| 628 | PIH1D1 |
| 629 | TRMT112 |
| 630 | ALDH16A1 |
| 631 | ENGASE |
| 632 | CPSF7 |
| 633 | ADPRH |
| 634 | PPP1R14B |
| 635 | N4BP2 |
| 636 | NARS1 |
| 637 | GPR137 |
| 638 | FAM76B |
| 639 | HUS1B |
| 640 | MAPK1IP1L |
| 641 | UMODL1 |
| 642 | TPRG1 |
| 643 | TIGD6 |
| 644 | CHADL |
| 645 | DEF8 |
| 646 | VPS52 |
| 647 | ZC3H10 |
| 648 | ZBTB21 |
| 649 | HMGXB3 |
| 650 | GPSM3 |
| 651 | GPR31 |
| 652 | UBN2 |
| 653 | TRIL |
| 654 | DUS3L |
| 655 | DNAJC14 |
| 656 | CNTD1 |
| 657 | C2CD2 |
| 658 | SCAF1 |
| 659 | SLC45A1 |
| 660 | CGB7 |
| 661 | CYB561A3 |
| 662 | WDR53 |
| 663 | ZBTB39 |
| 664 | PRR12 |
| 665 | CCN4 |
| 666 | DNAAF11 |
| 667 | TMEM109 |
| 668 | DYNLT2B |
| 669 | CFAP36 |
| 670 | ARRDC5 |
| 671 | CBLN3 |
| 672 | HSPB9 |
| 673 | CLHC1 |
| 674 | PABPN1L |
| 675 | ZNF385C |
| 676 | GAGE1 |
| 677 | RETREG3 |
| 678 | PPP4R3B |
| 679 | NBR2 |
| 680 | C19orf73 |
| 681 | MIR7-3HG |
| 682 | SPATA48 |
| 683 | CATSPERZ |
| 684 | MIR378A |
| 685 | TMEM78 |
| 686 | MIR4435-2HG |
| 687 | MIR7-3 |
| 688 | PRORSD1P |
| 689 | CYP21A1P |
| 690 | HLA-DRB6 |
| 691 | HCG23 |
| 692 | SNORD35B |
| 693 | SNORD35A |
| 694 | SNAI3-AS1 |
| 695 | TMEM198B |
| 696 | HLA-DQB1-AS1 |
| 697 | HLA-DRB9 |
| 698 | LOC344967 |
| 699 | LINC01063 |
| 700 | DPP9-AS1 |
| 701 | LNCRNA-IUR |
| 702 | MICD |
| 703 | L3MBTL2-AS1 |
| 704 | RERE-AS1 |
| 705 | LINC02265 |
| 706 | HLA-W |
| 707 | MSH2-OT1 |
| 708 | LINC02341 |
| 709 | LOC101928168 |
| 710 | FTOP1 |
| 711 | RNU6-588P |
| 712 | STK19B |
| 713 | LINC02571 |
| 714 | PHC1P1 |
| 715 | ENSG00000267261 |
| 716 | ENSG00000221083 |
| 717 | ENSG00000249141 |
| 718 | ENSG00000254680 |
| 719 | KRT18P39 |
| 720 | HSPD1P4 |
| 721 | RNU6-1149P |
| 722 | RNU6-742P |
| 723 | CPVL-AS1 |
| 724 | LINC02356 |
| 725 | LINC02624 |
| 726 | LINC02814 |
| 727 | LINC02815 |
| 728 | PPP4R3B-DT |
| 729 | COX6CP2 |
| 730 | RAB5C-AS1 |
| 731 | SLC25A38P1 |
| 732 | ENSG00000257740 |
| 733 | ENSG00000258657 |
| 734 | ENSG00000271780 |
| 735 | ENSG00000203327 |
| 736 | ENSG00000224228 |
| 737 | ENSG00000229971 |
| 738 | ENSG00000235237 |
| 739 | ENSG00000258199 |
| 740 | ENSG00000269807 |
| 741 | ENSG00000205653 |
| 742 | ENSG00000236194 |
| 743 | ENSG00000257449 |
| 744 | ENSG00000258317 |
| 745 | ENSG00000261226 |
| 746 | VDAC1P2 |
| 747 | ENSG00000231467 |
| 748 | HNRNPA1P49 |
| 749 | SNRPEP4 |
| 750 | LINC02498 |
| 751 | ENSG00000285162 |
| 752 | SETP16 |
| 753 | lnc-GCA-5 |
| 754 | ENSG00000205018 |
| 755 | YWHAZP5 |
| 756 | LOC100996598 |
| 757 | LOC105369781 |
| 758 | lnc-C1QTNF6-1 |
| 759 | lnc-MAP3K7-3 |
| 760 | CHORDC1P1 |
| 761 | ENSG00000273890 |
| 762 | HSALNG0079126 |
| 763 | ENSG00000285842 |
| 764 | ENSG00000227370 |
| 765 | lnc-NEDD4L-2 |
| 766 | lnc-BCL2L11-6-001 |
| 767 | lnc-FAM76B-1 |
| 768 | lnc-FGFR1OP-9 |
| 769 | lnc-RTKN2-5 |
| 770 | MN298114-178 |
| 771 | lnc-TBXAS1-1 |
| 772 | lnc-RNASET2-2 |
| 773 | lnc-TOB2-3 |
| 774 | lnc-HLA-DRB5-1-002 |
| 775 | lnc-BCL6-9 |
| 776 | piR-31432-064 |
| 777 | piR-37410 |
| 778 | piR-49399-010 |
| 779 | piR-39701-054 |
| 780 | piR-35530 |
| 781 | piR-36260-057 |
| 782 | piR-32940 |
| 783 | piR-50443-049 |
| 784 | piR-52008-011 |
| 785 | piR-41111-001 |
| 786 | NONHSAG026224.2 |
| 787 | NONHSAG026256.2 |
| 788 | piR-30173 |
| 789 | L13304-029 |
| 790 | HSALNG0031946 |
| 791 | HSALNG0015031 |
| 792 | HSALNG0049408 |
| 793 | HSALNG0049410 |
| 794 | HSALNG0084559 |
| 795 | HSALNG0084560 |
| 796 | HSALNG0015030 |
| 797 | HSALNG0020027 |
| 798 | HSALNG0082719 |
| 799 | HSALNG0084561-001 |
| 800 | HSALNG0049427 |
| 801 | HSALNG0049429 |
| 802 | HSALNG0011289 |
| 803 | HSALNG0011290 |
| 804 | HSALNG0034003 |
| 805 | HSALNG0047287 |
| 806 | HSALNG0047288 |
| 807 | HSALNG0047530 |
| 808 | HSALNG0049409 |
| 809 | HSALNG0049428 |
| 810 | HSALNG0021699 |
| 811 | ENSG00000226233 |
| 812 | LOC105373724 |
| 813 | LOC105373042 |
| 814 | LOC105378119 |
| 815 | LOC105369780 |
| 816 | lnc-NEU1-1 |
| 817 | lnc-NEU1-2 |
| 818 | lnc-SLC37A1-2 |
| 819 | HSALNG0135283 |
| 820 | L13715-021 |
| 821 | lnc-RRAS-5 |
| 822 | MG828730-053 |
| 823 | MN298364 |
| 824 | lnc-KCNH4-1 |
| 825 | lnc-KDM7A-1 |
| 826 | lnc-KDM7A-2 |
| 827 | lnc-HLA-DRB1-8 |
| 828 | HSALNG0113564 |
| 829 | HSALNG0113565 |
| 830 | HSALNG0113566 |
| 831 | HSALNG0133311 |
| 832 | lnc-ERRFI1-4 |
| 833 | HSALNG0121781 |
| 834 | HSALNG0121782 |
| 835 | HSALNG0135284 |
| 836 | lnc-ADGRD2-2 |
| 837 | lnc-DACT2-4 |
| 838 | piR-31534-168 |
| 839 | piR-52524 |
| 840 | piR-53177-042 |
| 841 | RF00017-3868 |
| 842 | RF00017-5028 |
| 843 | RF00017-5281 |
| 844 | RF00017-029 |
| 845 | RF00017-5040 |
| 846 | RF00017-3884 |
| 847 | RF00017-2542 |
| 848 | RF00017-5032 |
| 849 | piR-59409-061 |
| 850 | RF00026-650 |
| 851 | piR-42777 |
| 852 | piR-32286-014 |
| 853 | piR-40154-156 |
| 854 | piR-43107-159 |
| 855 | piR-31534-157 |
| 856 | HSALNG0091478 |
| 857 | KR820244 |
| 858 | HSALNG0045783 |
| 859 | HSALNG0068704 |
| 860 | HSALNG0000637 |
| 861 | FJ868800 |
| 862 | LOC107984667 |
| 863 | lnc-PDE6A-1 |
| 864 | HSALNG0127017 |
| 865 | lnc-RRAS-4 |
| 866 | HSALNG0126958 |
| 867 | HSALNG0126959 |
| 868 | RF00998-075 |
| 869 | RF00017-5543 |
| 870 | RF00017-7459 |
| 871 | piR-49423-221 |
| 872 | piR-33614-192 |
| 873 | piR-35674-157 |
| 874 | HSALNG0014763 |
| 875 | HSALNG0086587 |
| 876 | HSALNG0086584 |
| 877 | HSALNG0096667 |
| 878 | NONHSAG009311.2 |
| 879 | HSALNG0103864 |
| 880 | piR-53338-079 |
| 881 | piR-53431-565 |
| 882 | RF00017-5694 |
| 883 | MMP1 |
| 884 | UGT1A1 |
| 885 | ECHS1 |
| 886 | TGFBR3 |
| 887 | CFB |
| 888 | RGS9 |
| 889 | CHGA |
| 890 | TNFRSF25 |
| 891 | SLC3A2 |
| 892 | XRCC2 |
| 893 | CIDEC |
| 894 | CTSW |
| 895 | GPR174 |
| 896 | NUDT10 |
| 897 | IL2RG |
| 898 | SHTN1 |
| 899 | MIR3940 |
| 900 | AKT1 |
| 901 | IFI30 |
| 902 | GDNF |
| 903 | RPL13 |
| 904 | TRPM2 |
| 905 | IL27RA |
| 906 | FGFR1 |
| 907 | PDGFRB |
| 908 | MAP2K2 |
| 909 | AR |
| 910 | COL1A1 |
| 911 | NR5A1 |
| 912 | GGT1 |
| 913 | ABCG2 |
| 914 | SMAD3 |
| 915 | SMAD4 |
| 916 | ALB |
| 917 | CA2 |
| 918 | PRKDC |
| 919 | IGF2 |
| 920 | KRT18 |
| 921 | GLDC |
| 922 | ELANE |
| 923 | F2 |
| 924 | GLI3 |
| 925 | CACNA1S |
| 926 | ARG1 |
| 927 | CXCR2 |
| 928 | CYP2D6 |
| 929 | CHRM3 |
| 930 | B2M |
| 931 | CYP11B2 |
| 932 | PROS1 |
| 933 | HSPG2 |
| 934 | POLG |
| 935 | TNFAIP3 |
| 936 | TP63 |
| 937 | SPTAN1 |
| 938 | DCN |
| 939 | CYCS |
| 940 | CYP27B1 |
| 941 | MC2R |
| 942 | USP9X |
| 943 | KRT8 |
| 944 | LEP |
| 945 | NDUFS7 |
| 946 | GK |
| 947 | CD79A |
| 948 | TPMT |
| 949 | AMPD1 |
| 950 | ETV6 |
| 951 | CSF3R |
| 952 | KDM6A |
| 953 | AQP5 |
| 954 | TYMP |
| 955 | GHR |
| 956 | HSD3B2 |
| 957 | IGFBP3 |
| 958 | NDUFS1 |
| 959 | STAR |
| 960 | SCN4A |
| 961 | SAG |
| 962 | ARRB2 |
| 963 | EPHX1 |
| 964 | COX4I1 |
| 965 | PRTN3 |
| 966 | DLAT |
| 967 | UQCRC2 |
| 968 | LARS2 |
| 969 | NDUFV2 |
| 970 | NDUFA1 |
| 971 | NDUFS2 |
| 972 | NDUFS4 |
| 973 | GH1 |
| 974 | GRK6 |
| 975 | PMPCA |
| 976 | TP53BP1 |
| 977 | SMAD7 |
| 978 | APOH |
| 979 | AMH |
| 980 | DGUOK |
| 981 | BCS1L |
| 982 | LTF |
| 983 | CS |
| 984 | CXCL12 |
| 985 | WARS2 |
| 986 | GATAD2B |
| 987 | FMR1 |
| 988 | FCGR3B |
| 989 | SLC25A24 |
| 990 | DOCK8 |
| 991 | CD34 |
| 992 | ACAD9 |
| 993 | ARRB1 |
| 994 | SLC17A5 |
| 995 | TSFM |
| 996 | XRCC4 |
| 997 | FTCD |
| 998 | ELAC2 |
| 999 | HAX1 |
| 1000 | MTO1 |
| 1001 | NDUFB8 |
| 1002 | NDUFB3 |
| 1003 | PDSS1 |
| 1004 | SURF1 |
| 1005 | SELE |
| 1006 | SEPSECS |
| 1007 | TRIT1 |
| 1008 | TMC6 |
| 1009 | TFB1M |
| 1010 | TFAM |
| 1011 | COX6B1 |
| 1012 | IGFBP1 |
| 1013 | MBTPS2 |
| 1014 | RARS2 |
| 1015 | PAPPA |
| 1016 | COX15 |
| 1017 | YARS2 |
| 1018 | G6PC3 |
| 1019 | IARS2 |
| 1020 | HBG2 |
| 1021 | FGF7 |
| 1022 | COQ9 |
| 1023 | KAT8 |
| 1024 | GPT |
| 1025 | MRPS22 |
| 1026 | PDSS2 |
| 1027 | PEX3 |
| 1028 | SHBG |
| 1029 | SSB |
| 1030 | SPRED1 |
| 1031 | TRIM21 |
| 1032 | TBX19 |
| 1033 | CDK13 |
| 1034 | ACKR3 |
| 1035 | CXCL13 |
| 1036 | COQ2 |
| 1037 | COX4I2 |
| 1038 | PACS1 |
| 1039 | PEX19 |
| 1040 | COX5B |
| 1041 | COQ6 |
| 1042 | DARS2 |
| 1043 | NDUFAF2 |
| 1044 | IFNA1 |
| 1045 | GK2 |
| 1046 | NDUFB6 |
| 1047 | ADI1 |
| 1048 | MRPL44 |
| 1049 | PDPN |
| 1050 | SIL1 |
| 1051 | TMC8 |
| 1052 | ASGR1 |
| 1053 | ASGR2 |
| 1054 | CSF3 |
| 1055 | PDE6H |
| 1056 | IFT81 |
| 1057 | GTPBP3 |
| 1058 | SERPINA7 |
| 1059 | FBXL4 |
| 1060 | GRK3 |
| 1061 | FIP1L1 |
| 1062 | SHOX2 |
| 1063 | BMP15 |
| 1064 | COX6C |
| 1065 | TRMU |
| 1066 | GOLM1 |
| 1067 | FASTKD2 |
| 1068 | MRRF |
| 1069 | RPS4X |
| 1070 | SHOX |
| 1071 | TMEM70 |
| 1072 | SLC25A17 |
| 1073 | CCN2 |
| 1074 | CCRL2 |
| 1075 | JAKMIP1 |
| 1076 | OSGEPL1 |
| 1077 | PNPLA1 |
| 1078 | PEX11A |
| 1079 | COX7C |
| 1080 | MRPL18 |
| 1081 | SSNA1 |
| 1082 | EPRS1 |
| 1083 | TXNDC17 |
| 1084 | GPR22 |
| 1085 | AHDC1 |
| 1086 | MT-ND2 |
| 1087 | RNPC3 |
| 1088 | SRY |
| 1089 | CCL18 |
| 1090 | ASXL3 |
| 1091 | MTERF1 |
| 1092 | SKOR1 |
| 1093 | ARRDC4 |
| 1094 | EFHC2 |
| 1095 | EFCAB3 |
| 1096 | MFSD12 |
| 1097 | COA3 |
| 1098 | KANSL2 |
| 1099 | MT-ND3 |
| 1100 | OR4L1 |
| 1101 | TSPY1 |
| 1102 | TWNK |
| 1103 | SPPL2C |
| 1104 | TRMT61B |
| 1105 | MT-CYB |
| 1106 | RPS4Y1 |
| 1107 | YRDC |
| 1108 | KANSL1L |
| 1109 | MRM2 |
| 1110 | MT-ATP8 |
| 1111 | LRRC37A3 |
| 1112 | LRRC37A |
| 1113 | CGB3 |
| 1114 | LRRC37A2 |
| 1115 | RO60 |
| 1116 | MT-ND4L |
| 1117 | ARHGAP11B |
| 1118 | ZNRD2 |
| 1119 | XAGE3 |
| 1120 | SRGAP2C |
| 1121 | MIR9-1 |
| 1122 | MT-RNR2 |
| 1123 | IFNG-AS1 |
| 1124 | MAPT-AS1 |
| 1125 | MT-RNR1 |
| 1126 | LINC00426 |
| 1127 | LINC02210 |
| 1128 | CXCR2P1 |
| 1129 | AP4B1-AS1 |
| 1130 | LINC01436 |
| 1131 | MIR663AHG |
| 1132 | MAPT-IT1 |
| 1133 | MT-TG |
| 1134 | LINC02384 |
| 1135 | MT-TM |
| 1136 | MT-TD |
| 1137 | MT-TR |
| 1138 | MIR4315-1 |
| 1139 | TRL-AAG2-3 |
| 1140 | NFKB1 |
| 1141 | NQO1 |
| 1142 | PINK1 |
| 1143 | IL7 |
| 1144 | SGCB |
| 1145 | TTF2 |
| 1146 | MRPL28 |
| 1147 | ASMT |
| 1148 | IDH1 |
| 1149 | FZD4 |
| 1150 | ITGB2 |
| 1151 | RORA |
| 1152 | ITGAL |
| 1153 | MMP2 |
| 1154 | CCL2 |
| 1155 | CD55 |
| 1156 | HNF1A |
| 1157 | HRH2 |
| 1158 | LINC00328 |
| 1159 | RELA |
| 1160 | CASP1 |
| 1161 | TXNRD2 |
| 1162 | CDKN3 |
| 1163 | HPS1 |
| 1164 | MAGEA3 |
| 1165 | PTPN11 |
| 1166 | MAPK14 |
| 1167 | VIM |
| 1168 | SOS1 |
| 1169 | TFRC |
| 1170 | CD36 |
| 1171 | PIK3CG |
| 1172 | ABCC4 |
| 1173 | TXNRD1 |
| 1174 | TGM1 |
| 1175 | IL5 |
| 1176 | VIP |
| 1177 | PRDX4 |
| 1178 | NTS |
| 1179 | PSIP1 |
| 1180 | LGI3 |
| 1181 | IVL |
| 1182 | IL23A |
| 1183 | SOCS4 |
| 1184 | LOC107988030 |
| 1185 | P04798 |
| 1186 | CYP2A6 |
| 1187 | MTOR |
| 1188 | CYP3A4 |
| 1189 | FKBP1A |
| 1190 | ORM1 |
| 1191 | CYP3A5 |
| 1192 | ABCB1 |
| 1193 | ABCA5 |
| 1194 | SLCO1B1 |
| 1195 | PIK3R1 |
| 1196 | DDX39B |
| 1197 | LINC00243 |
| 1198 | ATAT1 |
| 1199 | EGFL8 |
| 1200 | DDR1-DT |
| 1201 | RNF5 |
| 1202 | HCG22 |
| 1203 | TNXB |
| 1204 | SFTA2 |
| 1205 | NOTCH4 |
| 1206 | GTF2H4 |
| 1207 | APOE |
| 1208 | TRIM26 |
| 1209 | MICC |
| 1210 | TRIM31 |
| 1211 | FGFR1OP |
| 1212 | PPT2 |
| 1213 | TRIM15 |
| 1214 | CCHCR1 |
| 1215 | ATP6V1G2-DDX39B |
| 1216 | PPT2-EGFL8 |
| 1217 | LINC01149 |
| 1218 | POU5F1 |
| 1219 | LINC02570 |
| 1220 | MUCL3 |
| 1221 | GABPA |
| 1222 | HT |
| 1223 | HPGDS |
| 1224 | TRBV20OR9-2 |
| 1225 | TBP |
| 1226 | C12orf10 |
| 1227 | COX2 |
| 1228 | VIT |
| 1229 | RTL1 |
| 1230 | NFE2 |
| 1231 | GSTK1 |
| 1232 | IL24 |
| 1233 | GSTM2 |
| 1234 | LRR1 |
| 1235 | SLCO6A1 |
| 1236 | ABO |
| 1237 | IFNA13 |
| 1238 | SOAT1 |
| 1239 | ROS1 |
| 1240 | NLN |
| 1241 | CANX |
| 1242 | KIDINS220 |
| 1243 | ZNF410 |
| 1244 | PRL |
| 1245 | PSMB10 |
| 1246 | USO1 |
| 1247 | MAP2K7 |
| 1248 | PRNP |
| 1249 | CD40 |
| 1250 | OPN4 |
| 1251 | GRAP2 |
| 1252 | PDLIM7 |
| 1253 | PRDX6 |
| 1254 | CBR1 |
| 1255 | CD69 |
| 1256 | AIMP2 |
| 1257 | TLR3 |
| 1258 | CXCL11 |
| 1259 | PRDX2 |
| 1260 | MIR577 |
| 1261 | S100A1 |
| 1262 | RPS6KB1 |
| 1263 | CARD14 |
| 1264 | RANBP2 |
| 1265 | MIR766 |
| 1266 | XRCC1 |
| 1267 | SUMO1 |
| 1268 | TRPC1 |
| 1269 | C4BPA |
| 1270 | TRP-AGG2-5 |
| 1271 | SLC18A2 |
| 1272 | CCDC88A |
| 1273 | ALOX15 |
| 1274 | ALOX12 |
| 1275 | SEC14L2 |
| 1276 | FLNB |
| 1277 | MYCBP2 |
| 1278 | MMRN1 |
| 1279 | FGFR2 |
| 1280 | QPCT |
| 1281 | RNF19A |
| 1282 | PYDC1 |
| 1283 | C1GALT1C1 |
| 1284 | ABR |
| 1285 | PGP |
| 1286 | IFNL3 |
| 1287 | GLI1 |
| 1288 | IL17B |
| 1289 | GATA3 |
| 1290 | POLDIP2 |
| 1291 | IL22RA2 |
| 1292 | NLRP3 |
| 1293 | IL17F |
| 1294 | MTCO2P12 |
| 1295 | SH2B2 |
| 1296 | AHSA1 |
| 1297 | LINC02210-CRHR1 |
| 1298 | NXF1 |
| 1299 | CDX2 |
| 1300 | ABCB6 |
| 1301 | CALR3 |
| 1302 | RBM45 |
| 1303 | ELN |
| 1304 | ATN1 |
| 1305 | DECR1 |
| 1306 | IFNLR1 |
| 1307 | ADRB2 |
| 1308 | RMDN2 |
| 1309 | CTNNB1 |
| 1310 | CRK |
| 1311 | SYBU |
| 1312 | ISYNA1 |
| 1313 | RMDN1 |
| 1314 | SLC22A18 |
| 1315 | OPRM1 |
| 1316 | NAIP |
| 1317 | TRPM1 |
| 1318 | RMDN3 |
| 1319 | PPBP |
| 1320 | PPARG |
| 1321 | PPARD |
| 1322 | NLE1 |
| 1323 | IL20RB |
| 1324 | MBD3 |
| 1325 | PIK3CD |
| 1326 | PIK3CA |
| 1327 | PGD |
| 1328 | KLK3 |
| 1329 | LINC01193 |
| 1330 | IFNB1 |
| 1331 | HSPA1B |
| 1332 | HSPA1A |
| 1333 | HLA-F |
| 1334 | HLA-E |
| 1335 | HLA-DRB3 |
| 1336 | MBD1 |
| 1337 | MAOB |
| 1338 | SH2D1A |
| 1339 | LMNA |
| 1340 | SLC16A12 |
| 1341 | KLRC1 |
| 1342 | ITGAE |
| 1343 | IDO1 |
| 1344 | JAK-2 |
| 1345 | MAO-A |
| 1346 | JAK-3 |
| 1347 | MAO-B |
| 1348 | SYK |
| 1349 | JAK-1 |
| 1350 | PLAUR |
| 1351 | PTK |
| 1352 | NALP1 |
| 1353 | KIAA0926 |
| 1354 | DEFCAP |
| 1355 | CARD7 |
| 1356 | VAMAS1 |
| 1357 | MSPC |
| 1358 | AIADK |
| 1359 | JRRP |

**4. Supplementary Table 4.** **Degree information for PPI.**

| NO | name | Degree |
| --- | --- | --- |
| 1 | AKT1 | 25 |
| 2 | RELA | 24 |
| 3 | STAT3 | 23 |
| 4 | MAPK1 | 20 |
| 5 | MAPK14 | 20 |
| 6 | TNF | 19 |
| 7 | TP53 | 19 |
| 8 | IL6 | 18 |
| 9 | IL10 | 15 |
| 10 | MAPK8 | 15 |
| 11 | NFKB1 | 15 |
| 12 | IL1B | 14 |
| 13 | MYC | 14 |
| 14 | IL2 | 14 |
| 15 | CXCL8 | 13 |
| 16 | IL4 | 13 |
| 17 | ESR1 | 13 |
| 18 | CTNNB1 | 12 |
| 19 | CDKN1A | 11 |
| 20 | CASP3 | 11 |
| 21 | RB1 | 11 |
| 22 | IL1A | 10 |
| 23 | BCL2 | 10 |
| 24 | MMP1 | 9 |
| 25 | CCL2 | 9 |
| 26 | CYCS | 9 |
| 27 | MDM2 | 9 |
| 28 | PPARG | 8 |
| 29 | CXCL10 | 8 |
| 30 | CD40LG | 8 |
| 31 | AR | 8 |
| 32 | MMP2 | 7 |
| 33 | IFNG | 7 |
| 34 | VEGFA | 7 |
| 35 | CDK2 | 7 |
| 36 | MTOR | 7 |
| 37 | NOS2 | 7 |
| 38 | RPS6KB1 | 7 |
| 39 | SYK | 6 |
| 40 | IGFBP3 | 6 |
| 41 | PTGS2 | 6 |
| 42 | BAD | 6 |
| 43 | RXRB | 6 |
| 44 | IGF2 | 5 |
| 45 | F2 | 5 |
| 46 | ICAM1 | 4 |
| 47 | CYP3A4 | 4 |
| 48 | CYP1A2 | 4 |
| 49 | PCNA | 4 |
| 50 | BAX | 4 |
| 51 | VCAM1 | 3 |
| 52 | HMOX1 | 3 |
| 53 | PIK3CG | 3 |
| 54 | CAT | 3 |
| 55 | AHR | 3 |
| 56 | SELE | 2 |
| 57 | HPGDS | 2 |
| 58 | MPO | 2 |
| 59 | ELANE | 2 |
| 60 | MAOB | 2 |
| 61 | HSD3B2 | 2 |
| 62 | UGT1A1 | 2 |
| 63 | PPARD | 2 |
| 64 | CHEK2 | 2 |
| 65 | CASP7 | 2 |
| 66 | EDNRA | 2 |
| 67 | ERBB3 | 2 |
| 68 | PON1 | 1 |
| 69 | PRSS1 | 1 |
| 70 | PNMT | 1 |
| 71 | NFE2L2 | 1 |
| 72 | GSTP1 | 1 |
| 73 | GSTM2 | 1 |
| 74 | GSTM1 | 1 |
| 75 | SERPINE1 | 1 |
| 76 | DPP4 | 1 |
| 77 | XDH | 1 |
| 78 | ESRRA | 1 |
| 79 | PPP3CA | 1 |
| 80 | CXCL11 | 1 |
| 81 | CA2 | 1 |
| 82 | BRAF | 1 |
| 83 | GLI2 | 1 |
| 84 | ADRB2 | 1 |

**5. Supplementary Table 5. 1690 biological processes, cellular components, and molecular functions were enriched by GO.**

| **Category** | **GO** | **Description** |
| --- | --- | --- |
| GO Molecular Functions | GO:0042803 | protein homodimerization activity |
| GO Molecular Functions | GO:0005126 | cytokine receptor binding |
| GO Molecular Functions | GO:0005125 | cytokine activity |
| GO Molecular Functions | GO:0048018 | receptor ligand activity |
| GO Molecular Functions | GO:0030546 | signaling receptor activator activity |
| GO Molecular Functions | GO:0030545 | signaling receptor regulator activity |
| GO Molecular Functions | GO:0140297 | DNA-binding transcription factor binding |
| GO Molecular Functions | GO:0008134 | transcription factor binding |
| GO Molecular Functions | GO:0061629 | RNA polymerase II-specific DNA-binding transcription factor binding |
| GO Molecular Functions | GO:0001221 | transcription coregulator binding |
| GO Molecular Functions | GO:0004879 | nuclear receptor activity |
| GO Molecular Functions | GO:0098531 | ligand-activated transcription factor activity |
| GO Molecular Functions | GO:0019904 | protein domain specific binding |
| GO Molecular Functions | GO:0019902 | phosphatase binding |
| GO Molecular Functions | GO:1901681 | sulfur compound binding |
| GO Molecular Functions | GO:0019207 | kinase regulator activity |
| GO Molecular Functions | GO:0016491 | oxidoreductase activity |
| GO Molecular Functions | GO:0020037 | heme binding |
| GO Molecular Functions | GO:0019903 | protein phosphatase binding |
| GO Molecular Functions | GO:0019900 | kinase binding |
| GO Molecular Functions | GO:0140296 | general transcription initiation factor binding |
| GO Molecular Functions | GO:0046906 | tetrapyrrole binding |
| GO Molecular Functions | GO:0004672 | protein kinase activity |
| GO Molecular Functions | GO:0019901 | protein kinase binding |
| GO Molecular Functions | GO:0033218 | amide binding |
| GO Molecular Functions | GO:0019887 | protein kinase regulator activity |
| GO Molecular Functions | GO:0042277 | peptide binding |
| GO Molecular Functions | GO:0004674 | protein serine/threonine kinase activity |
| GO Molecular Functions | GO:0016773 | phosphotransferase activity, alcohol group as acceptor |
| GO Molecular Functions | GO:0106310 | protein serine kinase activity |
| GO Molecular Functions | GO:0001223 | transcription coactivator binding |
| GO Molecular Functions | GO:0070851 | growth factor receptor binding |
| GO Molecular Functions | GO:0002020 | protease binding |
| GO Molecular Functions | GO:0004712 | protein serine/threonine/tyrosine kinase activity |
| GO Molecular Functions | GO:0019209 | kinase activator activity |
| GO Molecular Functions | GO:0008047 | enzyme activator activity |
| GO Molecular Functions | GO:0016301 | kinase activity |
| GO Molecular Functions | GO:0005496 | steroid binding |
| GO Molecular Functions | GO:0008083 | growth factor activity |
| GO Molecular Functions | GO:0004601 | peroxidase activity |
| GO Molecular Functions | GO:0003707 | nuclear steroid receptor activity |
| GO Molecular Functions | GO:0016684 | oxidoreductase activity, acting on peroxide as acceptor |
| GO Molecular Functions | GO:0008201 | heparin binding |
| GO Molecular Functions | GO:0031406 | carboxylic acid binding |
| GO Molecular Functions | GO:0004364 | glutathione transferase activity |
| GO Molecular Functions | GO:0001091 | RNA polymerase II general transcription initiation factor binding |
| GO Molecular Functions | GO:0003682 | chromatin binding |
| GO Molecular Functions | GO:0017171 | serine hydrolase activity |
| GO Molecular Functions | GO:0030295 | protein kinase activator activity |
| GO Molecular Functions | GO:0031625 | ubiquitin protein ligase binding |
| GO Molecular Functions | GO:0016725 | oxidoreductase activity, acting on CH or CH2 groups |
| GO Molecular Functions | GO:0097718 | disordered domain specific binding |
| GO Molecular Functions | GO:0033293 | monocarboxylic acid binding |
| GO Molecular Functions | GO:0016209 | antioxidant activity |
| GO Molecular Functions | GO:0044389 | ubiquitin-like protein ligase binding |
| GO Molecular Functions | GO:0005178 | integrin binding |
| GO Molecular Functions | GO:0004707 | MAP kinase activity |
| GO Molecular Functions | GO:0005539 | glycosaminoglycan binding |
| GO Molecular Functions | GO:0005504 | fatty acid binding |
| GO Molecular Functions | GO:0004252 | serine-type endopeptidase activity |
| GO Molecular Functions | GO:0001228 | DNA-binding transcription activator activity, RNA polymerase II-specific |
| GO Molecular Functions | GO:0008009 | chemokine activity |
| GO Molecular Functions | GO:0001216 | DNA-binding transcription activator activity |
| GO Molecular Functions | GO:0004497 | monooxygenase activity |
| GO Molecular Functions | GO:0016705 | oxidoreductase activity, acting on paired donors, with incorporation or reduction of molecular oxygen |
| GO Molecular Functions | GO:0045236 | CXCR chemokine receptor binding |
| GO Molecular Functions | GO:0004708 | MAP kinase kinase activity |
| GO Molecular Functions | GO:0008236 | serine-type peptidase activity |
| GO Molecular Functions | GO:0001099 | basal RNA polymerase II transcription machinery binding |
| GO Molecular Functions | GO:0016765 | transferase activity, transferring alkyl or aryl (other than methyl) groups |
| GO Molecular Functions | GO:0001098 | basal transcription machinery binding |
| GO Molecular Functions | GO:0016004 | phospholipase activator activity |
| GO Molecular Functions | GO:0004175 | endopeptidase activity |
| GO Molecular Functions | GO:0060229 | lipase activator activity |
| GO Molecular Functions | GO:0043539 | protein serine/threonine kinase activator activity |
| GO Molecular Functions | GO:0042379 | chemokine receptor binding |
| GO Molecular Functions | GO:0051721 | protein phosphatase 2A binding |
| GO Molecular Functions | GO:0001968 | fibronectin binding |
| GO Molecular Functions | GO:0071889 | 14-3-3 protein binding |
| GO Molecular Functions | GO:0030291 | protein serine/threonine kinase inhibitor activity |
| GO Molecular Functions | GO:0042805 | actinin binding |
| GO Molecular Functions | GO:0051219 | phosphoprotein binding |
| GO Molecular Functions | GO:0030331 | nuclear estrogen receptor binding |
| GO Molecular Functions | GO:0016712 | oxidoreductase activity, acting on paired donors, with incorporation or reduction of molecular oxygen, reduced flavin or flavoprotein as one donor, and incorporation of one atom of oxygen |
| GO Molecular Functions | GO:0001046 | core promoter sequence-specific DNA binding |
| GO Molecular Functions | GO:0050839 | cell adhesion molecule binding |
| GO Molecular Functions | GO:0032813 | tumor necrosis factor receptor superfamily binding |
| GO Molecular Functions | GO:0070888 | E-box binding |
| GO Molecular Functions | GO:0009055 | electron transfer activity |
| GO Molecular Functions | GO:0008233 | peptidase activity |
| GO Molecular Functions | GO:0052689 | carboxylic ester hydrolase activity |
| GO Molecular Functions | GO:0016922 | nuclear receptor binding |
| GO Molecular Functions | GO:0043177 | organic acid binding |
| GO Molecular Functions | GO:0005507 | copper ion binding |
| GO Molecular Functions | GO:0016247 | channel regulator activity |
| GO Molecular Functions | GO:0043621 | protein self-association |
| GO Molecular Functions | GO:0004860 | protein kinase inhibitor activity |
| GO Molecular Functions | GO:0019210 | kinase inhibitor activity |
| GO Molecular Functions | GO:0016788 | hydrolase activity, acting on ester bonds |
| GO Molecular Functions | GO:0001664 | G protein-coupled receptor binding |
| GO Molecular Functions | GO:0001540 | amyloid-beta binding |
| GO Molecular Functions | GO:0051117 | ATPase binding |
| GO Molecular Functions | GO:0008081 | phosphoric diester hydrolase activity |
| GO Molecular Functions | GO:0008022 | protein C-terminus binding |
| GO Molecular Functions | GO:0001227 | DNA-binding transcription repressor activity, RNA polymerase II-specific |
| GO Molecular Functions | GO:0008013 | beta-catenin binding |
| GO Molecular Functions | GO:0001217 | DNA-binding transcription repressor activity |
| GO Molecular Functions | GO:0004620 | phospholipase activity |
| GO Molecular Functions | GO:0051087 | chaperone binding |
| GO Molecular Functions | GO:0047485 | protein N-terminus binding |
| GO Molecular Functions | GO:0046982 | protein heterodimerization activity |
| GO Molecular Functions | GO:0042578 | phosphoric ester hydrolase activity |
| GO Molecular Functions | GO:0016298 | lipase activity |
| GO Molecular Functions | GO:0042826 | histone deacetylase binding |
| GO Molecular Functions | GO:0031072 | heat shock protein binding |
| GO Molecular Functions | GO:0004857 | enzyme inhibitor activity |
| GO Cellular Components | GO:0005667 | transcription regulator complex |
| GO Cellular Components | GO:0034774 | secretory granule lumen |
| GO Cellular Components | GO:0045121 | membrane raft |
| GO Cellular Components | GO:0098857 | membrane microdomain |
| GO Cellular Components | GO:0060205 | cytoplasmic vesicle lumen |
| GO Cellular Components | GO:0031983 | vesicle lumen |
| GO Cellular Components | GO:0031968 | organelle outer membrane |
| GO Cellular Components | GO:0019867 | outer membrane |
| GO Cellular Components | GO:0097136 | Bcl-2 family protein complex |
| GO Cellular Components | GO:0005741 | mitochondrial outer membrane |
| GO Cellular Components | GO:0017053 | transcription repressor complex |
| GO Cellular Components | GO:0005901 | caveola |
| GO Cellular Components | GO:0005740 | mitochondrial envelope |
| GO Cellular Components | GO:0090575 | RNA polymerase II transcription regulator complex |
| GO Cellular Components | GO:0031966 | mitochondrial membrane |
| GO Cellular Components | GO:0044853 | plasma membrane raft |
| GO Cellular Components | GO:0098552 | side of membrane |
| GO Cellular Components | GO:0031012 | extracellular matrix |
| GO Cellular Components | GO:0030312 | external encapsulating structure |
| GO Cellular Components | GO:0030139 | endocytic vesicle |
| GO Cellular Components | GO:0045171 | intercellular bridge |
| GO Cellular Components | GO:0031970 | organelle envelope lumen |
| GO Cellular Components | GO:0062023 | collagen-containing extracellular matrix |
| GO Cellular Components | GO:0016605 | PML body |
| GO Cellular Components | GO:0009897 | external side of plasma membrane |
| GO Cellular Components | GO:0005788 | endoplasmic reticulum lumen |
| GO Cellular Components | GO:0045177 | apical part of cell |
| GO Cellular Components | GO:0005764 | lysosome |
| GO Cellular Components | GO:0000323 | lytic vacuole |
| GO Cellular Components | GO:0000307 | cyclin-dependent protein kinase holoenzyme complex |
| GO Cellular Components | GO:1904813 | ficolin-1-rich granule lumen |
| GO Cellular Components | GO:0045335 | phagocytic vesicle |
| GO Cellular Components | GO:0072562 | blood microparticle |
| GO Cellular Components | GO:0019897 | extrinsic component of plasma membrane |
| GO Cellular Components | GO:0043073 | germ cell nucleus |
| GO Cellular Components | GO:0031093 | platelet alpha granule lumen |
| GO Cellular Components | GO:0098978 | glutamatergic synapse |
| GO Cellular Components | GO:0048471 | perinuclear region of cytoplasm |
| GO Cellular Components | GO:0005925 | focal adhesion |
| GO Cellular Components | GO:0019898 | extrinsic component of membrane |
| GO Cellular Components | GO:0005758 | mitochondrial intermembrane space |
| GO Cellular Components | GO:0030055 | cell-substrate junction |
| GO Cellular Components | GO:0101002 | ficolin-1-rich granule |
| GO Cellular Components | GO:0031091 | platelet alpha granule |
| GO Cellular Components | GO:0035578 | azurophil granule lumen |
| GO Cellular Components | GO:0032993 | protein-DNA complex |
| GO Cellular Components | GO:1902554 | serine/threonine protein kinase complex |
| GO Cellular Components | GO:0043235 | receptor complex |
| GO Cellular Components | GO:0042383 | sarcolemma |
| GO Biological Processes | GO:0032496 | response to lipopolysaccharide |
| GO Biological Processes | GO:0002237 | response to molecule of bacterial origin |
| GO Biological Processes | GO:0009410 | response to xenobiotic stimulus |
| GO Biological Processes | GO:0009725 | response to hormone |
| GO Biological Processes | GO:0009617 | response to bacterium |
| GO Biological Processes | GO:1901699 | cellular response to nitrogen compound |
| GO Biological Processes | GO:0071396 | cellular response to lipid |
| GO Biological Processes | GO:0006954 | inflammatory response |
| GO Biological Processes | GO:0071417 | cellular response to organonitrogen compound |
| GO Biological Processes | GO:0009314 | response to radiation |
| GO Biological Processes | GO:0036293 | response to decreased oxygen levels |
| GO Biological Processes | GO:0031667 | response to nutrient levels |
| GO Biological Processes | GO:0070482 | response to oxygen levels |
| GO Biological Processes | GO:0001666 | response to hypoxia |
| GO Biological Processes | GO:0009991 | response to extracellular stimulus |
| GO Biological Processes | GO:0071216 | cellular response to biotic stimulus |
| GO Biological Processes | GO:0010035 | response to inorganic substance |
| GO Biological Processes | GO:0009411 | response to UV |
| GO Biological Processes | GO:0022407 | regulation of cell-cell adhesion |
| GO Biological Processes | GO:0008285 | negative regulation of cell population proliferation |
| GO Biological Processes | GO:0050865 | regulation of cell activation |
| GO Biological Processes | GO:1901652 | response to peptide |
| GO Biological Processes | GO:0071222 | cellular response to lipopolysaccharide |
| GO Biological Processes | GO:0048732 | gland development |
| GO Biological Processes | GO:0071219 | cellular response to molecule of bacterial origin |
| GO Biological Processes | GO:0022409 | positive regulation of cell-cell adhesion |
| GO Biological Processes | GO:1903039 | positive regulation of leukocyte cell-cell adhesion |
| GO Biological Processes | GO:1903037 | regulation of leukocyte cell-cell adhesion |
| GO Biological Processes | GO:2001233 | regulation of apoptotic signaling pathway |
| GO Biological Processes | GO:2001234 | negative regulation of apoptotic signaling pathway |
| GO Biological Processes | GO:0071407 | cellular response to organic cyclic compound |
| GO Biological Processes | GO:0002694 | regulation of leukocyte activation |
| GO Biological Processes | GO:0000302 | response to reactive oxygen species |
| GO Biological Processes | GO:0045785 | positive regulation of cell adhesion |
| GO Biological Processes | GO:1904035 | regulation of epithelial cell apoptotic process |
| GO Biological Processes | GO:0030335 | positive regulation of cell migration |
| GO Biological Processes | GO:0051249 | regulation of lymphocyte activation |
| GO Biological Processes | GO:0071466 | cellular response to xenobiotic stimulus |
| GO Biological Processes | GO:0035094 | response to nicotine |
| GO Biological Processes | GO:2000147 | positive regulation of cell motility |
| GO Biological Processes | GO:0040017 | positive regulation of locomotion |
| GO Biological Processes | GO:0042327 | positive regulation of phosphorylation |
| GO Biological Processes | GO:0050727 | regulation of inflammatory response |
| GO Biological Processes | GO:0001775 | cell activation |
| GO Biological Processes | GO:0043068 | positive regulation of programmed cell death |
| GO Biological Processes | GO:0006979 | response to oxidative stress |
| GO Biological Processes | GO:0009416 | response to light stimulus |
| GO Biological Processes | GO:0002696 | positive regulation of leukocyte activation |
| GO Biological Processes | GO:0050863 | regulation of T cell activation |
| GO Biological Processes | GO:0070663 | regulation of leukocyte proliferation |
| GO Biological Processes | GO:0051251 | positive regulation of lymphocyte activation |
| GO Biological Processes | GO:0050867 | positive regulation of cell activation |
| GO Biological Processes | GO:0048545 | response to steroid hormone |
| GO Biological Processes | GO:0010942 | positive regulation of cell death |
| GO Biological Processes | GO:0001934 | positive regulation of protein phosphorylation |
| GO Biological Processes | GO:1901653 | cellular response to peptide |
| GO Biological Processes | GO:0097190 | apoptotic signaling pathway |
| GO Biological Processes | GO:2001237 | negative regulation of extrinsic apoptotic signaling pathway |
| GO Biological Processes | GO:0009611 | response to wounding |
| GO Biological Processes | GO:0048660 | regulation of smooth muscle cell proliferation |
| GO Biological Processes | GO:0062197 | cellular response to chemical stress |
| GO Biological Processes | GO:0002685 | regulation of leukocyte migration |
| GO Biological Processes | GO:0051090 | regulation of DNA-binding transcription factor activity |
| GO Biological Processes | GO:0008283 | cell population proliferation |
| GO Biological Processes | GO:0034762 | regulation of transmembrane transport |
| GO Biological Processes | GO:0002521 | leukocyte differentiation |
| GO Biological Processes | GO:0071345 | cellular response to cytokine stimulus |
| GO Biological Processes | GO:0050670 | regulation of lymphocyte proliferation |
| GO Biological Processes | GO:0080135 | regulation of cellular response to stress |
| GO Biological Processes | GO:0032944 | regulation of mononuclear cell proliferation |
| GO Biological Processes | GO:0050870 | positive regulation of T cell activation |
| GO Biological Processes | GO:0010038 | response to metal ion |
| GO Biological Processes | GO:0045321 | leukocyte activation |
| GO Biological Processes | GO:0034612 | response to tumor necrosis factor |
| GO Biological Processes | GO:0051347 | positive regulation of transferase activity |
| GO Biological Processes | GO:0104004 | cellular response to environmental stimulus |
| GO Biological Processes | GO:0071214 | cellular response to abiotic stimulus |
| GO Biological Processes | GO:0043408 | regulation of MAPK cascade |
| GO Biological Processes | GO:0010817 | regulation of hormone levels |
| GO Biological Processes | GO:0043065 | positive regulation of apoptotic process |
| GO Biological Processes | GO:0009636 | response to toxic substance |
| GO Biological Processes | GO:0045596 | negative regulation of cell differentiation |
| GO Biological Processes | GO:0051046 | regulation of secretion |
| GO Biological Processes | GO:0051384 | response to glucocorticoid |
| GO Biological Processes | GO:0036294 | cellular response to decreased oxygen levels |
| GO Biological Processes | GO:0001819 | positive regulation of cytokine production |
| GO Biological Processes | GO:0034764 | positive regulation of transmembrane transport |
| GO Biological Processes | GO:1903530 | regulation of secretion by cell |
| GO Biological Processes | GO:0030097 | hemopoiesis |
| GO Biological Processes | GO:0007584 | response to nutrient |
| GO Biological Processes | GO:1904019 | epithelial cell apoptotic process |
| GO Biological Processes | GO:0050900 | leukocyte migration |
| GO Biological Processes | GO:2000628 | regulation of miRNA metabolic process |
| GO Biological Processes | GO:0010720 | positive regulation of cell development |
| GO Biological Processes | GO:0031347 | regulation of defense response |
| GO Biological Processes | GO:0043434 | response to peptide hormone |
| GO Biological Processes | GO:2001236 | regulation of extrinsic apoptotic signaling pathway |
| GO Biological Processes | GO:0071453 | cellular response to oxygen levels |
| GO Biological Processes | GO:0032355 | response to estradiol |
| GO Biological Processes | GO:1903131 | mononuclear cell differentiation |
| GO Biological Processes | GO:0046649 | lymphocyte activation |
| GO Biological Processes | GO:0031960 | response to corticosteroid |
| GO Biological Processes | GO:0070665 | positive regulation of leukocyte proliferation |
| GO Biological Processes | GO:0051091 | positive regulation of DNA-binding transcription factor activity |
| GO Biological Processes | GO:0030162 | regulation of proteolysis |
| GO Biological Processes | GO:0050708 | regulation of protein secretion |
| GO Biological Processes | GO:0034599 | cellular response to oxidative stress |
| GO Biological Processes | GO:0051345 | positive regulation of hydrolase activity |
| GO Biological Processes | GO:1903706 | regulation of hemopoiesis |
| GO Biological Processes | GO:1902893 | regulation of miRNA transcription |
| GO Biological Processes | GO:0071456 | cellular response to hypoxia |
| GO Biological Processes | GO:0050678 | regulation of epithelial cell proliferation |
| GO Biological Processes | GO:0043410 | positive regulation of MAPK cascade |
| GO Biological Processes | GO:0035239 | tube morphogenesis |
| GO Biological Processes | GO:0032870 | cellular response to hormone stimulus |
| GO Biological Processes | GO:0042129 | regulation of T cell proliferation |
| GO Biological Processes | GO:0010212 | response to ionizing radiation |
| GO Biological Processes | GO:0042542 | response to hydrogen peroxide |
| GO Biological Processes | GO:0050671 | positive regulation of lymphocyte proliferation |
| GO Biological Processes | GO:0051223 | regulation of protein transport |
| GO Biological Processes | GO:0042110 | T cell activation |
| GO Biological Processes | GO:0070848 | response to growth factor |
| GO Biological Processes | GO:0032102 | negative regulation of response to external stimulus |
| GO Biological Processes | GO:0032946 | positive regulation of mononuclear cell proliferation |
| GO Biological Processes | GO:0097305 | response to alcohol |
| GO Biological Processes | GO:0032103 | positive regulation of response to external stimulus |
| GO Biological Processes | GO:1902105 | regulation of leukocyte differentiation |
| GO Biological Processes | GO:0070201 | regulation of establishment of protein localization |
| GO Biological Processes | GO:0097192 | extrinsic apoptotic signaling pathway in absence of ligand |
| GO Biological Processes | GO:0038034 | signal transduction in absence of ligand |
| GO Biological Processes | GO:0062012 | regulation of small molecule metabolic process |
| GO Biological Processes | GO:0042060 | wound healing |
| GO Biological Processes | GO:0007507 | heart development |
| GO Biological Processes | GO:0010332 | response to gamma radiation |
| GO Biological Processes | GO:0009612 | response to mechanical stimulus |
| GO Biological Processes | GO:0050673 | epithelial cell proliferation |
| GO Biological Processes | GO:0030098 | lymphocyte differentiation |
| GO Biological Processes | GO:2000630 | positive regulation of miRNA metabolic process |
| GO Biological Processes | GO:0006935 | chemotaxis |
| GO Biological Processes | GO:0042330 | taxis |
| GO Biological Processes | GO:0001568 | blood vessel development |
| GO Biological Processes | GO:0006468 | protein phosphorylation |
| GO Biological Processes | GO:0032757 | positive regulation of interleukin-8 production |
| GO Biological Processes | GO:0019221 | cytokine-mediated signaling pathway |
| GO Biological Processes | GO:0040011 | locomotion |
| GO Biological Processes | GO:0001944 | vasculature development |
| GO Biological Processes | GO:0043549 | regulation of kinase activity |
| GO Biological Processes | GO:0051222 | positive regulation of protein transport |
| GO Biological Processes | GO:0032677 | regulation of interleukin-8 production |
| GO Biological Processes | GO:0042102 | positive regulation of T cell proliferation |
| GO Biological Processes | GO:0002366 | leukocyte activation involved in immune response |
| GO Biological Processes | GO:0090087 | regulation of peptide transport |
| GO Biological Processes | GO:0002687 | positive regulation of leukocyte migration |
| GO Biological Processes | GO:0002252 | immune effector process |
| GO Biological Processes | GO:0071887 | leukocyte apoptotic process |
| GO Biological Processes | GO:0002263 | cell activation involved in immune response |
| GO Biological Processes | GO:1904951 | positive regulation of establishment of protein localization |
| GO Biological Processes | GO:0060326 | cell chemotaxis |
| GO Biological Processes | GO:1901214 | regulation of neuron death |
| GO Biological Processes | GO:0097191 | extrinsic apoptotic signaling pathway |
| GO Biological Processes | GO:0008015 | blood circulation |
| GO Biological Processes | GO:0002526 | acute inflammatory response |
| GO Biological Processes | GO:0000165 | MAPK cascade |
| GO Biological Processes | GO:0003013 | circulatory system process |
| GO Biological Processes | GO:0050796 | regulation of insulin secretion |
| GO Biological Processes | GO:1902895 | positive regulation of miRNA transcription |
| GO Biological Processes | GO:0044057 | regulation of system process |
| GO Biological Processes | GO:0045765 | regulation of angiogenesis |
| GO Biological Processes | GO:0043269 | regulation of monoatomic ion transport |
| GO Biological Processes | GO:0040013 | negative regulation of locomotion |
| GO Biological Processes | GO:0033674 | positive regulation of kinase activity |
| GO Biological Processes | GO:0034614 | cellular response to reactive oxygen species |
| GO Biological Processes | GO:1901342 | regulation of vasculature development |
| GO Biological Processes | GO:0007568 | aging |
| GO Biological Processes | GO:0010721 | negative regulation of cell development |
| GO Biological Processes | GO:0007346 | regulation of mitotic cell cycle |
| GO Biological Processes | GO:0045936 | negative regulation of phosphate metabolic process |
| GO Biological Processes | GO:0010563 | negative regulation of phosphorus metabolic process |
| GO Biological Processes | GO:0045471 | response to ethanol |
| GO Biological Processes | GO:0045862 | positive regulation of proteolysis |
| GO Biological Processes | GO:0040008 | regulation of growth |
| GO Biological Processes | GO:0071900 | regulation of protein serine/threonine kinase activity |
| GO Biological Processes | GO:0045859 | regulation of protein kinase activity |
| GO Biological Processes | GO:0050864 | regulation of B cell activation |
| GO Biological Processes | GO:0030336 | negative regulation of cell migration |
| GO Biological Processes | GO:0018105 | peptidyl-serine phosphorylation |
| GO Biological Processes | GO:0050731 | positive regulation of peptidyl-tyrosine phosphorylation |
| GO Biological Processes | GO:0045786 | negative regulation of cell cycle |
| GO Biological Processes | GO:0071478 | cellular response to radiation |
| GO Biological Processes | GO:2000351 | regulation of endothelial cell apoptotic process |
| GO Biological Processes | GO:0034644 | cellular response to UV |
| GO Biological Processes | GO:0071375 | cellular response to peptide hormone stimulus |
| GO Biological Processes | GO:0071356 | cellular response to tumor necrosis factor |
| GO Biological Processes | GO:0050728 | negative regulation of inflammatory response |
| GO Biological Processes | GO:1903829 | positive regulation of protein localization |
| GO Biological Processes | GO:0046883 | regulation of hormone secretion |
| GO Biological Processes | GO:1904036 | negative regulation of epithelial cell apoptotic process |
| GO Biological Processes | GO:2000146 | negative regulation of cell motility |
| GO Biological Processes | GO:0051047 | positive regulation of secretion |
| GO Biological Processes | GO:0042063 | gliogenesis |
| GO Biological Processes | GO:0090276 | regulation of peptide hormone secretion |
| GO Biological Processes | GO:0071496 | cellular response to external stimulus |
| GO Biological Processes | GO:0032642 | regulation of chemokine production |
| GO Biological Processes | GO:0002791 | regulation of peptide secretion |
| GO Biological Processes | GO:0048661 | positive regulation of smooth muscle cell proliferation |
| GO Biological Processes | GO:0001525 | angiogenesis |
| GO Biological Processes | GO:0002683 | negative regulation of immune system process |
| GO Biological Processes | GO:0071363 | cellular response to growth factor stimulus |
| GO Biological Processes | GO:0062013 | positive regulation of small molecule metabolic process |
| GO Biological Processes | GO:0018209 | peptidyl-serine modification |
| GO Biological Processes | GO:0030595 | leukocyte chemotaxis |
| GO Biological Processes | GO:0009895 | negative regulation of catabolic process |
| GO Biological Processes | GO:0052548 | regulation of endopeptidase activity |
| GO Biological Processes | GO:2000377 | regulation of reactive oxygen species metabolic process |
| GO Biological Processes | GO:0043281 | regulation of cysteine-type endopeptidase activity involved in apoptotic process |
| GO Biological Processes | GO:0007169 | transmembrane receptor protein tyrosine kinase signaling pathway |
| GO Biological Processes | GO:0031400 | negative regulation of protein modification process |
| GO Biological Processes | GO:0031099 | regeneration |
| GO Biological Processes | GO:0010638 | positive regulation of organelle organization |
| GO Biological Processes | GO:2000379 | positive regulation of reactive oxygen species metabolic process |
| GO Biological Processes | GO:0019932 | second-messenger-mediated signaling |
| GO Biological Processes | GO:0048514 | blood vessel morphogenesis |
| GO Biological Processes | GO:0010959 | regulation of metal ion transport |
| GO Biological Processes | GO:0019216 | regulation of lipid metabolic process |
| GO Biological Processes | GO:0050878 | regulation of body fluid levels |
| GO Biological Processes | GO:0048511 | rhythmic process |
| GO Biological Processes | GO:0010506 | regulation of autophagy |
| GO Biological Processes | GO:0045637 | regulation of myeloid cell differentiation |
| GO Biological Processes | GO:0008630 | intrinsic apoptotic signaling pathway in response to DNA damage |
| GO Biological Processes | GO:0097193 | intrinsic apoptotic signaling pathway |
| GO Biological Processes | GO:0030522 | intracellular receptor signaling pathway |
| GO Biological Processes | GO:0043523 | regulation of neuron apoptotic process |
| GO Biological Processes | GO:0007167 | enzyme-linked receptor protein signaling pathway |
| GO Biological Processes | GO:0002360 | T cell lineage commitment |
| GO Biological Processes | GO:1903532 | positive regulation of secretion by cell |
| GO Biological Processes | GO:0010827 | regulation of glucose transmembrane transport |
| GO Biological Processes | GO:0045860 | positive regulation of protein kinase activity |
| GO Biological Processes | GO:0050679 | positive regulation of epithelial cell proliferation |
| GO Biological Processes | GO:0045930 | negative regulation of mitotic cell cycle |
| GO Biological Processes | GO:0051052 | regulation of DNA metabolic process |
| GO Biological Processes | GO:0071482 | cellular response to light stimulus |
| GO Biological Processes | GO:0051098 | regulation of binding |
| GO Biological Processes | GO:0002673 | regulation of acute inflammatory response |
| GO Biological Processes | GO:0043270 | positive regulation of monoatomic ion transport |
| GO Biological Processes | GO:0030217 | T cell differentiation |
| GO Biological Processes | GO:0051960 | regulation of nervous system development |
| GO Biological Processes | GO:0019725 | cellular homeostasis |
| GO Biological Processes | GO:0002761 | regulation of myeloid leukocyte differentiation |
| GO Biological Processes | GO:2000116 | regulation of cysteine-type endopeptidase activity |
| GO Biological Processes | GO:0051054 | positive regulation of DNA metabolic process |
| GO Biological Processes | GO:0034767 | positive regulation of monoatomic ion transmembrane transport |
| GO Biological Processes | GO:0050767 | regulation of neurogenesis |
| GO Biological Processes | GO:0050871 | positive regulation of B cell activation |
| GO Biological Processes | GO:0042326 | negative regulation of phosphorylation |
| GO Biological Processes | GO:1902532 | negative regulation of intracellular signal transduction |
| GO Biological Processes | GO:0002697 | regulation of immune effector process |
| GO Biological Processes | GO:0030879 | mammary gland development |
| GO Biological Processes | GO:0002688 | regulation of leukocyte chemotaxis |
| GO Biological Processes | GO:2000134 | negative regulation of G1/S transition of mitotic cell cycle |
| GO Biological Processes | GO:0030183 | B cell differentiation |
| GO Biological Processes | GO:0002703 | regulation of leukocyte mediated immunity |
| GO Biological Processes | GO:1902107 | positive regulation of leukocyte differentiation |
| GO Biological Processes | GO:1903708 | positive regulation of hemopoiesis |
| GO Biological Processes | GO:0051051 | negative regulation of transport |
| GO Biological Processes | GO:0001936 | regulation of endothelial cell proliferation |
| GO Biological Processes | GO:0046686 | response to cadmium ion |
| GO Biological Processes | GO:0045165 | cell fate commitment |
| GO Biological Processes | GO:0007159 | leukocyte cell-cell adhesion |
| GO Biological Processes | GO:0009743 | response to carbohydrate |
| GO Biological Processes | GO:0043467 | regulation of generation of precursor metabolites and energy |
| GO Biological Processes | GO:0019722 | calcium-mediated signaling |
| GO Biological Processes | GO:0001101 | response to acid chemical |
| GO Biological Processes | GO:0050730 | regulation of peptidyl-tyrosine phosphorylation |
| GO Biological Processes | GO:0031334 | positive regulation of protein-containing complex assembly |
| GO Biological Processes | GO:0097529 | myeloid leukocyte migration |
| GO Biological Processes | GO:0007610 | behavior |
| GO Biological Processes | GO:0033135 | regulation of peptidyl-serine phosphorylation |
| GO Biological Processes | GO:0051924 | regulation of calcium ion transport |
| GO Biological Processes | GO:0050778 | positive regulation of immune response |
| GO Biological Processes | GO:0052547 | regulation of peptidase activity |
| GO Biological Processes | GO:0001933 | negative regulation of protein phosphorylation |
| GO Biological Processes | GO:1902807 | negative regulation of cell cycle G1/S phase transition |
| GO Biological Processes | GO:0032755 | positive regulation of interleukin-6 production |
| GO Biological Processes | GO:0042692 | muscle cell differentiation |
| GO Biological Processes | GO:0006974 | cellular response to DNA damage stimulus |
| GO Biological Processes | GO:0022612 | gland morphogenesis |
| GO Biological Processes | GO:0045834 | positive regulation of lipid metabolic process |
| GO Biological Processes | GO:0001503 | ossification |
| GO Biological Processes | GO:0070227 | lymphocyte apoptotic process |
| GO Biological Processes | GO:0031331 | positive regulation of cellular catabolic process |
| GO Biological Processes | GO:0032655 | regulation of interleukin-12 production |
| GO Biological Processes | GO:0048771 | tissue remodeling |
| GO Biological Processes | GO:0010564 | regulation of cell cycle process |
| GO Biological Processes | GO:0030888 | regulation of B cell proliferation |
| GO Biological Processes | GO:0031348 | negative regulation of defense response |
| GO Biological Processes | GO:0032868 | response to insulin |
| GO Biological Processes | GO:0014013 | regulation of gliogenesis |
| GO Biological Processes | GO:0033138 | positive regulation of peptidyl-serine phosphorylation |
| GO Biological Processes | GO:0007249 | I-kappaB kinase/NF-kappaB signaling |
| GO Biological Processes | GO:0045670 | regulation of osteoclast differentiation |
| GO Biological Processes | GO:0006959 | humoral immune response |
| GO Biological Processes | GO:0042742 | defense response to bacterium |
| GO Biological Processes | GO:0034284 | response to monosaccharide |
| GO Biological Processes | GO:0048871 | multicellular organismal homeostasis |
| GO Biological Processes | GO:0010632 | regulation of epithelial cell migration |
| GO Biological Processes | GO:0043369 | CD4-positive or CD8-positive, alpha-beta T cell lineage commitment |
| GO Biological Processes | GO:1901615 | organic hydroxy compound metabolic process |
| GO Biological Processes | GO:0061138 | morphogenesis of a branching epithelium |
| GO Biological Processes | GO:0046632 | alpha-beta T cell differentiation |
| GO Biological Processes | GO:0030856 | regulation of epithelial cell differentiation |
| GO Biological Processes | GO:0097237 | cellular response to toxic substance |
| GO Biological Processes | GO:0071241 | cellular response to inorganic substance |
| GO Biological Processes | GO:0050920 | regulation of chemotaxis |
| GO Biological Processes | GO:0060558 | regulation of calcidiol 1-monooxygenase activity |
| GO Biological Processes | GO:0010950 | positive regulation of endopeptidase activity |
| GO Biological Processes | GO:0031663 | lipopolysaccharide-mediated signaling pathway |
| GO Biological Processes | GO:1904037 | positive regulation of epithelial cell apoptotic process |
| GO Biological Processes | GO:0048662 | negative regulation of smooth muscle cell proliferation |
| GO Biological Processes | GO:2001242 | regulation of intrinsic apoptotic signaling pathway |
| GO Biological Processes | GO:0043200 | response to amino acid |
| GO Biological Processes | GO:0001763 | morphogenesis of a branching structure |
| GO Biological Processes | GO:0045058 | T cell selection |
| GO Biological Processes | GO:0098754 | detoxification |
| GO Biological Processes | GO:0032675 | regulation of interleukin-6 production |
| GO Biological Processes | GO:0006805 | xenobiotic metabolic process |
| GO Biological Processes | GO:0006351 | DNA-templated transcription |
| GO Biological Processes | GO:0043280 | positive regulation of cysteine-type endopeptidase activity involved in apoptotic process |
| GO Biological Processes | GO:0097659 | nucleic acid-templated transcription |
| GO Biological Processes | GO:0051048 | negative regulation of secretion |
| GO Biological Processes | GO:0032409 | regulation of transporter activity |
| GO Biological Processes | GO:1905952 | regulation of lipid localization |
| GO Biological Processes | GO:0033273 | response to vitamin |
| GO Biological Processes | GO:0071316 | cellular response to nicotine |
| GO Biological Processes | GO:0006109 | regulation of carbohydrate metabolic process |
| GO Biological Processes | GO:0030593 | neutrophil chemotaxis |
| GO Biological Processes | GO:0048638 | regulation of developmental growth |
| GO Biological Processes | GO:0032774 | RNA biosynthetic process |
| GO Biological Processes | GO:1904018 | positive regulation of vasculature development |
| GO Biological Processes | GO:0045766 | positive regulation of angiogenesis |
| GO Biological Processes | GO:0010952 | positive regulation of peptidase activity |
| GO Biological Processes | GO:2000045 | regulation of G1/S transition of mitotic cell cycle |
| GO Biological Processes | GO:0008217 | regulation of blood pressure |
| GO Biological Processes | GO:0008202 | steroid metabolic process |
| GO Biological Processes | GO:0002700 | regulation of production of molecular mediator of immune response |
| GO Biological Processes | GO:0042113 | B cell activation |
| GO Biological Processes | GO:0045927 | positive regulation of growth |
| GO Biological Processes | GO:0000082 | G1/S transition of mitotic cell cycle |
| GO Biological Processes | GO:0010001 | glial cell differentiation |
| GO Biological Processes | GO:0044843 | cell cycle G1/S phase transition |
| GO Biological Processes | GO:0006366 | transcription by RNA polymerase II |
| GO Biological Processes | GO:1901863 | positive regulation of muscle tissue development |
| GO Biological Processes | GO:0060627 | regulation of vesicle-mediated transport |
| GO Biological Processes | GO:0071902 | positive regulation of protein serine/threonine kinase activity |
| GO Biological Processes | GO:0071621 | granulocyte chemotaxis |
| GO Biological Processes | GO:0046889 | positive regulation of lipid biosynthetic process |
| GO Biological Processes | GO:0010507 | negative regulation of autophagy |
| GO Biological Processes | GO:0048145 | regulation of fibroblast proliferation |
| GO Biological Processes | GO:0031652 | positive regulation of heat generation |
| GO Biological Processes | GO:0002699 | positive regulation of immune effector process |
| GO Biological Processes | GO:0048754 | branching morphogenesis of an epithelial tube |
| GO Biological Processes | GO:0001649 | osteoblast differentiation |
| GO Biological Processes | GO:1904996 | positive regulation of leukocyte adhesion to vascular endothelial cell |
| GO Biological Processes | GO:0009896 | positive regulation of catabolic process |
| GO Biological Processes | GO:2001056 | positive regulation of cysteine-type endopeptidase activity |
| GO Biological Processes | GO:1990266 | neutrophil migration |
| GO Biological Processes | GO:1901216 | positive regulation of neuron death |
| GO Biological Processes | GO:0042178 | xenobiotic catabolic process |
| GO Biological Processes | GO:0002009 | morphogenesis of an epithelium |
| GO Biological Processes | GO:0042176 | regulation of protein catabolic process |
| GO Biological Processes | GO:0042135 | neurotransmitter catabolic process |
| GO Biological Processes | GO:0050714 | positive regulation of protein secretion |
| GO Biological Processes | GO:0043086 | negative regulation of catalytic activity |
| GO Biological Processes | GO:0051146 | striated muscle cell differentiation |
| GO Biological Processes | GO:0002690 | positive regulation of leukocyte chemotaxis |
| GO Biological Processes | GO:0051402 | neuron apoptotic process |
| GO Biological Processes | GO:0003012 | muscle system process |
| GO Biological Processes | GO:0048729 | tissue morphogenesis |
| GO Biological Processes | GO:0050768 | negative regulation of neurogenesis |
| GO Biological Processes | GO:1902806 | regulation of cell cycle G1/S phase transition |
| GO Biological Processes | GO:0042759 | long-chain fatty acid biosynthetic process |
| GO Biological Processes | GO:0070231 | T cell apoptotic process |
| GO Biological Processes | GO:0002675 | positive regulation of acute inflammatory response |
| GO Biological Processes | GO:0032768 | regulation of monooxygenase activity |
| GO Biological Processes | GO:0043279 | response to alkaloid |
| GO Biological Processes | GO:0009408 | response to heat |
| GO Biological Processes | GO:0001890 | placenta development |
| GO Biological Processes | GO:1904064 | positive regulation of cation transmembrane transport |
| GO Biological Processes | GO:0046631 | alpha-beta T cell activation |
| GO Biological Processes | GO:0060559 | positive regulation of calcidiol 1-monooxygenase activity |
| GO Biological Processes | GO:0050729 | positive regulation of inflammatory response |
| GO Biological Processes | GO:0044819 | mitotic G1/S transition checkpoint signaling |
| GO Biological Processes | GO:0031571 | mitotic G1 DNA damage checkpoint signaling |
| GO Biological Processes | GO:1901215 | negative regulation of neuron death |
| GO Biological Processes | GO:0097530 | granulocyte migration |
| GO Biological Processes | GO:0034765 | regulation of monoatomic ion transmembrane transport |
| GO Biological Processes | GO:0051961 | negative regulation of nervous system development |
| GO Biological Processes | GO:0043368 | positive T cell selection |
| GO Biological Processes | GO:0097421 | liver regeneration |
| GO Biological Processes | GO:0044772 | mitotic cell cycle phase transition |
| GO Biological Processes | GO:0016241 | regulation of macroautophagy |
| GO Biological Processes | GO:0050804 | modulation of chemical synaptic transmission |
| GO Biological Processes | GO:0099177 | regulation of trans-synaptic signaling |
| GO Biological Processes | GO:0072593 | reactive oxygen species metabolic process |
| GO Biological Processes | GO:1990748 | cellular detoxification |
| GO Biological Processes | GO:0045428 | regulation of nitric oxide biosynthetic process |
| GO Biological Processes | GO:0071276 | cellular response to cadmium ion |
| GO Biological Processes | GO:0031650 | regulation of heat generation |
| GO Biological Processes | GO:0070997 | neuron death |
| GO Biological Processes | GO:1901796 | regulation of signal transduction by p53 class mediator |
| GO Biological Processes | GO:0051100 | negative regulation of binding |
| GO Biological Processes | GO:0050769 | positive regulation of neurogenesis |
| GO Biological Processes | GO:0032787 | monocarboxylic acid metabolic process |
| GO Biological Processes | GO:0007162 | negative regulation of cell adhesion |
| GO Biological Processes | GO:1905330 | regulation of morphogenesis of an epithelium |
| GO Biological Processes | GO:0080164 | regulation of nitric oxide metabolic process |
| GO Biological Processes | GO:0044089 | positive regulation of cellular component biogenesis |
| GO Biological Processes | GO:0061061 | muscle structure development |
| GO Biological Processes | GO:0034250 | positive regulation of amide metabolic process |
| GO Biological Processes | GO:1990776 | response to angiotensin |
| GO Biological Processes | GO:0044770 | cell cycle phase transition |
| GO Biological Processes | GO:0031100 | animal organ regeneration |
| GO Biological Processes | GO:0043254 | regulation of protein-containing complex assembly |
| GO Biological Processes | GO:1903034 | regulation of response to wounding |
| GO Biological Processes | GO:0009266 | response to temperature stimulus |
| GO Biological Processes | GO:0007565 | female pregnancy |
| GO Biological Processes | GO:1901988 | negative regulation of cell cycle phase transition |
| GO Biological Processes | GO:1901099 | negative regulation of signal transduction in absence of ligand |
| GO Biological Processes | GO:2001240 | negative regulation of extrinsic apoptotic signaling pathway in absence of ligand |
| GO Biological Processes | GO:2000352 | negative regulation of endothelial cell apoptotic process |
| GO Biological Processes | GO:0006006 | glucose metabolic process |
| GO Biological Processes | GO:0006633 | fatty acid biosynthetic process |
| GO Biological Processes | GO:0001558 | regulation of cell growth |
| GO Biological Processes | GO:0010634 | positive regulation of epithelial cell migration |
| GO Biological Processes | GO:0008203 | cholesterol metabolic process |
| GO Biological Processes | GO:0010469 | regulation of signaling receptor activity |
| GO Biological Processes | GO:0014823 | response to activity |
| GO Biological Processes | GO:0032722 | positive regulation of chemokine production |
| GO Biological Processes | GO:0031330 | negative regulation of cellular catabolic process |
| GO Biological Processes | GO:1901991 | negative regulation of mitotic cell cycle phase transition |
| GO Biological Processes | GO:0002718 | regulation of cytokine production involved in immune response |
| GO Biological Processes | GO:0031668 | cellular response to extracellular stimulus |
| GO Biological Processes | GO:0090257 | regulation of muscle system process |
| GO Biological Processes | GO:2000278 | regulation of DNA biosynthetic process |
| GO Biological Processes | GO:0046890 | regulation of lipid biosynthetic process |
| GO Biological Processes | GO:0051403 | stress-activated MAPK cascade |
| GO Biological Processes | GO:0046651 | lymphocyte proliferation |
| GO Biological Processes | GO:0071675 | regulation of mononuclear cell migration |
| GO Biological Processes | GO:1903522 | regulation of blood circulation |
| GO Biological Processes | GO:0097398 | cellular response to interleukin-17 |
| GO Biological Processes | GO:0097396 | response to interleukin-17 |
| GO Biological Processes | GO:0071901 | negative regulation of protein serine/threonine kinase activity |
| GO Biological Processes | GO:0032680 | regulation of tumor necrosis factor production |
| GO Biological Processes | GO:1903169 | regulation of calcium ion transmembrane transport |
| GO Biological Processes | GO:0043405 | regulation of MAP kinase activity |
| GO Biological Processes | GO:0032411 | positive regulation of transporter activity |
| GO Biological Processes | GO:0032890 | regulation of organic acid transport |
| GO Biological Processes | GO:1904062 | regulation of monoatomic cation transmembrane transport |
| GO Biological Processes | GO:0055082 | cellular chemical homeostasis |
| GO Biological Processes | GO:0046427 | positive regulation of receptor signaling pathway via JAK-STAT |
| GO Biological Processes | GO:0060251 | regulation of glial cell proliferation |
| GO Biological Processes | GO:0031281 | positive regulation of cyclase activity |
| GO Biological Processes | GO:0032943 | mononuclear cell proliferation |
| GO Biological Processes | GO:0006970 | response to osmotic stress |
| GO Biological Processes | GO:0002637 | regulation of immunoglobulin production |
| GO Biological Processes | GO:0006919 | activation of cysteine-type endopeptidase activity involved in apoptotic process |
| GO Biological Processes | GO:1903555 | regulation of tumor necrosis factor superfamily cytokine production |
| GO Biological Processes | GO:1902652 | secondary alcohol metabolic process |
| GO Biological Processes | GO:0003018 | vascular process in circulatory system |
| GO Biological Processes | GO:0090559 | regulation of membrane permeability |
| GO Biological Processes | GO:0046394 | carboxylic acid biosynthetic process |
| GO Biological Processes | GO:0050777 | negative regulation of immune response |
| GO Biological Processes | GO:0090399 | replicative senescence |
| GO Biological Processes | GO:0045579 | positive regulation of B cell differentiation |
| GO Biological Processes | GO:0006953 | acute-phase response |
| GO Biological Processes | GO:1904994 | regulation of leukocyte adhesion to vascular endothelial cell |
| GO Biological Processes | GO:0044703 | multi-organism reproductive process |
| GO Biological Processes | GO:0048568 | embryonic organ development |
| GO Biological Processes | GO:0045787 | positive regulation of cell cycle |
| GO Biological Processes | GO:0051928 | positive regulation of calcium ion transport |
| GO Biological Processes | GO:1900015 | regulation of cytokine production involved in inflammatory response |
| GO Biological Processes | GO:0002286 | T cell activation involved in immune response |
| GO Biological Processes | GO:0031098 | stress-activated protein kinase signaling cascade |
| GO Biological Processes | GO:0032872 | regulation of stress-activated MAPK cascade |
| GO Biological Processes | GO:0045621 | positive regulation of lymphocyte differentiation |
| GO Biological Processes | GO:0016053 | organic acid biosynthetic process |
| GO Biological Processes | GO:0051281 | positive regulation of release of sequestered calcium ion into cytosol |
| GO Biological Processes | GO:0045123 | cellular extravasation |
| GO Biological Processes | GO:0150077 | regulation of neuroinflammatory response |
| GO Biological Processes | GO:0001889 | liver development |
| GO Biological Processes | GO:0070302 | regulation of stress-activated protein kinase signaling cascade |
| GO Biological Processes | GO:0030099 | myeloid cell differentiation |
| GO Biological Processes | GO:0016125 | sterol metabolic process |
| GO Biological Processes | GO:0070555 | response to interleukin-1 |
| GO Biological Processes | GO:0060965 | negative regulation of miRNA-mediated gene silencing |
| GO Biological Processes | GO:0002363 | alpha-beta T cell lineage commitment |
| GO Biological Processes | GO:0045429 | positive regulation of nitric oxide biosynthetic process |
| GO Biological Processes | GO:0032733 | positive regulation of interleukin-10 production |
| GO Biological Processes | GO:0002285 | lymphocyte activation involved in immune response |
| GO Biological Processes | GO:0071248 | cellular response to metal ion |
| GO Biological Processes | GO:0061008 | hepaticobiliary system development |
| GO Biological Processes | GO:0033555 | multicellular organismal response to stress |
| GO Biological Processes | GO:0044706 | multi-multicellular organism process |
| GO Biological Processes | GO:0045840 | positive regulation of mitotic nuclear division |
| GO Biological Processes | GO:0032735 | positive regulation of interleukin-12 production |
| GO Biological Processes | GO:1904894 | positive regulation of receptor signaling pathway via STAT |
| GO Biological Processes | GO:0051348 | negative regulation of transferase activity |
| GO Biological Processes | GO:0051962 | positive regulation of nervous system development |
| GO Biological Processes | GO:0001818 | negative regulation of cytokine production |
| GO Biological Processes | GO:1904407 | positive regulation of nitric oxide metabolic process |
| GO Biological Processes | GO:0043620 | regulation of DNA-templated transcription in response to stress |
| GO Biological Processes | GO:0060967 | negative regulation of gene silencing by RNA |
| GO Biological Processes | GO:1900369 | negative regulation of post-transcriptional gene silencing by RNA |
| GO Biological Processes | GO:0060149 | negative regulation of post-transcriptional gene silencing |
| GO Biological Processes | GO:0006977 | DNA damage response, signal transduction by p53 class mediator resulting in cell cycle arrest |
| GO Biological Processes | GO:1901654 | response to ketone |
| GO Biological Processes | GO:0098586 | cellular response to virus |
| GO Biological Processes | GO:0042594 | response to starvation |
| GO Biological Processes | GO:0002573 | myeloid leukocyte differentiation |
| GO Biological Processes | GO:0061419 | positive regulation of transcription from RNA polymerase II promoter in response to hypoxia |
| GO Biological Processes | GO:0034349 | glial cell apoptotic process |
| GO Biological Processes | GO:0034103 | regulation of tissue remodeling |
| GO Biological Processes | GO:0010948 | negative regulation of cell cycle process |
| GO Biological Processes | GO:0022898 | regulation of transmembrane transporter activity |
| GO Biological Processes | GO:0035296 | regulation of tube diameter |
| GO Biological Processes | GO:0097746 | blood vessel diameter maintenance |
| GO Biological Processes | GO:2001239 | regulation of extrinsic apoptotic signaling pathway in absence of ligand |
| GO Biological Processes | GO:1901861 | regulation of muscle tissue development |
| GO Biological Processes | GO:1901522 | positive regulation of transcription from RNA polymerase II promoter involved in cellular response to chemical stimulus |
| GO Biological Processes | GO:0035150 | regulation of tube size |
| GO Biological Processes | GO:0050921 | positive regulation of chemotaxis |
| GO Biological Processes | GO:1904892 | regulation of receptor signaling pathway via STAT |
| GO Biological Processes | GO:0045619 | regulation of lymphocyte differentiation |
| GO Biological Processes | GO:0070661 | leukocyte proliferation |
| GO Biological Processes | GO:0055123 | digestive system development |
| GO Biological Processes | GO:0045601 | regulation of endothelial cell differentiation |
| GO Biological Processes | GO:0001822 | kidney development |
| GO Biological Processes | GO:0045598 | regulation of fat cell differentiation |
| GO Biological Processes | GO:0014910 | regulation of smooth muscle cell migration |
| GO Biological Processes | GO:0048538 | thymus development |
| GO Biological Processes | GO:0031349 | positive regulation of defense response |
| GO Biological Processes | GO:0006469 | negative regulation of protein kinase activity |
| GO Biological Processes | GO:1902004 | positive regulation of amyloid-beta formation |
| GO Biological Processes | GO:0048643 | positive regulation of skeletal muscle tissue development |
| GO Biological Processes | GO:0000278 | mitotic cell cycle |
| GO Biological Processes | GO:0035924 | cellular response to vascular endothelial growth factor stimulus |
| GO Biological Processes | GO:0071383 | cellular response to steroid hormone stimulus |
| GO Biological Processes | GO:0031669 | cellular response to nutrient levels |
| GO Biological Processes | GO:1904427 | positive regulation of calcium ion transmembrane transport |
| GO Biological Processes | GO:0008585 | female gonad development |
| GO Biological Processes | GO:1900542 | regulation of purine nucleotide metabolic process |
| GO Biological Processes | GO:0030855 | epithelial cell differentiation |
| GO Biological Processes | GO:0072001 | renal system development |
| GO Biological Processes | GO:0019748 | secondary metabolic process |
| GO Biological Processes | GO:0043491 | protein kinase B signaling |
| GO Biological Processes | GO:0002902 | regulation of B cell apoptotic process |
| GO Biological Processes | GO:0010224 | response to UV-B |
| GO Biological Processes | GO:0006140 | regulation of nucleotide metabolic process |
| GO Biological Processes | GO:0043535 | regulation of blood vessel endothelial cell migration |
| GO Biological Processes | GO:0010675 | regulation of cellular carbohydrate metabolic process |
| GO Biological Processes | GO:0032368 | regulation of lipid transport |
| GO Biological Processes | GO:0030003 | cellular monoatomic cation homeostasis |
| GO Biological Processes | GO:1904705 | regulation of vascular associated smooth muscle cell proliferation |
| GO Biological Processes | GO:0001894 | tissue homeostasis |
| GO Biological Processes | GO:0060249 | anatomical structure homeostasis |
| GO Biological Processes | GO:0045087 | innate immune response |
| GO Biological Processes | GO:0043367 | CD4-positive, alpha-beta T cell differentiation |
| GO Biological Processes | GO:0060688 | regulation of morphogenesis of a branching structure |
| GO Biological Processes | GO:0009746 | response to hexose |
| GO Biological Processes | GO:0048534 | hematopoietic or lymphoid organ development |
| GO Biological Processes | GO:0032091 | negative regulation of protein binding |
| GO Biological Processes | GO:0045807 | positive regulation of endocytosis |
| GO Biological Processes | GO:0043524 | negative regulation of neuron apoptotic process |
| GO Biological Processes | GO:0051712 | positive regulation of killing of cells of another organism |
| GO Biological Processes | GO:0040007 | growth |
| GO Biological Processes | GO:0048589 | developmental growth |
| GO Biological Processes | GO:1903531 | negative regulation of secretion by cell |
| GO Biological Processes | GO:0060562 | epithelial tube morphogenesis |
| GO Biological Processes | GO:0046545 | development of primary female sexual characteristics |
| GO Biological Processes | GO:0030168 | platelet activation |
| GO Biological Processes | GO:0010623 | programmed cell death involved in cell development |
| GO Biological Processes | GO:1902894 | negative regulation of miRNA transcription |
| GO Biological Processes | GO:0001836 | release of cytochrome c from mitochondria |
| GO Biological Processes | GO:0006091 | generation of precursor metabolites and energy |
| GO Biological Processes | GO:0019318 | hexose metabolic process |
| GO Biological Processes | GO:0070372 | regulation of ERK1 and ERK2 cascade |
| GO Biological Processes | GO:1903047 | mitotic cell cycle process |
| GO Biological Processes | GO:0006873 | cellular monoatomic ion homeostasis |
| GO Biological Processes | GO:0048260 | positive regulation of receptor-mediated endocytosis |
| GO Biological Processes | GO:0001541 | ovarian follicle development |
| GO Biological Processes | GO:0010594 | regulation of endothelial cell migration |
| GO Biological Processes | GO:0007276 | gamete generation |
| GO Biological Processes | GO:0051092 | positive regulation of NF-kappaB transcription factor activity |
| GO Biological Processes | GO:0006631 | fatty acid metabolic process |
| GO Biological Processes | GO:0072330 | monocarboxylic acid biosynthetic process |
| GO Biological Processes | GO:1904645 | response to amyloid-beta |
| GO Biological Processes | GO:0030857 | negative regulation of epithelial cell differentiation |
| GO Biological Processes | GO:2000629 | negative regulation of miRNA metabolic process |
| GO Biological Processes | GO:0002764 | immune response-regulating signaling pathway |
| GO Biological Processes | GO:0002520 | immune system development |
| GO Biological Processes | GO:0043525 | positive regulation of neuron apoptotic process |
| GO Biological Processes | GO:0033559 | unsaturated fatty acid metabolic process |
| GO Biological Processes | GO:0071347 | cellular response to interleukin-1 |
| GO Biological Processes | GO:0033673 | negative regulation of kinase activity |
| GO Biological Processes | GO:0045639 | positive regulation of myeloid cell differentiation |
| GO Biological Processes | GO:0071346 | cellular response to type II interferon |
| GO Biological Processes | GO:0048639 | positive regulation of developmental growth |
| GO Biological Processes | GO:0051043 | regulation of membrane protein ectodomain proteolysis |
| GO Biological Processes | GO:0000077 | DNA damage checkpoint signaling |
| GO Biological Processes | GO:1901376 | organic heteropentacyclic compound metabolic process |
| GO Biological Processes | GO:0031649 | heat generation |
| GO Biological Processes | GO:0043385 | mycotoxin metabolic process |
| GO Biological Processes | GO:0010573 | vascular endothelial growth factor production |
| GO Biological Processes | GO:0060556 | regulation of vitamin D biosynthetic process |
| GO Biological Processes | GO:0031622 | positive regulation of fever generation |
| GO Biological Processes | GO:0046222 | aflatoxin metabolic process |
| GO Biological Processes | GO:0042391 | regulation of membrane potential |
| GO Biological Processes | GO:0050853 | B cell receptor signaling pathway |
| GO Biological Processes | GO:1902993 | positive regulation of amyloid precursor protein catabolic process |
| GO Biological Processes | GO:0009267 | cellular response to starvation |
| GO Biological Processes | GO:0051785 | positive regulation of nuclear division |
| GO Biological Processes | GO:0070228 | regulation of lymphocyte apoptotic process |
| GO Biological Processes | GO:0046324 | regulation of glucose import |
| GO Biological Processes | GO:0001676 | long-chain fatty acid metabolic process |
| GO Biological Processes | GO:0045833 | negative regulation of lipid metabolic process |
| GO Biological Processes | GO:0035994 | response to muscle stretch |
| GO Biological Processes | GO:0050995 | negative regulation of lipid catabolic process |
| GO Biological Processes | GO:0002444 | myeloid leukocyte mediated immunity |
| GO Biological Processes | GO:0044283 | small molecule biosynthetic process |
| GO Biological Processes | GO:0031341 | regulation of cell killing |
| GO Biological Processes | GO:0051341 | regulation of oxidoreductase activity |
| GO Biological Processes | GO:0008637 | apoptotic mitochondrial changes |
| GO Biological Processes | GO:0090398 | cellular senescence |
| GO Biological Processes | GO:0060135 | maternal process involved in female pregnancy |
| GO Biological Processes | GO:0031279 | regulation of cyclase activity |
| GO Biological Processes | GO:0005996 | monosaccharide metabolic process |
| GO Biological Processes | GO:0050680 | negative regulation of epithelial cell proliferation |
| GO Biological Processes | GO:0042133 | neurotransmitter metabolic process |
| GO Biological Processes | GO:1904385 | cellular response to angiotensin |
| GO Biological Processes | GO:0048641 | regulation of skeletal muscle tissue development |
| GO Biological Processes | GO:0032682 | negative regulation of chemokine production |
| GO Biological Processes | GO:0046902 | regulation of mitochondrial membrane permeability |
| GO Biological Processes | GO:0046660 | female sex differentiation |
| GO Biological Processes | GO:0032414 | positive regulation of ion transmembrane transporter activity |
| GO Biological Processes | GO:0043122 | regulation of I-kappaB kinase/NF-kappaB signaling |
| GO Biological Processes | GO:0045580 | regulation of T cell differentiation |
| GO Biological Processes | GO:0006606 | protein import into nucleus |
| GO Biological Processes | GO:0031570 | DNA integrity checkpoint signaling |
| GO Biological Processes | GO:0048872 | homeostasis of number of cells |
| GO Biological Processes | GO:0051709 | regulation of killing of cells of another organism |
| GO Biological Processes | GO:0090400 | stress-induced premature senescence |
| GO Biological Processes | GO:0051121 | hepoxilin metabolic process |
| GO Biological Processes | GO:0051122 | hepoxilin biosynthetic process |
| GO Biological Processes | GO:0009820 | alkaloid metabolic process |
| GO Biological Processes | GO:0006690 | icosanoid metabolic process |
| GO Biological Processes | GO:0001938 | positive regulation of endothelial cell proliferation |
| GO Biological Processes | GO:1901987 | regulation of cell cycle phase transition |
| GO Biological Processes | GO:0090068 | positive regulation of cell cycle process |
| GO Biological Processes | GO:0032107 | regulation of response to nutrient levels |
| GO Biological Processes | GO:0032104 | regulation of response to extracellular stimulus |
| GO Biological Processes | GO:0035710 | CD4-positive, alpha-beta T cell activation |
| GO Biological Processes | GO:1905953 | negative regulation of lipid localization |
| GO Biological Processes | GO:0045582 | positive regulation of T cell differentiation |
| GO Biological Processes | GO:0015980 | energy derivation by oxidation of organic compounds |
| GO Biological Processes | GO:1901990 | regulation of mitotic cell cycle phase transition |
| GO Biological Processes | GO:0048863 | stem cell differentiation |
| GO Biological Processes | GO:0002706 | regulation of lymphocyte mediated immunity |
| GO Biological Processes | GO:0043406 | positive regulation of MAP kinase activity |
| GO Biological Processes | GO:0048708 | astrocyte differentiation |
| GO Biological Processes | GO:0048009 | insulin-like growth factor receptor signaling pathway |
| GO Biological Processes | GO:0010575 | positive regulation of vascular endothelial growth factor production |
| GO Biological Processes | GO:0032653 | regulation of interleukin-10 production |
| GO Biological Processes | GO:0030278 | regulation of ossification |
| GO Biological Processes | GO:0051170 | import into nucleus |
| GO Biological Processes | GO:0009615 | response to virus |
| GO Biological Processes | GO:0051101 | regulation of DNA binding |
| GO Biological Processes | GO:0042531 | positive regulation of tyrosine phosphorylation of STAT protein |
| GO Biological Processes | GO:0018107 | peptidyl-threonine phosphorylation |
| GO Biological Processes | GO:0002822 | regulation of adaptive immune response based on somatic recombination of immune receptors built from immunoglobulin superfamily domains |
| GO Biological Processes | GO:0071480 | cellular response to gamma radiation |
| GO Biological Processes | GO:0034504 | protein localization to nucleus |
| GO Biological Processes | GO:0002698 | negative regulation of immune effector process |
| GO Biological Processes | GO:1903799 | negative regulation of miRNA maturation |
| GO Biological Processes | GO:0031620 | regulation of fever generation |
| GO Biological Processes | GO:0007548 | sex differentiation |
| GO Biological Processes | GO:0070301 | cellular response to hydrogen peroxide |
| GO Biological Processes | GO:0071354 | cellular response to interleukin-6 |
| GO Biological Processes | GO:0032412 | regulation of monoatomic ion transmembrane transporter activity |
| GO Biological Processes | GO:0034341 | response to type II interferon |
| GO Biological Processes | GO:0010876 | lipid localization |
| GO Biological Processes | GO:0045600 | positive regulation of fat cell differentiation |
| GO Biological Processes | GO:0032770 | positive regulation of monooxygenase activity |
| GO Biological Processes | GO:0072678 | T cell migration |
| GO Biological Processes | GO:0009755 | hormone-mediated signaling pathway |
| GO Biological Processes | GO:0042698 | ovulation cycle |
| GO Biological Processes | GO:1900076 | regulation of cellular response to insulin stimulus |
| GO Biological Processes | GO:0048015 | phosphatidylinositol-mediated signaling |
| GO Biological Processes | GO:0071260 | cellular response to mechanical stimulus |
| GO Biological Processes | GO:0048147 | negative regulation of fibroblast proliferation |
| GO Biological Processes | GO:2000191 | regulation of fatty acid transport |
| GO Biological Processes | GO:0001659 | temperature homeostasis |
| GO Biological Processes | GO:0055080 | monoatomic cation homeostasis |
| GO Biological Processes | GO:0044346 | fibroblast apoptotic process |
| GO Biological Processes | GO:0002676 | regulation of chronic inflammatory response |
| GO Biological Processes | GO:0010595 | positive regulation of endothelial cell migration |
| GO Biological Processes | GO:0032652 | regulation of interleukin-1 production |
| GO Biological Processes | GO:2000573 | positive regulation of DNA biosynthetic process |
| GO Biological Processes | GO:0045661 | regulation of myoblast differentiation |
| GO Biological Processes | GO:0061844 | antimicrobial humoral immune response mediated by antimicrobial peptide |
| GO Biological Processes | GO:0071479 | cellular response to ionizing radiation |
| GO Biological Processes | GO:0048608 | reproductive structure development |
| GO Biological Processes | GO:0048565 | digestive tract development |
| GO Biological Processes | GO:0002262 | myeloid cell homeostasis |
| GO Biological Processes | GO:0002819 | regulation of adaptive immune response |
| GO Biological Processes | GO:0022408 | negative regulation of cell-cell adhesion |
| GO Biological Processes | GO:0070741 | response to interleukin-6 |
| GO Biological Processes | GO:0016242 | negative regulation of macroautophagy |
| GO Biological Processes | GO:0043500 | muscle adaptation |
| GO Biological Processes | GO:0060964 | regulation of miRNA-mediated gene silencing |
| GO Biological Processes | GO:0034405 | response to fluid shear stress |
| GO Biological Processes | GO:0045577 | regulation of B cell differentiation |
| GO Biological Processes | GO:0043536 | positive regulation of blood vessel endothelial cell migration |
| GO Biological Processes | GO:0043401 | steroid hormone mediated signaling pathway |
| GO Biological Processes | GO:2000241 | regulation of reproductive process |
| GO Biological Processes | GO:0042445 | hormone metabolic process |
| GO Biological Processes | GO:0001701 | in utero embryonic development |
| GO Biological Processes | GO:0002757 | immune response-activating signaling pathway |
| GO Biological Processes | GO:0002702 | positive regulation of production of molecular mediator of immune response |
| GO Biological Processes | GO:0061458 | reproductive system development |
| GO Biological Processes | GO:1903036 | positive regulation of response to wounding |
| GO Biological Processes | GO:0050801 | monoatomic ion homeostasis |
| GO Biological Processes | GO:1902253 | regulation of intrinsic apoptotic signaling pathway by p53 class mediator |
| GO Biological Processes | GO:0018210 | peptidyl-threonine modification |
| GO Biological Processes | GO:0048017 | inositol lipid-mediated signaling |
| GO Biological Processes | GO:0046425 | regulation of receptor signaling pathway via JAK-STAT |
| GO Biological Processes | GO:0042770 | signal transduction in response to DNA damage |
| GO Biological Processes | GO:0061041 | regulation of wound healing |
| GO Biological Processes | GO:0046887 | positive regulation of hormone secretion |
| GO Biological Processes | GO:0061418 | regulation of transcription from RNA polymerase II promoter in response to hypoxia |
| GO Biological Processes | GO:0035821 | modulation of process of another organism |
| GO Biological Processes | GO:0070242 | thymocyte apoptotic process |
| GO Biological Processes | GO:0072577 | endothelial cell apoptotic process |
| GO Biological Processes | GO:0071695 | anatomical structure maturation |
| GO Biological Processes | GO:1905144 | response to acetylcholine |
| GO Biological Processes | GO:1900368 | regulation of post-transcriptional gene silencing by RNA |
| GO Biological Processes | GO:0046903 | secretion |
| GO Biological Processes | GO:1903201 | regulation of oxidative stress-induced cell death |
| GO Biological Processes | GO:0045913 | positive regulation of carbohydrate metabolic process |
| GO Biological Processes | GO:0044773 | mitotic DNA damage checkpoint signaling |
| GO Biological Processes | GO:0060147 | regulation of post-transcriptional gene silencing |
| GO Biological Processes | GO:0002437 | inflammatory response to antigenic stimulus |
| GO Biological Processes | GO:0071312 | cellular response to alkaloid |
| GO Biological Processes | GO:0061900 | glial cell activation |
| GO Biological Processes | GO:0098926 | postsynaptic signal transduction |
| GO Biological Processes | GO:0043009 | chordate embryonic development |
| GO Biological Processes | GO:0014074 | response to purine-containing compound |
| GO Biological Processes | GO:0051279 | regulation of release of sequestered calcium ion into cytosol |
| GO Biological Processes | GO:0042509 | regulation of tyrosine phosphorylation of STAT protein |
| GO Biological Processes | GO:0032024 | positive regulation of insulin secretion |
| GO Biological Processes | GO:0050848 | regulation of calcium-mediated signaling |
| GO Biological Processes | GO:0008406 | gonad development |
| GO Biological Processes | GO:0060966 | regulation of gene silencing by RNA |
| GO Biological Processes | GO:0043618 | regulation of transcription from RNA polymerase II promoter in response to stress |
| GO Biological Processes | GO:0098609 | cell-cell adhesion |
| GO Biological Processes | GO:0002544 | chronic inflammatory response |
| GO Biological Processes | GO:0006978 | DNA damage response, signal transduction by p53 class mediator resulting in transcription of p21 class mediator |
| GO Biological Processes | GO:0030656 | regulation of vitamin metabolic process |
| GO Biological Processes | GO:0002295 | T-helper cell lineage commitment |
| GO Biological Processes | GO:0014068 | positive regulation of phosphatidylinositol 3-kinase signaling |
| GO Biological Processes | GO:0044774 | mitotic DNA integrity checkpoint signaling |
| GO Biological Processes | GO:0006693 | prostaglandin metabolic process |
| GO Biological Processes | GO:0042093 | T-helper cell differentiation |
| GO Biological Processes | GO:0042743 | hydrogen peroxide metabolic process |
| GO Biological Processes | GO:0006692 | prostanoid metabolic process |
| GO Biological Processes | GO:0002294 | CD4-positive, alpha-beta T cell differentiation involved in immune response |
| GO Biological Processes | GO:0045137 | development of primary sexual characteristics |
| GO Biological Processes | GO:0050866 | negative regulation of cell activation |
| GO Biological Processes | GO:0120161 | regulation of cold-induced thermogenesis |
| GO Biological Processes | GO:0045667 | regulation of osteoblast differentiation |
| GO Biological Processes | GO:0010660 | regulation of muscle cell apoptotic process |
| GO Biological Processes | GO:0002705 | positive regulation of leukocyte mediated immunity |
| GO Biological Processes | GO:0002293 | alpha-beta T cell differentiation involved in immune response |
| GO Biological Processes | GO:0050850 | positive regulation of calcium-mediated signaling |
| GO Biological Processes | GO:0002287 | alpha-beta T cell activation involved in immune response |
| GO Biological Processes | GO:0030330 | DNA damage response, signal transduction by p53 class mediator |
| GO Biological Processes | GO:0071392 | cellular response to estradiol stimulus |
| GO Biological Processes | GO:0043029 | T cell homeostasis |
| GO Biological Processes | GO:0070884 | regulation of calcineurin-NFAT signaling cascade |
| GO Biological Processes | GO:1900182 | positive regulation of protein localization to nucleus |
| GO Biological Processes | GO:0006875 | cellular metal ion homeostasis |
| GO Biological Processes | GO:0009792 | embryo development ending in birth or egg hatching |
| GO Biological Processes | GO:0050806 | positive regulation of synaptic transmission |
| GO Biological Processes | GO:0031644 | regulation of nervous system process |
| GO Biological Processes | GO:0002274 | myeloid leukocyte activation |
| GO Biological Processes | GO:0018958 | phenol-containing compound metabolic process |
| GO Biological Processes | GO:0010821 | regulation of mitochondrion organization |
| GO Biological Processes | GO:0032869 | cellular response to insulin stimulus |
| GO Biological Processes | GO:0106056 | regulation of calcineurin-mediated signaling |
| GO Biological Processes | GO:0007566 | embryo implantation |
| GO Biological Processes | GO:0042772 | DNA damage response, signal transduction resulting in transcription |
| GO Biological Processes | GO:0072331 | signal transduction by p53 class mediator |
| GO Biological Processes | GO:0050829 | defense response to Gram-negative bacterium |
| GO Biological Processes | GO:0034763 | negative regulation of transmembrane transport |
| GO Biological Processes | GO:0098869 | cellular oxidant detoxification |
| GO Biological Processes | GO:0050999 | regulation of nitric-oxide synthase activity |
| GO Biological Processes | GO:0014065 | phosphatidylinositol 3-kinase signaling |
| GO Biological Processes | GO:0014912 | negative regulation of smooth muscle cell migration |
| GO Biological Processes | GO:0045981 | positive regulation of nucleotide metabolic process |
| GO Biological Processes | GO:1900544 | positive regulation of purine nucleotide metabolic process |
| GO Biological Processes | GO:0045861 | negative regulation of proteolysis |
| GO Biological Processes | GO:0050851 | antigen receptor-mediated signaling pathway |
| GO Biological Processes | GO:0001776 | leukocyte homeostasis |
| GO Biological Processes | GO:2000106 | regulation of leukocyte apoptotic process |
| GO Biological Processes | GO:0000075 | cell cycle checkpoint signaling |
| GO Biological Processes | GO:0050805 | negative regulation of synaptic transmission |
| GO Biological Processes | GO:0008625 | extrinsic apoptotic signaling pathway via death domain receptors |
| GO Biological Processes | GO:0150076 | neuroinflammatory response |
| GO Biological Processes | GO:0031294 | lymphocyte costimulation |
| GO Biological Processes | GO:0010818 | T cell chemotaxis |
| GO Biological Processes | GO:0045348 | positive regulation of MHC class II biosynthetic process |
| GO Biological Processes | GO:1903798 | regulation of miRNA maturation |
| GO Biological Processes | GO:0070141 | response to UV-A |
| GO Biological Processes | GO:0009791 | post-embryonic development |
| GO Biological Processes | GO:1904646 | cellular response to amyloid-beta |
| GO Biological Processes | GO:0060443 | mammary gland morphogenesis |
| GO Biological Processes | GO:0010828 | positive regulation of glucose transmembrane transport |
| GO Biological Processes | GO:0002292 | T cell differentiation involved in immune response |
| GO Biological Processes | GO:0042303 | molting cycle |
| GO Biological Processes | GO:0051781 | positive regulation of cell division |
| GO Biological Processes | GO:0042633 | hair cycle |
| GO Biological Processes | GO:1900407 | regulation of cellular response to oxidative stress |
| GO Biological Processes | GO:0034248 | regulation of amide metabolic process |
| GO Biological Processes | GO:0030850 | prostate gland development |
| GO Biological Processes | GO:0030890 | positive regulation of B cell proliferation |
| GO Biological Processes | GO:1904706 | negative regulation of vascular associated smooth muscle cell proliferation |
| GO Biological Processes | GO:0046006 | regulation of activated T cell proliferation |
| GO Biological Processes | GO:0014014 | negative regulation of gliogenesis |
| GO Biological Processes | GO:0030307 | positive regulation of cell growth |
| GO Biological Processes | GO:2000671 | regulation of motor neuron apoptotic process |
| GO Biological Processes | GO:0034116 | positive regulation of heterotypic cell-cell adhesion |
| GO Biological Processes | GO:0043373 | CD4-positive, alpha-beta T cell lineage commitment |
| GO Biological Processes | GO:0097049 | motor neuron apoptotic process |
| GO Biological Processes | GO:0070920 | regulation of production of small RNA involved in gene silencing by RNA |
| GO Biological Processes | GO:1903409 | reactive oxygen species biosynthetic process |
| GO Biological Processes | GO:2000273 | positive regulation of signaling receptor activity |
| GO Biological Processes | GO:0022617 | extracellular matrix disassembly |
| GO Biological Processes | GO:0099536 | synaptic signaling |
| GO Biological Processes | GO:0048598 | embryonic morphogenesis |
| GO Biological Processes | GO:0048469 | cell maturation |
| GO Biological Processes | GO:0051972 | regulation of telomerase activity |
| GO Biological Processes | GO:0002861 | regulation of inflammatory response to antigenic stimulus |
| GO Biological Processes | GO:0048806 | genitalia development |
| GO Biological Processes | GO:0090199 | regulation of release of cytochrome c from mitochondria |
| GO Biological Processes | GO:0120162 | positive regulation of cold-induced thermogenesis |
| GO Biological Processes | GO:0002831 | regulation of response to biotic stimulus |
| GO Biological Processes | GO:2001243 | negative regulation of intrinsic apoptotic signaling pathway |
| GO Biological Processes | GO:0019218 | regulation of steroid metabolic process |
| GO Biological Processes | GO:1902003 | regulation of amyloid-beta formation |
| GO Biological Processes | GO:0031641 | regulation of myelination |
| GO Biological Processes | GO:0008610 | lipid biosynthetic process |
| GO Biological Processes | GO:0070230 | positive regulation of lymphocyte apoptotic process |
| GO Biological Processes | GO:0051044 | positive regulation of membrane protein ectodomain proteolysis |
| GO Biological Processes | GO:0050764 | regulation of phagocytosis |
| GO Biological Processes | GO:0002768 | immune response-regulating cell surface receptor signaling pathway |
| GO Biological Processes | GO:0034198 | cellular response to amino acid starvation |
| GO Biological Processes | GO:0033628 | regulation of cell adhesion mediated by integrin |
| GO Biological Processes | GO:0007596 | blood coagulation |
| GO Biological Processes | GO:0002253 | activation of immune response |
| GO Biological Processes | GO:0006937 | regulation of muscle contraction |
| GO Biological Processes | GO:0044282 | small molecule catabolic process |
| GO Biological Processes | GO:0050817 | coagulation |
| GO Biological Processes | GO:0010906 | regulation of glucose metabolic process |
| GO Biological Processes | GO:1902882 | regulation of response to oxidative stress |
| GO Biological Processes | GO:0048247 | lymphocyte chemotaxis |
| GO Biological Processes | GO:0045776 | negative regulation of blood pressure |
| GO Biological Processes | GO:0043457 | regulation of cellular respiration |
| GO Biological Processes | GO:0007204 | positive regulation of cytosolic calcium ion concentration |
| GO Biological Processes | GO:0048143 | astrocyte activation |
| GO Biological Processes | GO:1901550 | regulation of endothelial cell development |
| GO Biological Processes | GO:0009404 | toxin metabolic process |
| GO Biological Processes | GO:1903140 | regulation of establishment of endothelial barrier |
| GO Biological Processes | GO:0009299 | mRNA transcription |
| GO Biological Processes | GO:0007599 | hemostasis |
| GO Biological Processes | GO:0032760 | positive regulation of tumor necrosis factor production |
| GO Biological Processes | GO:0090596 | sensory organ morphogenesis |
| GO Biological Processes | GO:0090277 | positive regulation of peptide hormone secretion |
| GO Biological Processes | GO:0021700 | developmental maturation |
| GO Biological Processes | GO:0060324 | face development |
| GO Biological Processes | GO:1990928 | response to amino acid starvation |
| GO Biological Processes | GO:1905562 | regulation of vascular endothelial cell proliferation |
| GO Biological Processes | GO:0010543 | regulation of platelet activation |
| GO Biological Processes | GO:0007259 | receptor signaling pathway via JAK-STAT |
| GO Biological Processes | GO:0000904 | cell morphogenesis involved in differentiation |
| GO Biological Processes | GO:0043588 | skin development |
| GO Biological Processes | GO:0002793 | positive regulation of peptide secretion |
| GO Biological Processes | GO:0010883 | regulation of lipid storage |
| GO Biological Processes | GO:0071622 | regulation of granulocyte chemotaxis |
| GO Biological Processes | GO:0002639 | positive regulation of immunoglobulin production |
| GO Biological Processes | GO:0042100 | B cell proliferation |
| GO Biological Processes | GO:0097696 | receptor signaling pathway via STAT |
| GO Biological Processes | GO:0150078 | positive regulation of neuroinflammatory response |
| GO Biological Processes | GO:0036003 | positive regulation of transcription from RNA polymerase II promoter in response to stress |
| GO Biological Processes | GO:0035234 | ectopic germ cell programmed cell death |
| GO Biological Processes | GO:0090066 | regulation of anatomical structure size |
| GO Biological Processes | GO:1903557 | positive regulation of tumor necrosis factor superfamily cytokine production |
| GO Biological Processes | GO:0034605 | cellular response to heat |
| GO Biological Processes | GO:0072332 | intrinsic apoptotic signaling pathway by p53 class mediator |
| GO Biological Processes | GO:0071674 | mononuclear cell migration |
| GO Biological Processes | GO:1902991 | regulation of amyloid precursor protein catabolic process |
| GO Biological Processes | GO:0002686 | negative regulation of leukocyte migration |
| GO Biological Processes | GO:1903202 | negative regulation of oxidative stress-induced cell death |
| GO Biological Processes | GO:0051302 | regulation of cell division |
| GO Biological Processes | GO:0014066 | regulation of phosphatidylinositol 3-kinase signaling |
| GO Biological Processes | GO:0032651 | regulation of interleukin-1 beta production |
| GO Biological Processes | GO:1905954 | positive regulation of lipid localization |
| GO Biological Processes | GO:0045346 | regulation of MHC class II biosynthetic process |
| GO Biological Processes | GO:0002523 | leukocyte migration involved in inflammatory response |
| GO Biological Processes | GO:0002281 | macrophage activation involved in immune response |
| GO Biological Processes | GO:0007254 | JNK cascade |
| GO Biological Processes | GO:0006749 | glutathione metabolic process |
| GO Biological Processes | GO:0007420 | brain development |
| GO Biological Processes | GO:0043470 | regulation of carbohydrate catabolic process |
| GO Biological Processes | GO:0043388 | positive regulation of DNA binding |
| GO Biological Processes | GO:0019730 | antimicrobial humoral response |
| GO Biological Processes | GO:0048259 | regulation of receptor-mediated endocytosis |
| GO Biological Processes | GO:0043123 | positive regulation of I-kappaB kinase/NF-kappaB signaling |
| GO Biological Processes | GO:0050901 | leukocyte tethering or rolling |
| GO Biological Processes | GO:1900221 | regulation of amyloid-beta clearance |
| GO Biological Processes | GO:0061377 | mammary gland lobule development |
| GO Biological Processes | GO:0060749 | mammary gland alveolus development |
| GO Biological Processes | GO:0000902 | cell morphogenesis |
| GO Biological Processes | GO:0010718 | positive regulation of epithelial to mesenchymal transition |
| GO Biological Processes | GO:0061180 | mammary gland epithelium development |
| GO Biological Processes | GO:0007088 | regulation of mitotic nuclear division |
| GO Biological Processes | GO:0007006 | mitochondrial membrane organization |
| GO Biological Processes | GO:0031175 | neuron projection development |
| GO Biological Processes | GO:0016485 | protein processing |
| GO Biological Processes | GO:0010574 | regulation of vascular endothelial growth factor production |
| GO Biological Processes | GO:0002763 | positive regulation of myeloid leukocyte differentiation |
| GO Biological Processes | GO:0070886 | positive regulation of calcineurin-NFAT signaling cascade |
| GO Biological Processes | GO:0060252 | positive regulation of glial cell proliferation |
| GO Biological Processes | GO:0106058 | positive regulation of calcineurin-mediated signaling |
| GO Biological Processes | GO:0010888 | negative regulation of lipid storage |
| GO Biological Processes | GO:0071637 | regulation of monocyte chemotactic protein-1 production |
| GO Biological Processes | GO:1901739 | regulation of myoblast fusion |
| GO Biological Processes | GO:0071498 | cellular response to fluid shear stress |
| GO Biological Processes | GO:0046824 | positive regulation of nucleocytoplasmic transport |
| GO Biological Processes | GO:0042116 | macrophage activation |
| GO Biological Processes | GO:0035051 | cardiocyte differentiation |
| GO Biological Processes | GO:0050994 | regulation of lipid catabolic process |
| GO Biological Processes | GO:0043393 | regulation of protein binding |
| GO Biological Processes | GO:0007093 | mitotic cell cycle checkpoint signaling |
| GO Biological Processes | GO:0002824 | positive regulation of adaptive immune response based on somatic recombination of immune receptors built from immunoglobulin superfamily domains |
| GO Biological Processes | GO:0090303 | positive regulation of wound healing |
| GO Biological Processes | GO:0061756 | leukocyte adhesion to vascular endothelial cell |
| GO Biological Processes | GO:2000310 | regulation of NMDA receptor activity |
| GO Biological Processes | GO:0032891 | negative regulation of organic acid transport |
| GO Biological Processes | GO:0032303 | regulation of icosanoid secretion |
| GO Biological Processes | GO:0051769 | regulation of nitric-oxide synthase biosynthetic process |
| GO Biological Processes | GO:0055065 | metal ion homeostasis |
| GO Biological Processes | GO:0098916 | anterograde trans-synaptic signaling |
| GO Biological Processes | GO:0007268 | chemical synaptic transmission |
| GO Biological Processes | GO:0032731 | positive regulation of interleukin-1 beta production |
| GO Biological Processes | GO:0002712 | regulation of B cell mediated immunity |
| GO Biological Processes | GO:0099601 | regulation of neurotransmitter receptor activity |
| GO Biological Processes | GO:0051353 | positive regulation of oxidoreductase activity |
| GO Biological Processes | GO:0033077 | T cell differentiation in thymus |
| GO Biological Processes | GO:2001244 | positive regulation of intrinsic apoptotic signaling pathway |
| GO Biological Processes | GO:0001655 | urogenital system development |
| GO Biological Processes | GO:0070542 | response to fatty acid |
| GO Biological Processes | GO:0002889 | regulation of immunoglobulin mediated immune response |
| GO Biological Processes | GO:0002708 | positive regulation of lymphocyte mediated immunity |
| GO Biological Processes | GO:0038127 | ERBB signaling pathway |
| GO Biological Processes | GO:0040014 | regulation of multicellular organism growth |
| GO Biological Processes | GO:0042440 | pigment metabolic process |
| GO Biological Processes | GO:0002275 | myeloid cell activation involved in immune response |
| GO Biological Processes | GO:0006940 | regulation of smooth muscle contraction |
| GO Biological Processes | GO:0042593 | glucose homeostasis |
| GO Biological Processes | GO:0002446 | neutrophil mediated immunity |
| GO Biological Processes | GO:0060740 | prostate gland epithelium morphogenesis |
| GO Biological Processes | GO:0009651 | response to salt stress |
| GO Biological Processes | GO:0010893 | positive regulation of steroid biosynthetic process |
| GO Biological Processes | GO:0008016 | regulation of heart contraction |
| GO Biological Processes | GO:0045732 | positive regulation of protein catabolic process |
| GO Biological Processes | GO:0033500 | carbohydrate homeostasis |
| GO Biological Processes | GO:0002260 | lymphocyte homeostasis |
| GO Biological Processes | GO:0002821 | positive regulation of adaptive immune response |
| GO Biological Processes | GO:0032874 | positive regulation of stress-activated MAPK cascade |
| GO Biological Processes | GO:0035270 | endocrine system development |
| GO Biological Processes | GO:0046683 | response to organophosphorus |
| GO Biological Processes | GO:0007281 | germ cell development |
| GO Biological Processes | GO:0032094 | response to food |
| GO Biological Processes | GO:0045932 | negative regulation of muscle contraction |
| GO Biological Processes | GO:0030100 | regulation of endocytosis |
| GO Biological Processes | GO:0070304 | positive regulation of stress-activated protein kinase signaling cascade |
| GO Biological Processes | GO:0099537 | trans-synaptic signaling |
| GO Biological Processes | GO:0051282 | regulation of sequestering of calcium ion |
| GO Biological Processes | GO:0050890 | cognition |
| GO Biological Processes | GO:0007188 | adenylate cyclase-modulating G protein-coupled receptor signaling pathway |
| GO Biological Processes | GO:0098771 | inorganic ion homeostasis |
| GO Biological Processes | GO:1900180 | regulation of protein localization to nucleus |
| GO Biological Processes | GO:0072073 | kidney epithelium development |
| GO Biological Processes | GO:0060512 | prostate gland morphogenesis |
| GO Biological Processes | GO:0045662 | negative regulation of myoblast differentiation |
| GO Biological Processes | GO:2000679 | positive regulation of transcription regulatory region DNA binding |
| GO Biological Processes | GO:0034114 | regulation of heterotypic cell-cell adhesion |
| GO Biological Processes | GO:0097300 | programmed necrotic cell death |
| GO Biological Processes | GO:0006066 | alcohol metabolic process |
| GO Biological Processes | GO:0042098 | T cell proliferation |
| GO Biological Processes | GO:0014015 | positive regulation of gliogenesis |
| GO Biological Processes | GO:0008544 | epidermis development |
| GO Biological Processes | GO:0051129 | negative regulation of cellular component organization |
| GO Biological Processes | GO:0030193 | regulation of blood coagulation |
| GO Biological Processes | GO:0051881 | regulation of mitochondrial membrane potential |
| GO Biological Processes | GO:0043627 | response to estrogen |
| GO Biological Processes | GO:0070374 | positive regulation of ERK1 and ERK2 cascade |
| GO Biological Processes | GO:0045822 | negative regulation of heart contraction |
| GO Biological Processes | GO:0045672 | positive regulation of osteoclast differentiation |
| GO Biological Processes | GO:0002726 | positive regulation of T cell cytokine production |
| GO Biological Processes | GO:0002443 | leukocyte mediated immunity |
| GO Biological Processes | GO:0007005 | mitochondrion organization |
| GO Biological Processes | GO:1900046 | regulation of hemostasis |
| GO Biological Processes | GO:0071677 | positive regulation of mononuclear cell migration |
| GO Biological Processes | GO:0051926 | negative regulation of calcium ion transport |
| GO Biological Processes | GO:2001235 | positive regulation of apoptotic signaling pathway |
| GO Biological Processes | GO:0043271 | negative regulation of monoatomic ion transport |
| GO Biological Processes | GO:0045940 | positive regulation of steroid metabolic process |
| GO Biological Processes | GO:0002092 | positive regulation of receptor internalization |
| GO Biological Processes | GO:0042537 | benzene-containing compound metabolic process |
| GO Biological Processes | GO:1903523 | negative regulation of blood circulation |
| GO Biological Processes | GO:0090200 | positive regulation of release of cytochrome c from mitochondria |
| GO Biological Processes | GO:2000108 | positive regulation of leukocyte apoptotic process |
| GO Biological Processes | GO:0006936 | muscle contraction |
| GO Biological Processes | GO:0046637 | regulation of alpha-beta T cell differentiation |
| GO Biological Processes | GO:0050766 | positive regulation of phagocytosis |
| GO Biological Processes | GO:0060537 | muscle tissue development |
| GO Biological Processes | GO:0002429 | immune response-activating cell surface receptor signaling pathway |
| GO Biological Processes | GO:0061045 | negative regulation of wound healing |
| GO Biological Processes | GO:0032732 | positive regulation of interleukin-1 production |
| GO Biological Processes | GO:1903578 | regulation of ATP metabolic process |
| GO Biological Processes | GO:0031343 | positive regulation of cell killing |
| GO Biological Processes | GO:0033365 | protein localization to organelle |
| GO Biological Processes | GO:0010822 | positive regulation of mitochondrion organization |
| GO Biological Processes | GO:0050818 | regulation of coagulation |
| GO Biological Processes | GO:0030194 | positive regulation of blood coagulation |
| GO Biological Processes | GO:1900048 | positive regulation of hemostasis |
| GO Biological Processes | GO:0030878 | thyroid gland development |
| GO Biological Processes | GO:0001816 | cytokine production |
| GO Biological Processes | GO:0070265 | necrotic cell death |
| GO Biological Processes | GO:0042104 | positive regulation of activated T cell proliferation |
| GO Biological Processes | GO:0051783 | regulation of nuclear division |
| GO Biological Processes | GO:0120254 | olefinic compound metabolic process |
| GO Biological Processes | GO:0019233 | sensory perception of pain |
| GO Biological Processes | GO:0050810 | regulation of steroid biosynthetic process |
| GO Biological Processes | GO:0010508 | positive regulation of autophagy |
| GO Biological Processes | GO:0006869 | lipid transport |
| GO Biological Processes | GO:0007292 | female gamete generation |
| GO Biological Processes | GO:0045685 | regulation of glial cell differentiation |
| GO Biological Processes | GO:0001937 | negative regulation of endothelial cell proliferation |
| GO Biological Processes | GO:0001942 | hair follicle development |
| GO Biological Processes | GO:2000112 | regulation of cellular macromolecule biosynthetic process |
| GO Biological Processes | GO:0008209 | androgen metabolic process |
| GO Biological Processes | GO:0033280 | response to vitamin D |
| GO Biological Processes | GO:1900017 | positive regulation of cytokine production involved in inflammatory response |
| GO Biological Processes | GO:0090287 | regulation of cellular response to growth factor stimulus |
| GO Biological Processes | GO:0043154 | negative regulation of cysteine-type endopeptidase activity involved in apoptotic process |
| GO Biological Processes | GO:0072676 | lymphocyte migration |
| GO Biological Processes | GO:0009749 | response to glucose |
| GO Biological Processes | GO:0072594 | establishment of protein localization to organelle |
| GO Biological Processes | GO:0005975 | carbohydrate metabolic process |
| GO Biological Processes | GO:0006839 | mitochondrial transport |
| GO Biological Processes | GO:1903038 | negative regulation of leukocyte cell-cell adhesion |
| GO Biological Processes | GO:0060485 | mesenchyme development |
| GO Biological Processes | GO:0050820 | positive regulation of coagulation |
| GO Biological Processes | GO:0060142 | regulation of syncytium formation by plasma membrane fusion |
| GO Biological Processes | GO:0001662 | behavioral fear response |
| GO Biological Processes | GO:0001774 | microglial cell activation |
| GO Biological Processes | GO:0090330 | regulation of platelet aggregation |
| GO Biological Processes | GO:0042771 | intrinsic apoptotic signaling pathway in response to DNA damage by p53 class mediator |
| GO Biological Processes | GO:0090312 | positive regulation of protein deacetylation |
| GO Biological Processes | GO:0016525 | negative regulation of angiogenesis |
| GO Biological Processes | GO:0002720 | positive regulation of cytokine production involved in immune response |
| GO Biological Processes | GO:2000181 | negative regulation of blood vessel morphogenesis |
| GO Biological Processes | GO:0030163 | protein catabolic process |
| GO Biological Processes | GO:0022405 | hair cycle process |
| GO Biological Processes | GO:0022404 | molting cycle process |
| GO Biological Processes | GO:1901343 | negative regulation of vasculature development |
| GO Biological Processes | GO:0006913 | nucleocytoplasmic transport |
| GO Biological Processes | GO:0051169 | nuclear transport |
| GO Biological Processes | GO:0002209 | behavioral defense response |
| GO Biological Processes | GO:0045987 | positive regulation of smooth muscle contraction |
| GO Biological Processes | GO:0001782 | B cell homeostasis |
| GO Biological Processes | GO:0043304 | regulation of mast cell degranulation |
| GO Biological Processes | GO:1902042 | negative regulation of extrinsic apoptotic signaling pathway via death domain receptors |
| GO Biological Processes | GO:0045948 | positive regulation of translational initiation |
| GO Biological Processes | GO:0010039 | response to iron ion |
| GO Biological Processes | GO:1900745 | positive regulation of p38MAPK cascade |
| GO Biological Processes | GO:0095500 | acetylcholine receptor signaling pathway |
| GO Biological Processes | GO:1903203 | regulation of oxidative stress-induced neuron death |
| GO Biological Processes | GO:0030902 | hindbrain development |
| GO Biological Processes | GO:0048592 | eye morphogenesis |
| GO Biological Processes | GO:0090316 | positive regulation of intracellular protein transport |
| GO Biological Processes | GO:0051149 | positive regulation of muscle cell differentiation |
| GO Biological Processes | GO:0042475 | odontogenesis of dentin-containing tooth |
| GO Biological Processes | GO:0001569 | branching involved in blood vessel morphogenesis |
| GO Biological Processes | GO:0031069 | hair follicle morphogenesis |
| GO Biological Processes | GO:0002862 | negative regulation of inflammatory response to antigenic stimulus |
| GO Biological Processes | GO:0001523 | retinoid metabolic process |
| GO Biological Processes | GO:0070098 | chemokine-mediated signaling pathway |
| GO Biological Processes | GO:0045926 | negative regulation of growth |
| GO Biological Processes | GO:0001657 | ureteric bud development |
| GO Biological Processes | GO:0010951 | negative regulation of endopeptidase activity |
| GO Biological Processes | GO:0055094 | response to lipoprotein particle |
| GO Biological Processes | GO:0033006 | regulation of mast cell activation involved in immune response |
| GO Biological Processes | GO:0048566 | embryonic digestive tract development |
| GO Biological Processes | GO:1905145 | cellular response to acetylcholine |
| GO Biological Processes | GO:0071404 | cellular response to low-density lipoprotein particle stimulus |
| GO Biological Processes | GO:0090183 | regulation of kidney development |
| GO Biological Processes | GO:0002269 | leukocyte activation involved in inflammatory response |
| GO Biological Processes | GO:0051147 | regulation of muscle cell differentiation |
| GO Biological Processes | GO:0006898 | receptor-mediated endocytosis |
| GO Biological Processes | GO:0072164 | mesonephric tubule development |
| GO Biological Processes | GO:0072163 | mesonephric epithelium development |
| GO Biological Processes | GO:0016101 | diterpenoid metabolic process |
| GO Biological Processes | GO:2000243 | positive regulation of reproductive process |
| GO Biological Processes | GO:0032370 | positive regulation of lipid transport |
| GO Biological Processes | GO:1901361 | organic cyclic compound catabolic process |
| GO Biological Processes | GO:0035794 | positive regulation of mitochondrial membrane permeability |
| GO Biological Processes | GO:0009112 | nucleobase metabolic process |
| GO Biological Processes | GO:0051973 | positive regulation of telomerase activity |
| GO Biological Processes | GO:0010661 | positive regulation of muscle cell apoptotic process |
| GO Biological Processes | GO:0070059 | intrinsic apoptotic signaling pathway in response to endoplasmic reticulum stress |
| GO Biological Processes | GO:0090322 | regulation of superoxide metabolic process |
| GO Biological Processes | GO:0046661 | male sex differentiation |
| GO Biological Processes | GO:0060993 | kidney morphogenesis |
| GO Biological Processes | GO:0001892 | embryonic placenta development |
| GO Biological Processes | GO:0021675 | nerve development |
| GO Biological Processes | GO:0055001 | muscle cell development |
| GO Biological Processes | GO:1903035 | negative regulation of response to wounding |
| GO Biological Processes | GO:0071229 | cellular response to acid chemical |
| GO Biological Processes | GO:0090050 | positive regulation of cell migration involved in sprouting angiogenesis |
| GO Biological Processes | GO:0001893 | maternal placenta development |
| GO Biological Processes | GO:0002719 | negative regulation of cytokine production involved in immune response |
| GO Biological Processes | GO:0035633 | maintenance of blood-brain barrier |
| GO Biological Processes | GO:0042596 | fear response |
| GO Biological Processes | GO:0001823 | mesonephros development |
| GO Biological Processes | GO:0001501 | skeletal system development |
| GO Biological Processes | GO:2000117 | negative regulation of cysteine-type endopeptidase activity |
| GO Biological Processes | GO:0048730 | epidermis morphogenesis |
| GO Biological Processes | GO:0031929 | TOR signaling |
| GO Biological Processes | GO:1905332 | positive regulation of morphogenesis of an epithelium |
| GO Biological Processes | GO:0051930 | regulation of sensory perception of pain |
| GO Biological Processes | GO:0051931 | regulation of sensory perception |
| GO Biological Processes | GO:0071402 | cellular response to lipoprotein particle stimulus |
| GO Biological Processes | GO:1903580 | positive regulation of ATP metabolic process |
| GO Biological Processes | GO:0014002 | astrocyte development |
| GO Biological Processes | GO:1990869 | cellular response to chemokine |
| GO Biological Processes | GO:1990868 | response to chemokine |
| GO Biological Processes | GO:0051235 | maintenance of location |
| GO Biological Processes | GO:0009308 | amine metabolic process |
| GO Biological Processes | GO:0046326 | positive regulation of glucose import |
| GO Biological Processes | GO:0034110 | regulation of homotypic cell-cell adhesion |
| GO Biological Processes | GO:1905475 | regulation of protein localization to membrane |
| GO Biological Processes | GO:0051604 | protein maturation |
| GO Biological Processes | GO:0019217 | regulation of fatty acid metabolic process |
| GO Biological Processes | GO:0045191 | regulation of isotype switching |
| GO Biological Processes | GO:0002724 | regulation of T cell cytokine production |
| GO Biological Processes | GO:0045923 | positive regulation of fatty acid metabolic process |
| GO Biological Processes | GO:0001974 | blood vessel remodeling |
| GO Biological Processes | GO:0032885 | regulation of polysaccharide biosynthetic process |
| GO Biological Processes | GO:0051099 | positive regulation of binding |
| GO Biological Processes | GO:0007611 | learning or memory |
| GO Biological Processes | GO:0070664 | negative regulation of leukocyte proliferation |
| GO Biological Processes | GO:0002709 | regulation of T cell mediated immunity |
| GO Biological Processes | GO:0097306 | cellular response to alcohol |
| GO Biological Processes | GO:0045740 | positive regulation of DNA replication |
| GO Biological Processes | GO:0048645 | animal organ formation |
| GO Biological Processes | GO:0051145 | smooth muscle cell differentiation |
| GO Biological Processes | GO:1905710 | positive regulation of membrane permeability |
| GO Biological Processes | GO:0042307 | positive regulation of protein import into nucleus |
| GO Biological Processes | GO:0006721 | terpenoid metabolic process |
| GO Biological Processes | GO:0030324 | lung development |
| GO Biological Processes | GO:0008210 | estrogen metabolic process |
| GO Biological Processes | GO:0010863 | positive regulation of phospholipase C activity |
| GO Biological Processes | GO:0042551 | neuron maturation |
| GO Biological Processes | GO:0051349 | positive regulation of lyase activity |
| GO Biological Processes | GO:0032147 | activation of protein kinase activity |
| GO Biological Processes | GO:0010631 | epithelial cell migration |
| GO Biological Processes | GO:0023061 | signal release |
| GO Biological Processes | GO:0021543 | pallium development |
| GO Biological Processes | GO:0031589 | cell-substrate adhesion |
| GO Biological Processes | GO:0030323 | respiratory tube development |
| GO Biological Processes | GO:0071470 | cellular response to osmotic stress |
| GO Biological Processes | GO:0033574 | response to testosterone |
| GO Biological Processes | GO:0002251 | organ or tissue specific immune response |
| GO Biological Processes | GO:0031295 | T cell costimulation |
| GO Biological Processes | GO:0032965 | regulation of collagen biosynthetic process |
| GO Biological Processes | GO:0002691 | regulation of cellular extravasation |
| GO Biological Processes | GO:0051896 | regulation of protein kinase B signaling |
| GO Biological Processes | GO:1905477 | positive regulation of protein localization to membrane |
| GO Biological Processes | GO:2000008 | regulation of protein localization to cell surface |
| GO Biological Processes | GO:0002891 | positive regulation of immunoglobulin mediated immune response |
| GO Biological Processes | GO:0002714 | positive regulation of B cell mediated immunity |
| GO Biological Processes | GO:1900274 | regulation of phospholipase C activity |
| GO Biological Processes | GO:0019915 | lipid storage |
| GO Biological Processes | GO:0090132 | epithelium migration |
| GO Biological Processes | GO:1903076 | regulation of protein localization to plasma membrane |
| GO Biological Processes | GO:0072009 | nephron epithelium development |
| GO Biological Processes | GO:0050881 | musculoskeletal movement |
| GO Biological Processes | GO:0006584 | catecholamine metabolic process |
| GO Biological Processes | GO:0038061 | NIK/NF-kappaB signaling |
| GO Biological Processes | GO:0009712 | catechol-containing compound metabolic process |
| GO Biological Processes | GO:0034101 | erythrocyte homeostasis |
| GO Biological Processes | GO:1901655 | cellular response to ketone |
| GO Biological Processes | GO:0030308 | negative regulation of cell growth |
| GO Biological Processes | GO:0033003 | regulation of mast cell activation |
| GO Biological Processes | GO:0050879 | multicellular organismal movement |
| GO Biological Processes | GO:0032881 | regulation of polysaccharide metabolic process |
| GO Biological Processes | GO:0062014 | negative regulation of small molecule metabolic process |
| GO Biological Processes | GO:0046822 | regulation of nucleocytoplasmic transport |
| GO Biological Processes | GO:0098773 | skin epidermis development |
| GO Biological Processes | GO:0043433 | negative regulation of DNA-binding transcription factor activity |
| GO Biological Processes | GO:0017157 | regulation of exocytosis |
| GO Biological Processes | GO:0007423 | sensory organ development |
| GO Biological Processes | GO:0006897 | endocytosis |
| GO Biological Processes | GO:0048599 | oocyte development |
| GO Biological Processes | GO:1902622 | regulation of neutrophil migration |
| GO Biological Processes | GO:0048483 | autonomic nervous system development |
| GO Biological Processes | GO:0001658 | branching involved in ureteric bud morphogenesis |
| GO Biological Processes | GO:1900744 | regulation of p38MAPK cascade |
| GO Biological Processes | GO:1902692 | regulation of neuroblast proliferation |
| GO Biological Processes | GO:0022412 | cellular process involved in reproduction in multicellular organism |
| GO Biological Processes | GO:1902106 | negative regulation of leukocyte differentiation |
| GO Biological Processes | GO:0090130 | tissue migration |
| GO Biological Processes | GO:0001667 | ameboidal-type cell migration |
| GO Biological Processes | GO:0032388 | positive regulation of intracellular transport |
| GO Biological Processes | GO:0010717 | regulation of epithelial to mesenchymal transition |
| GO Biological Processes | GO:0030195 | negative regulation of blood coagulation |
| GO Biological Processes | GO:0010830 | regulation of myotube differentiation |
| GO Biological Processes | GO:0010712 | regulation of collagen metabolic process |
| GO Biological Processes | GO:0042789 | mRNA transcription by RNA polymerase II |
| GO Biological Processes | GO:0006874 | cellular calcium ion homeostasis |
| GO Biological Processes | GO:0007517 | muscle organ development |
| GO Biological Processes | GO:0021782 | glial cell development |
| GO Biological Processes | GO:0006778 | porphyrin-containing compound metabolic process |
| GO Biological Processes | GO:1900047 | negative regulation of hemostasis |
| GO Biological Processes | GO:0046677 | response to antibiotic |
| GO Biological Processes | GO:0060711 | labyrinthine layer development |
| GO Biological Processes | GO:0042220 | response to cocaine |
| GO Biological Processes | GO:0002701 | negative regulation of production of molecular mediator of immune response |
| GO Biological Processes | GO:0002695 | negative regulation of leukocyte activation |
| GO Biological Processes | GO:2001020 | regulation of response to DNA damage stimulus |
| GO Biological Processes | GO:0046634 | regulation of alpha-beta T cell activation |
| GO Biological Processes | GO:0045744 | negative regulation of G protein-coupled receptor signaling pathway |
| GO Biological Processes | GO:0043300 | regulation of leukocyte degranulation |
| GO Biological Processes | GO:0032660 | regulation of interleukin-17 production |
| GO Biological Processes | GO:0042311 | vasodilation |
| GO Biological Processes | GO:0006110 | regulation of glycolytic process |
| GO Biological Processes | GO:0032892 | positive regulation of organic acid transport |
| GO Biological Processes | GO:0060541 | respiratory system development |
| GO Biological Processes | GO:0022411 | cellular component disassembly |
| GO Biological Processes | GO:0022037 | metencephalon development |
| GO Biological Processes | GO:1903707 | negative regulation of hemopoiesis |
| GO Biological Processes | GO:0021761 | limbic system development |
| GO Biological Processes | GO:0002250 | adaptive immune response |
| GO Biological Processes | GO:0042752 | regulation of circadian rhythm |
| GO Biological Processes | GO:0042149 | cellular response to glucose starvation |
| GO Biological Processes | GO:0009994 | oocyte differentiation |
| GO Biological Processes | GO:0048546 | digestive tract morphogenesis |
| GO Biological Processes | GO:0045933 | positive regulation of muscle contraction |
| GO Biological Processes | GO:0022602 | ovulation cycle process |
| GO Biological Processes | GO:0003014 | renal system process |
| GO Biological Processes | GO:0006720 | isoprenoid metabolic process |
| GO Biological Processes | GO:0001505 | regulation of neurotransmitter levels |
| GO Biological Processes | GO:0050819 | negative regulation of coagulation |
| GO Biological Processes | GO:1902041 | regulation of extrinsic apoptotic signaling pathway via death domain receptors |
| GO Biological Processes | GO:0046456 | icosanoid biosynthetic process |
| GO Biological Processes | GO:0032649 | regulation of type II interferon production |
| GO Biological Processes | GO:0031647 | regulation of protein stability |
| GO Biological Processes | GO:1904707 | positive regulation of vascular associated smooth muscle cell proliferation |
| GO Biological Processes | GO:1901989 | positive regulation of cell cycle phase transition |
| GO Biological Processes | GO:0097194 | execution phase of apoptosis |
| GO Biological Processes | GO:0007157 | heterophilic cell-cell adhesion via plasma membrane cell adhesion molecules |
| GO Biological Processes | GO:2000677 | regulation of transcription regulatory region DNA binding |
| GO Biological Processes | GO:0002762 | negative regulation of myeloid leukocyte differentiation |
| GO Biological Processes | GO:0046622 | positive regulation of organ growth |
| GO Biological Processes | GO:0032386 | regulation of intracellular transport |
| GO Biological Processes | GO:0030316 | osteoclast differentiation |
| GO Biological Processes | GO:0010823 | negative regulation of mitochondrion organization |
| GO Biological Processes | GO:0048146 | positive regulation of fibroblast proliferation |
| GO Biological Processes | GO:0051155 | positive regulation of striated muscle cell differentiation |
| GO Biological Processes | GO:0140352 | export from cell |
| GO Biological Processes | GO:0042476 | odontogenesis |
| GO Biological Processes | GO:1903170 | negative regulation of calcium ion transmembrane transport |
| GO Biological Processes | GO:0034976 | response to endoplasmic reticulum stress |
| GO Biological Processes | GO:0061448 | connective tissue development |
| GO Biological Processes | GO:2000027 | regulation of animal organ morphogenesis |
| GO Biological Processes | GO:0048593 | camera-type eye morphogenesis |
| GO Biological Processes | GO:0010518 | positive regulation of phospholipase activity |
| GO Biological Processes | GO:0043370 | regulation of CD4-positive, alpha-beta T cell differentiation |
| GO Biological Processes | GO:0070371 | ERK1 and ERK2 cascade |
| GO Biological Processes | GO:0033013 | tetrapyrrole metabolic process |
| GO Biological Processes | GO:0060675 | ureteric bud morphogenesis |
| GO Biological Processes | GO:0033157 | regulation of intracellular protein transport |
| GO Biological Processes | GO:1904375 | regulation of protein localization to cell periphery |
| GO Biological Processes | GO:0055074 | calcium ion homeostasis |
| GO Biological Processes | GO:0007187 | G protein-coupled receptor signaling pathway, coupled to cyclic nucleotide second messenger |
| GO Biological Processes | GO:0010559 | regulation of glycoprotein biosynthetic process |
| GO Biological Processes | GO:0072171 | mesonephric tubule morphogenesis |
| GO Biological Processes | GO:0097006 | regulation of plasma lipoprotein particle levels |
| GO Biological Processes | GO:0002823 | negative regulation of adaptive immune response based on somatic recombination of immune receptors built from immunoglobulin superfamily domains |
| GO Biological Processes | GO:0045931 | positive regulation of mitotic cell cycle |
| GO Biological Processes | GO:1903305 | regulation of regulated secretory pathway |
| GO Biological Processes | GO:0050868 | negative regulation of T cell activation |
| GO Biological Processes | GO:0006790 | sulfur compound metabolic process |
| GO Biological Processes | GO:0042306 | regulation of protein import into nucleus |
| GO Biological Processes | GO:0090311 | regulation of protein deacetylation |
| GO Biological Processes | GO:0072006 | nephron development |
| GO Biological Processes | GO:0007519 | skeletal muscle tissue development |
| GO Biological Processes | GO:0032990 | cell part morphogenesis |
| GO Biological Processes | GO:1903053 | regulation of extracellular matrix organization |
| GO Biological Processes | GO:0033209 | tumor necrosis factor-mediated signaling pathway |
| GO Biological Processes | GO:0050807 | regulation of synapse organization |
| GO Biological Processes | GO:0010565 | regulation of cellular ketone metabolic process |
| GO Biological Processes | GO:0010466 | negative regulation of peptidase activity |
| GO Biological Processes | GO:0006275 | regulation of DNA replication |
| GO Biological Processes | GO:0008277 | regulation of G protein-coupled receptor signaling pathway |
| GO Biological Processes | GO:0002886 | regulation of myeloid leukocyte mediated immunity |
| GO Biological Processes | GO:1903078 | positive regulation of protein localization to plasma membrane |
| GO Biological Processes | GO:0003179 | heart valve morphogenesis |
| GO Biological Processes | GO:0006939 | smooth muscle contraction |
| GO Biological Processes | GO:0043407 | negative regulation of MAP kinase activity |
| GO Biological Processes | GO:0007623 | circadian rhythm |
| GO Biological Processes | GO:0019229 | regulation of vasoconstriction |
| GO Biological Processes | GO:0002820 | negative regulation of adaptive immune response |
| GO Biological Processes | GO:0048678 | response to axon injury |
| GO Biological Processes | GO:0050803 | regulation of synapse structure or activity |
| GO Biological Processes | GO:0046328 | regulation of JNK cascade |
| GO Biological Processes | GO:0060261 | positive regulation of transcription initiation by RNA polymerase II |
| GO Biological Processes | GO:0043030 | regulation of macrophage activation |
| GO Biological Processes | GO:0009620 | response to fungus |
| GO Biological Processes | GO:0043506 | regulation of JUN kinase activity |
| GO Biological Processes | GO:1903018 | regulation of glycoprotein metabolic process |
| GO Biological Processes | GO:0008584 | male gonad development |
| GO Biological Processes | GO:0045727 | positive regulation of translation |
| GO Biological Processes | GO:0046546 | development of primary male sexual characteristics |
| GO Biological Processes | GO:0032434 | regulation of proteasomal ubiquitin-dependent protein catabolic process |
| GO Biological Processes | GO:0031056 | regulation of histone modification |
| GO Biological Processes | GO:0002090 | regulation of receptor internalization |
| GO Biological Processes | GO:0050905 | neuromuscular process |
| GO Biological Processes | GO:0007264 | small GTPase mediated signal transduction |
| GO Biological Processes | GO:0051339 | regulation of lyase activity |
| GO Biological Processes | GO:0045088 | regulation of innate immune response |
| GO Biological Processes | GO:0001654 | eye development |
| GO Biological Processes | GO:0033044 | regulation of chromosome organization |
| GO Biological Processes | GO:0072078 | nephron tubule morphogenesis |
| GO Biological Processes | GO:2001259 | positive regulation of cation channel activity |
| GO Biological Processes | GO:0006112 | energy reserve metabolic process |
| GO Biological Processes | GO:0010517 | regulation of phospholipase activity |
| GO Biological Processes | GO:0060538 | skeletal muscle organ development |
| GO Biological Processes | GO:0150063 | visual system development |
| GO Biological Processes | GO:0051607 | defense response to virus |
| GO Biological Processes | GO:0140546 | defense response to symbiont |
| GO Biological Processes | GO:0060193 | positive regulation of lipase activity |
| GO Biological Processes | GO:1901224 | positive regulation of NIK/NF-kappaB signaling |
| GO Biological Processes | GO:0008286 | insulin receptor signaling pathway |
| GO Biological Processes | GO:2000144 | positive regulation of DNA-templated transcription initiation |
| GO Biological Processes | GO:0072088 | nephron epithelium morphogenesis |
| GO Biological Processes | GO:2000401 | regulation of lymphocyte migration |
| GO Biological Processes | GO:1904377 | positive regulation of protein localization to cell periphery |
| GO Biological Processes | GO:0048880 | sensory system development |
| GO Biological Processes | GO:0003007 | heart morphogenesis |
| GO Biological Processes | GO:0002711 | positive regulation of T cell mediated immunity |
| GO Biological Processes | GO:0030858 | positive regulation of epithelial cell differentiation |
| GO Biological Processes | GO:0006816 | calcium ion transport |
| GO Biological Processes | GO:0061564 | axon development |
| GO Biological Processes | GO:0061333 | renal tubule morphogenesis |
| GO Biological Processes | GO:0043542 | endothelial cell migration |
| GO Biological Processes | GO:0050922 | negative regulation of chemotaxis |
| GO Biological Processes | GO:0072028 | nephron morphogenesis |
| GO Biological Processes | GO:0006518 | peptide metabolic process |
| GO Biological Processes | GO:0031623 | receptor internalization |
| GO Biological Processes | GO:0060260 | regulation of transcription initiation by RNA polymerase II |
| GO Biological Processes | GO:0007589 | body fluid secretion |
| GO Biological Processes | GO:0046626 | regulation of insulin receptor signaling pathway |
| GO Biological Processes | GO:0030198 | extracellular matrix organization |
| GO Biological Processes | GO:0030900 | forebrain development |
| GO Biological Processes | GO:0043062 | extracellular structure organization |
| GO Biological Processes | GO:0003170 | heart valve development |
| GO Biological Processes | GO:0045229 | external encapsulating structure organization |
| GO Biological Processes | GO:0021537 | telencephalon development |
| GO Biological Processes | GO:0031016 | pancreas development |
| GO Biological Processes | GO:0015908 | fatty acid transport |
| GO Biological Processes | GO:0032940 | secretion by cell |
| GO Biological Processes | GO:0045669 | positive regulation of osteoblast differentiation |
| GO Biological Processes | GO:0016239 | positive regulation of macroautophagy |
| GO Biological Processes | GO:0007193 | adenylate cyclase-inhibiting G protein-coupled receptor signaling pathway |
| GO Biological Processes | GO:0001959 | regulation of cytokine-mediated signaling pathway |
| GO Biological Processes | GO:0021536 | diencephalon development |
| GO Biological Processes | GO:0032729 | positive regulation of type II interferon production |
| GO Biological Processes | GO:2000514 | regulation of CD4-positive, alpha-beta T cell activation |
| GO Biological Processes | GO:0051216 | cartilage development |
| GO Biological Processes | GO:0050709 | negative regulation of protein secretion |
| GO Biological Processes | GO:0032720 | negative regulation of tumor necrosis factor production |
| GO Biological Processes | GO:0006575 | cellular modified amino acid metabolic process |
| GO Biological Processes | GO:0051250 | negative regulation of lymphocyte activation |
| GO Biological Processes | GO:0032371 | regulation of sterol transport |
| GO Biological Processes | GO:0032374 | regulation of cholesterol transport |
| GO Biological Processes | GO:0048667 | cell morphogenesis involved in neuron differentiation |
| GO Biological Processes | GO:0022900 | electron transport chain |
| GO Biological Processes | GO:1903556 | negative regulation of tumor necrosis factor superfamily cytokine production |
| GO Biological Processes | GO:0071230 | cellular response to amino acid stimulus |
| GO Biological Processes | GO:0002062 | chondrocyte differentiation |
| GO Biological Processes | GO:2000142 | regulation of DNA-templated transcription initiation |
| GO Biological Processes | GO:0045921 | positive regulation of exocytosis |
| GO Biological Processes | GO:0090049 | regulation of cell migration involved in sprouting angiogenesis |
| GO Biological Processes | GO:0051966 | regulation of synaptic transmission, glutamatergic |
| GO Biological Processes | GO:0048762 | mesenchymal cell differentiation |
| GO Biological Processes | GO:0072080 | nephron tubule development |
| GO Biological Processes | GO:2000058 | regulation of ubiquitin-dependent protein catabolic process |
| GO Biological Processes | GO:2001021 | negative regulation of response to DNA damage stimulus |
| GO Biological Processes | GO:0010921 | regulation of phosphatase activity |
| GO Biological Processes | GO:0006446 | regulation of translational initiation |
| GO Biological Processes | GO:0060759 | regulation of response to cytokine stimulus |
| GO Biological Processes | GO:0002460 | adaptive immune response based on somatic recombination of immune receptors built from immunoglobulin superfamily domains |
| GO Biological Processes | GO:0006576 | biogenic amine metabolic process |
| GO Biological Processes | GO:0008643 | carbohydrate transport |
| GO Biological Processes | GO:0035264 | multicellular organism growth |
| GO Biological Processes | GO:0044344 | cellular response to fibroblast growth factor stimulus |
| GO Biological Processes | GO:0007265 | Ras protein signal transduction |
| GO Biological Processes | GO:0045069 | regulation of viral genome replication |
| GO Biological Processes | GO:0061326 | renal tubule development |
| GO Biological Processes | GO:0048864 | stem cell development |
| GO Biological Processes | GO:0032989 | cellular component morphogenesis |
| GO Biological Processes | GO:0001960 | negative regulation of cytokine-mediated signaling pathway |
| GO Biological Processes | GO:0043409 | negative regulation of MAPK cascade |
| GO Biological Processes | GO:0031058 | positive regulation of histone modification |
| GO Biological Processes | GO:0050672 | negative regulation of lymphocyte proliferation |
| GO Biological Processes | GO:0060079 | excitatory postsynaptic potential |
| GO Biological Processes | GO:0002064 | epithelial cell development |
| GO Biological Processes | GO:0048477 | oogenesis |
| GO Biological Processes | GO:0051591 | response to cAMP |
| GO Biological Processes | GO:2000177 | regulation of neural precursor cell proliferation |
| GO Biological Processes | GO:0032945 | negative regulation of mononuclear cell proliferation |
| GO Biological Processes | GO:0060191 | regulation of lipase activity |
| GO Biological Processes | GO:0046777 | protein autophosphorylation |
| GO Biological Processes | GO:0071774 | response to fibroblast growth factor |
| GO Biological Processes | GO:0060761 | negative regulation of response to cytokine stimulus |
| GO Biological Processes | GO:0048812 | neuron projection morphogenesis |
| GO Biological Processes | GO:0030641 | regulation of cellular pH |
| GO Biological Processes | GO:0032436 | positive regulation of proteasomal ubiquitin-dependent protein catabolic process |
| GO Biological Processes | GO:0003073 | regulation of systemic arterial blood pressure |
| GO Biological Processes | GO:0099565 | chemical synaptic transmission, postsynaptic |
| GO Biological Processes | GO:0061136 | regulation of proteasomal protein catabolic process |
| GO Biological Processes | GO:0045333 | cellular respiration |
| GO Biological Processes | GO:0120039 | plasma membrane bounded cell projection morphogenesis |
| GO Biological Processes | GO:0003158 | endothelium development |
| GO Biological Processes | GO:0046330 | positive regulation of JNK cascade |
| GO Biological Processes | GO:0045638 | negative regulation of myeloid cell differentiation |
| GO Biological Processes | GO:0043502 | regulation of muscle adaptation |
| GO Biological Processes | GO:0030218 | erythrocyte differentiation |
| GO Biological Processes | GO:0009913 | epidermal cell differentiation |
| GO Biological Processes | GO:0048858 | cell projection morphogenesis |
| GO Biological Processes | GO:1903321 | negative regulation of protein modification by small protein conjugation or removal |
| GO Biological Processes | GO:0051153 | regulation of striated muscle cell differentiation |
| GO Biological Processes | GO:0043010 | camera-type eye development |
| GO Biological Processes | GO:0002244 | hematopoietic progenitor cell differentiation |
| GO Biological Processes | GO:1901992 | positive regulation of mitotic cell cycle phase transition |
| GO Biological Processes | GO:0007178 | transmembrane receptor protein serine/threonine kinase signaling pathway |
| GO Biological Processes | GO:0043255 | regulation of carbohydrate biosynthetic process |
| GO Biological Processes | GO:0046620 | regulation of organ growth |
| GO Biological Processes | GO:1904063 | negative regulation of cation transmembrane transport |
| GO Biological Processes | GO:0006885 | regulation of pH |
| GO Biological Processes | GO:0032204 | regulation of telomere maintenance |
| GO Biological Processes | GO:0070588 | calcium ion transmembrane transport |
| GO Biological Processes | GO:0021549 | cerebellum development |
| GO Biological Processes | GO:0019439 | aromatic compound catabolic process |
| GO Biological Processes | GO:0015718 | monocarboxylic acid transport |
| GO Biological Processes | GO:0002027 | regulation of heart rate |
| GO Biological Processes | GO:0035265 | organ growth |
| GO Biological Processes | GO:0051346 | negative regulation of hydrolase activity |
| GO Biological Processes | GO:1901888 | regulation of cell junction assembly |
| GO Biological Processes | GO:0032526 | response to retinoic acid |
| GO Biological Processes | GO:0007200 | phospholipase C-activating G protein-coupled receptor signaling pathway |
| GO Biological Processes | GO:0034766 | negative regulation of monoatomic ion transmembrane transport |
| GO Biological Processes | GO:1901222 | regulation of NIK/NF-kappaB signaling |
| GO Biological Processes | GO:0006941 | striated muscle contraction |
| GO Biological Processes | GO:0060078 | regulation of postsynaptic membrane potential |
| GO Biological Processes | GO:2000060 | positive regulation of ubiquitin-dependent protein catabolic process |
| GO Biological Processes | GO:2001252 | positive regulation of chromosome organization |
| GO Biological Processes | GO:0010810 | regulation of cell-substrate adhesion |
| GO Biological Processes | GO:1902749 | regulation of cell cycle G2/M phase transition |
| GO Biological Processes | GO:0042177 | negative regulation of protein catabolic process |
| GO Biological Processes | GO:0000079 | regulation of cyclin-dependent protein serine/threonine kinase activity |
| GO Biological Processes | GO:0019827 | stem cell population maintenance |
| GO Biological Processes | GO:0046395 | carboxylic acid catabolic process |
| GO Biological Processes | GO:0016054 | organic acid catabolic process |
| GO Biological Processes | GO:1904029 | regulation of cyclin-dependent protein kinase activity |
| GO Biological Processes | GO:0010633 | negative regulation of epithelial cell migration |
| GO Biological Processes | GO:1903050 | regulation of proteolysis involved in protein catabolic process |
| GO Biological Processes | GO:1901800 | positive regulation of proteasomal protein catabolic process |
| GO Biological Processes | GO:0098727 | maintenance of cell number |
| GO Biological Processes | GO:0050830 | defense response to Gram-positive bacterium |
| GO Biological Processes | GO:0051897 | positive regulation of protein kinase B signaling |
| GO Biological Processes | GO:0006886 | intracellular protein transport |
| GO Biological Processes | GO:0045444 | fat cell differentiation |
| GO Biological Processes | GO:0002758 | innate immune response-activating signaling pathway |
| GO Biological Processes | GO:1905114 | cell surface receptor signaling pathway involved in cell-cell signaling |
| GO Biological Processes | GO:0001952 | regulation of cell-matrix adhesion |
| GO Biological Processes | GO:0016032 | viral process |
| GO Biological Processes | GO:0021987 | cerebral cortex development |
| GO Biological Processes | GO:0007189 | adenylate cyclase-activating G protein-coupled receptor signaling pathway |
| GO Biological Processes | GO:0003206 | cardiac chamber morphogenesis |
| GO Biological Processes | GO:0090288 | negative regulation of cellular response to growth factor stimulus |
| GO Biological Processes | GO:0035303 | regulation of dephosphorylation |
| GO Biological Processes | GO:0060560 | developmental growth involved in morphogenesis |
| GO Biological Processes | GO:0000018 | regulation of DNA recombination |
| GO Biological Processes | GO:0051224 | negative regulation of protein transport |
| GO Biological Processes | GO:0001764 | neuron migration |
| GO Biological Processes | GO:0007160 | cell-matrix adhesion |
| GO Biological Processes | GO:1903052 | positive regulation of proteolysis involved in protein catabolic process |
| GO Biological Processes | GO:1904950 | negative regulation of establishment of protein localization |
| GO Biological Processes | GO:0008360 | regulation of cell shape |
| GO Biological Processes | GO:0061024 | membrane organization |
| GO Biological Processes | GO:0044403 | biological process involved in symbiotic interaction |
| GO Biological Processes | GO:0030216 | keratinocyte differentiation |

**6. Supplementary Table 6. 180 pathways were enriched by KEGG.**

| NO. | Category | Description | LogP | Enrichment | #GeneInGOAndHitList |
| --- | --- | --- | --- | --- | --- |
| 1 | KEGG Pathway | Pathways in cancer | -54 | 28 | 45 |
| 2 | KEGG Pathway | Lipid and atherosclerosis | -38 | 43 | 28 |
| 3 | KEGG Pathway | AGE-RAGE signaling pathway in diabetic complications | -35 | 72 | 22 |
| 4 | KEGG Pathway | Fluid shear stress and atherosclerosis | -33 | 54 | 23 |
| 5 | KEGG Pathway | Human cytomegalovirus infection | -32 | 36 | 25 |
| 6 | KEGG Pathway | Hepatitis B | -30 | 45 | 22 |
| 7 | KEGG Pathway | Kaposi sarcoma-associated herpesvirus infection | -30 | 39 | 23 |
| 8 | KEGG Pathway | Chemical carcinogenesis - receptor activation | -27 | 34 | 22 |
| 9 | KEGG Pathway | Epstein-Barr virus infection | -26 | 34 | 21 |
| 10 | KEGG Pathway | Tuberculosis | -25 | 36 | 20 |
| 11 | KEGG Pathway | Cellular senescence | -25 | 40 | 19 |
| 12 | KEGG Pathway | Hepatitis C | -25 | 40 | 19 |
| 13 | KEGG Pathway | TNF signaling pathway | -24 | 50 | 17 |
| 14 | KEGG Pathway | IL-17 signaling pathway | -23 | 56 | 16 |
| 15 | KEGG Pathway | Prostate cancer | -23 | 54 | 16 |
| 16 | KEGG Pathway | Endocrine resistance | -23 | 54 | 16 |
| 17 | KEGG Pathway | Pancreatic cancer | -23 | 65 | 15 |
| 18 | KEGG Pathway | Chagas disease | -23 | 51 | 16 |
| 19 | KEGG Pathway | Proteoglycans in cancer | -22 | 30 | 19 |
| 20 | KEGG Pathway | Hepatocellular carcinoma | -22 | 35 | 18 |
| 21 | KEGG Pathway | Pathways of neurodegeneration - multiple diseases | -22 | 17 | 24 |
| 22 | KEGG Pathway | Colorectal cancer | -22 | 57 | 15 |
| 23 | KEGG Pathway | Toxoplasmosis | -22 | 47 | 16 |
| 24 | KEGG Pathway | Small cell lung cancer | -22 | 54 | 15 |
| 25 | KEGG Pathway | Pertussis | -21 | 60 | 14 |
| 26 | KEGG Pathway | C-type lectin receptor signaling pathway | -21 | 47 | 15 |
| 27 | KEGG Pathway | Influenza A | -21 | 33 | 17 |
| 28 | KEGG Pathway | Th17 cell differentiation | -20 | 46 | 15 |
| 29 | KEGG Pathway | Measles | -20 | 38 | 16 |
| 30 | KEGG Pathway | HIF-1 signaling pathway | -20 | 45 | 15 |
| 31 | KEGG Pathway | Alzheimer disease | -20 | 18 | 21 |
| 32 | KEGG Pathway | PI3K-Akt signaling pathway | -19 | 19 | 20 |
| 33 | KEGG Pathway | Platinum drug resistance | -19 | 58 | 13 |
| 34 | KEGG Pathway | Human T-cell leukemia virus 1 infection | -19 | 25 | 17 |
| 35 | KEGG Pathway | Chemical carcinogenesis - reactive oxygen species | -19 | 25 | 17 |
| 36 | KEGG Pathway | Bladder cancer | -18 | 88 | 11 |
| 37 | KEGG Pathway | Non-alcoholic fatty liver disease | -18 | 32 | 15 |
| 38 | KEGG Pathway | Acute myeloid leukemia | -18 | 59 | 12 |
| 39 | KEGG Pathway | Salmonella infection | -18 | 22 | 17 |
| 40 | KEGG Pathway | Malaria | -17 | 72 | 11 |
| 41 | KEGG Pathway | T cell receptor signaling pathway | -17 | 41 | 13 |
| 42 | KEGG Pathway | Chronic myeloid leukemia | -17 | 52 | 12 |
| 43 | KEGG Pathway | Human papillomavirus infection | -17 | 18 | 18 |
| 44 | KEGG Pathway | Leishmaniasis | -17 | 51 | 12 |
| 45 | KEGG Pathway | EGFR tyrosine kinase inhibitor resistance | -17 | 50 | 12 |
| 46 | KEGG Pathway | Gastric cancer | -17 | 31 | 14 |
| 47 | KEGG Pathway | MAPK signaling pathway | -17 | 19 | 17 |
| 48 | KEGG Pathway | Shigellosis | -16 | 21 | 16 |
| 49 | KEGG Pathway | Inflammatory bowel disease | -16 | 56 | 11 |
| 50 | KEGG Pathway | Human immunodeficiency virus 1 infection | -16 | 23 | 15 |
| 51 | KEGG Pathway | Osteoclast differentiation | -16 | 33 | 13 |
| 52 | KEGG Pathway | Apoptosis | -16 | 31 | 13 |
| 53 | KEGG Pathway | Yersinia infection | -15 | 31 | 13 |
| 54 | KEGG Pathway | p53 signaling pathway | -15 | 49 | 11 |
| 55 | KEGG Pathway | Toll-like receptor signaling pathway | -15 | 38 | 12 |
| 56 | KEGG Pathway | Coronavirus disease - COVID-19 | -15 | 21 | 15 |
| 57 | KEGG Pathway | Transcriptional misregulation in cancer | -15 | 24 | 14 |
| 58 | KEGG Pathway | Thyroid cancer | -15 | 80 | 9 |
| 59 | KEGG Pathway | FoxO signaling pathway | -14 | 30 | 12 |
| 60 | KEGG Pathway | Non-small cell lung cancer | -14 | 46 | 10 |
| 61 | KEGG Pathway | NF-kappa B signaling pathway | -14 | 35 | 11 |
| 62 | KEGG Pathway | Breast cancer | -14 | 27 | 12 |
| 63 | KEGG Pathway | Pathogenic Escherichia coli infection | -13 | 22 | 13 |
| 64 | KEGG Pathway | Viral carcinogenesis | -13 | 21 | 13 |
| 65 | KEGG Pathway | Neurotrophin signaling pathway | -13 | 30 | 11 |
| 66 | KEGG Pathway | ErbB signaling pathway | -13 | 39 | 10 |
| 67 | KEGG Pathway | Legionellosis | -13 | 52 | 9 |
| 68 | KEGG Pathway | Endometrial cancer | -13 | 51 | 9 |
| 69 | KEGG Pathway | PD-L1 expression and PD-1 checkpoint pathway in cancer | -13 | 37 | 10 |
| 70 | KEGG Pathway | African trypanosomiasis | -13 | 71 | 8 |
| 71 | KEGG Pathway | Rheumatoid arthritis | -13 | 35 | 10 |
| 72 | KEGG Pathway | Alcoholic liver disease | -12 | 25 | 11 |
| 73 | KEGG Pathway | MicroRNAs in cancer | -12 | 15 | 14 |
| 74 | KEGG Pathway | Amoebiasis | -12 | 32 | 10 |
| 75 | KEGG Pathway | Melanoma | -12 | 41 | 9 |
| 76 | KEGG Pathway | Glioma | -12 | 39 | 9 |
| 77 | KEGG Pathway | Herpes simplex virus 1 infection | -12 | 11 | 16 |
| 78 | KEGG Pathway | Prion disease | -12 | 16 | 13 |
| 79 | KEGG Pathway | JAK-STAT signaling pathway | -12 | 22 | 11 |
| 80 | KEGG Pathway | Sphingolipid signaling pathway | -11 | 28 | 10 |
| 81 | KEGG Pathway | Thyroid hormone signaling pathway | -11 | 27 | 10 |
| 82 | KEGG Pathway | Cytokine-cytokine receptor interaction | -11 | 14 | 13 |
| 83 | KEGG Pathway | Relaxin signaling pathway | -11 | 25 | 10 |
| 84 | KEGG Pathway | Longevity regulating pathway | -11 | 33 | 9 |
| 85 | KEGG Pathway | Th1 and Th2 cell differentiation | -11 | 32 | 9 |
| 86 | KEGG Pathway | NOD-like receptor signaling pathway | -11 | 20 | 11 |
| 87 | KEGG Pathway | Chemokine signaling pathway | -11 | 19 | 11 |
| 88 | KEGG Pathway | Chemical carcinogenesis - DNA adducts | -10 | 38 | 8 |
| 89 | KEGG Pathway | Adipocytokine signaling pathway | -10 | 38 | 8 |
| 90 | KEGG Pathway | Prolactin signaling pathway | -10 | 38 | 8 |
| 91 | KEGG Pathway | Insulin resistance | -10 | 27 | 9 |
| 92 | KEGG Pathway | Drug metabolism - cytochrome P450 | -10 | 36 | 8 |
| 93 | KEGG Pathway | Amyotrophic lateral sclerosis | -10 | 12 | 13 |
| 94 | KEGG Pathway | Antifolate resistance | -9.4 | 66 | 6 |
| 95 | KEGG Pathway | Apoptosis - multiple species | -9.2 | 62 | 6 |
| 96 | KEGG Pathway | VEGF signaling pathway | -9.2 | 39 | 7 |
| 97 | KEGG Pathway | Viral protein interaction with cytokine and cytokine receptor | -9.1 | 26 | 8 |
| 98 | KEGG Pathway | Fc epsilon RI signaling pathway | -8.8 | 34 | 7 |
| 99 | KEGG Pathway | Allograft rejection | -8.8 | 52 | 6 |
| 100 | KEGG Pathway | RIG-I-like receptor signaling pathway | -8.7 | 33 | 7 |
| 101 | KEGG Pathway | Graft-versus-host disease | -8.5 | 47 | 6 |
| 102 | KEGG Pathway | Metabolism of xenobiotics by cytochrome P450 | -8.4 | 29 | 7 |
| 103 | KEGG Pathway | Cell cycle | -8.3 | 21 | 8 |
| 104 | KEGG Pathway | Drug metabolism - other enzymes | -8.3 | 29 | 7 |
| 105 | KEGG Pathway | Natural killer cell mediated cytotoxicity | -8.2 | 20 | 8 |
| 106 | KEGG Pathway | Neutrophil extracellular trap formation | -8.1 | 16 | 9 |
| 107 | KEGG Pathway | Cushing syndrome | -7.6 | 17 | 8 |
| 108 | KEGG Pathway | cAMP signaling pathway | -7.6 | 13 | 9 |
| 109 | KEGG Pathway | Necroptosis | -7.5 | 17 | 8 |
| 110 | KEGG Pathway | Renal cell carcinoma | -7.2 | 29 | 6 |
| 111 | KEGG Pathway | Epithelial cell signaling in Helicobacter pylori infection | -7.1 | 28 | 6 |
| 112 | KEGG Pathway | Growth hormone synthesis, secretion and action | -7.1 | 19 | 7 |
| 113 | KEGG Pathway | Focal adhesion | -6.7 | 13 | 8 |
| 114 | KEGG Pathway | B cell receptor signaling pathway | -6.7 | 24 | 6 |
| 115 | KEGG Pathway | Type I diabetes mellitus | -6.7 | 38 | 5 |
| 116 | KEGG Pathway | Diabetic cardiomyopathy | -6.7 | 13 | 8 |
| 117 | KEGG Pathway | Insulin signaling pathway | -6.7 | 17 | 7 |
| 118 | KEGG Pathway | Autophagy - animal | -6.6 | 16 | 7 |
| 119 | KEGG Pathway | Intestinal immune network for IgA production | -6.4 | 33 | 5 |
| 120 | KEGG Pathway | Ras signaling pathway | -6.2 | 11 | 8 |
| 121 | KEGG Pathway | Progesterone-mediated oocyte maturation | -6.1 | 19 | 6 |
| 122 | KEGG Pathway | Basal cell carcinoma | -5.8 | 26 | 5 |
| 123 | KEGG Pathway | Cytosolic DNA-sensing pathway | -5.8 | 26 | 5 |
| 124 | KEGG Pathway | Parkinson disease | -5.8 | 9.9 | 8 |
| 125 | KEGG Pathway | Asthma | -5.6 | 42 | 4 |
| 126 | KEGG Pathway | Central carbon metabolism in cancer | -5.6 | 23 | 5 |
| 127 | KEGG Pathway | Estrogen signaling pathway | -5.4 | 14 | 6 |
| 128 | KEGG Pathway | Apelin signaling pathway | -5.4 | 14 | 6 |
| 129 | KEGG Pathway | Huntington disease | -5.3 | 8.6 | 8 |
| 130 | KEGG Pathway | Signaling pathways regulating pluripotency of stem cells | -5.3 | 14 | 6 |
| 131 | KEGG Pathway | mTOR signaling pathway | -5.1 | 13 | 6 |
| 132 | KEGG Pathway | Calcium signaling pathway | -5 | 9.6 | 7 |
| 133 | KEGG Pathway | TGF-beta signaling pathway | -5 | 17 | 5 |
| 134 | KEGG Pathway | Type II diabetes mellitus | -4.9 | 29 | 4 |
| 135 | KEGG Pathway | Wnt signaling pathway | -4.9 | 12 | 6 |
| 136 | KEGG Pathway | cGMP-PKG signaling pathway | -4.9 | 12 | 6 |
| 137 | KEGG Pathway | Choline metabolism in cancer | -4.9 | 17 | 5 |
| 138 | KEGG Pathway | Hematopoietic cell lineage | -4.9 | 17 | 5 |
| 139 | KEGG Pathway | Parathyroid hormone synthesis, secretion and action | -4.7 | 15 | 5 |
| 140 | KEGG Pathway | Autoimmune thyroid disease | -4.7 | 25 | 4 |
| 141 | KEGG Pathway | Cholinergic synapse | -4.6 | 15 | 5 |
| 142 | KEGG Pathway | Leukocyte transendothelial migration | -4.6 | 14 | 5 |
| 143 | KEGG Pathway | Serotonergic synapse | -4.6 | 14 | 5 |
| 144 | KEGG Pathway | Glutathione metabolism | -4.5 | 23 | 4 |
| 145 | KEGG Pathway | Viral myocarditis | -4.5 | 22 | 4 |
| 146 | KEGG Pathway | Steroid hormone biosynthesis | -4.4 | 22 | 4 |
| 147 | KEGG Pathway | Longevity regulating pathway - multiple species | -4.4 | 21 | 4 |
| 148 | KEGG Pathway | Rap1 signaling pathway | -4.3 | 9.4 | 6 |
| 149 | KEGG Pathway | Oocyte meiosis | -4.3 | 13 | 5 |
| 150 | KEGG Pathway | Dopaminergic synapse | -4.3 | 12 | 5 |
| 151 | KEGG Pathway | Systemic lupus erythematosus | -4.2 | 12 | 5 |
| 152 | KEGG Pathway | PPAR signaling pathway | -4.1 | 18 | 4 |
| 153 | KEGG Pathway | Phospholipase D signaling pathway | -4 | 11 | 5 |
| 154 | KEGG Pathway | Retrograde endocannabinoid signaling | -4 | 11 | 5 |
| 155 | KEGG Pathway | Adrenergic signaling in cardiomyocytes | -4 | 11 | 5 |
| 156 | KEGG Pathway | Bile secretion | -3.8 | 15 | 4 |
| 157 | KEGG Pathway | Tyrosine metabolism | -3.7 | 27 | 3 |
| 158 | KEGG Pathway | GnRH signaling pathway | -3.7 | 14 | 4 |
| 159 | KEGG Pathway | Fc gamma R-mediated phagocytosis | -3.6 | 14 | 4 |
| 160 | KEGG Pathway | Tryptophan metabolism | -3.5 | 23 | 3 |
| 161 | KEGG Pathway | Cocaine addiction | -3.3 | 20 | 3 |
| 162 | KEGG Pathway | AMPK signaling pathway | -3.3 | 11 | 4 |
| 163 | KEGG Pathway | Platelet activation | -3.2 | 11 | 4 |
| 164 | KEGG Pathway | Regulation of lipolysis in adipocytes | -3.2 | 18 | 3 |
| 165 | KEGG Pathway | Thermogenesis | -3.1 | 7.1 | 5 |
| 166 | KEGG Pathway | Spinocerebellar ataxia | -3 | 9.2 | 4 |
| 167 | KEGG Pathway | Cell adhesion molecules | -2.9 | 8.8 | 4 |
| 168 | KEGG Pathway | Long-term potentiation | -2.9 | 15 | 3 |
| 169 | KEGG Pathway | Retinol metabolism | -2.9 | 14 | 3 |
| 170 | KEGG Pathway | Renin secretion | -2.9 | 14 | 3 |
| 171 | KEGG Pathway | Oxytocin signaling pathway | -2.9 | 8.5 | 4 |
| 172 | KEGG Pathway | Hippo signaling pathway | -2.9 | 8.4 | 4 |
| 173 | KEGG Pathway | Mitophagy - animal | -2.9 | 14 | 3 |
| 174 | KEGG Pathway | Protein processing in endoplasmic reticulum | -2.7 | 7.7 | 4 |
| 175 | KEGG Pathway | Peroxisome | -2.7 | 12 | 3 |
| 176 | KEGG Pathway | Inflammatory mediator regulation of TRP channels | -2.5 | 10 | 3 |
| 177 | KEGG Pathway | Melanogenesis | -2.4 | 9.7 | 3 |
| 178 | KEGG Pathway | Pancreatic secretion | -2.4 | 9.7 | 3 |
| 179 | KEGG Pathway | Neuroactive ligand-receptor interaction | -2.3 | 4.5 | 5 |
| 180 | KEGG Pathway | Vascular smooth muscle contraction | -2.1 | 7.3 | 3 |

**6. Supplementary Table 6.** **Degree information for PPI.**

| NO | name | Degree |
| --- | --- | --- |
| 1 | AKT1 | 25 |
| 2 | RELA | 24 |
| 3 | STAT3 | 23 |
| 4 | MAPK1 | 20 |
| 5 | MAPK14 | 20 |
| 6 | TNF | 19 |
| 7 | TP53 | 19 |
| 8 | IL6 | 18 |
| 9 | IL10 | 15 |
| 10 | MAPK8 | 15 |
| 11 | NFKB1 | 15 |
| 12 | IL1B | 14 |
| 13 | MYC | 14 |
| 14 | IL2 | 14 |
| 15 | CXCL8 | 13 |
| 16 | IL4 | 13 |
| 17 | ESR1 | 13 |
| 18 | CTNNB1 | 12 |
| 19 | CDKN1A | 11 |
| 20 | CASP3 | 11 |
| 21 | RB1 | 11 |
| 22 | IL1A | 10 |
| 23 | BCL2 | 10 |
| 24 | MMP1 | 9 |
| 25 | CCL2 | 9 |
| 26 | CYCS | 9 |
| 27 | MDM2 | 9 |
| 28 | PPARG | 8 |
| 29 | CXCL10 | 8 |
| 30 | CD40LG | 8 |
| 31 | AR | 8 |
| 32 | MMP2 | 7 |
| 33 | IFNG | 7 |
| 34 | VEGFA | 7 |
| 35 | CDK2 | 7 |
| 36 | MTOR | 7 |
| 37 | NOS2 | 7 |
| 38 | RPS6KB1 | 7 |
| 39 | SYK | 6 |
| 40 | IGFBP3 | 6 |
| 41 | PTGS2 | 6 |
| 42 | BAD | 6 |
| 43 | RXRB | 6 |
| 44 | IGF2 | 5 |
| 45 | F2 | 5 |
| 46 | ICAM1 | 4 |
| 47 | CYP3A4 | 4 |
| 48 | CYP1A2 | 4 |
| 49 | PCNA | 4 |
| 50 | BAX | 4 |
| 51 | VCAM1 | 3 |
| 52 | HMOX1 | 3 |
| 53 | PIK3CG | 3 |
| 54 | CAT | 3 |
| 55 | AHR | 3 |
| 56 | SELE | 2 |
| 57 | HPGDS | 2 |
| 58 | MPO | 2 |
| 59 | ELANE | 2 |
| 60 | MAOB | 2 |
| 61 | HSD3B2 | 2 |
| 62 | UGT1A1 | 2 |
| 63 | PPARD | 2 |
| 64 | CHEK2 | 2 |
| 65 | CASP7 | 2 |
| 66 | EDNRA | 2 |
| 67 | ERBB3 | 2 |
| 68 | PON1 | 1 |
| 69 | PRSS1 | 1 |
| 70 | PNMT | 1 |
| 71 | NFE2L2 | 1 |
| 72 | GSTP1 | 1 |
| 73 | GSTM2 | 1 |
| 74 | GSTM1 | 1 |
| 75 | SERPINE1 | 1 |
| 76 | DPP4 | 1 |
| 77 | XDH | 1 |
| 78 | ESRRA | 1 |
| 79 | PPP3CA | 1 |
| 80 | CXCL11 | 1 |
| 81 | CA2 | 1 |
| 82 | BRAF | 1 |
| 83 | GLI2 | 1 |
| 84 | ADRB2 | 1 |
